# Peptide barcoding for establishment of new types of genotype–phenotype linkages

**DOI:** 10.1371/journal.pone.0215993

**Published:** 2019-04-23

**Authors:** Kana Miyamoto, Wataru Aoki, Yuta Ohtani, Natsuko Miura, Shunsuke Aburaya, Yusei Matsuzaki, Kaho Kajiwara, Yoshinori Kitagawa, Mitsuyoshi Ueda

**Affiliations:** 1 Division of Applied Life Sciences, Graduate School of Agriculture, Kyoto University, Kitashirakawa Oiwake-cho, Sakyo-ku, Kyoto, Japan; 2 Core Research for Evolutional Science and Technology (CREST), Japan Science and Technology Agency (JST), 7 Goban-cho, Chiyoda-ku, Tokyo, Japan; 3 Kyoto Integrated Science & Technology Bio-Analysis Center, 134 Chudoji Minamimachi, Simogyo-ku, Kyoto, Japan; 4 Graduate School of Life and Environmental Sciences, Osaka Prefecture University, 1–1 Gakuen-cho, Naka-ku, Sakai, Osaka, Japan; 5 Japan Society for the Promotion of Science, 5-3-1 Kojimachi, Chiyoda-ku, Tokyo, Japan; Helsingin Yliopisto, FINLAND

## Abstract

Measuring binding properties of binders (e.g., antibodies) is essential for developing useful experimental reagents, diagnostics, and pharmaceuticals. Display technologies can evaluate a large number of binders in a high-throughput manner, but the immobilization effect and the avidity effect prohibit the precise evaluation of binding properties. In this paper, we propose a novel methodology, peptide barcoding, to quantitatively measure the binding properties of multiple binders without immobilization. In the experimental scheme, unique peptide barcodes are fused with each binder, and they represent genotype information. These peptide barcodes are designed to have high detectability for mass spectrometry, leading to low identification bias and a high identification rate. A mixture of different peptide-barcoded nanobodies is reacted with antigen-coated magnetic beads in one pot. Peptide barcodes of functional nanobodies are cleaved on beads by a specific protease, and identified by selected reaction monitoring using triple quadrupole mass spectrometry. To demonstrate proof-of-principle for peptide barcoding, we generated peptide-barcoded anti-CD4 nanobody and anti-GFP nanobody, and determined whether we could simultaneously quantify their binding activities. We showed that peptide barcoding did not affect the properties of the nanobodies, and succeeded in measuring the binding activities of these nanobodies in one shot. The results demonstrate the advantages of peptide barcoding, new types of genotype–phenotype linkages.

## Introduction

Binders such as antibodies are biologics that are useful for application as experimental reagents, diagnostics, and pharmaceuticals. To measure the binding properties of binders, one approach is to purify each binder and measure its kinetic parameters using surface plasmon resonance or ELISA. This approach enables precise measurement of kinetic parameters, although its throughput is low. An alternative approach is to use various display technologies: phage display [1,2], bacterial display [3], yeast display [4,5], mammalian display [6], or ribosome display [7]. In these display technologies, a gene encoding a chimeric protein of a binder and a cell surface protein is designed and introduced into phages or cells. The produced chimeric proteins are immobilized at the cell surface, establishing genotype–phenotype linkages at the single-cell level. Using randomized display libraries, we can isolate various binders after several cycles of panning assays and genetic sequencing of positive clones. Display technologies can evaluate a large number of binders in a high-throughput manner, and have been used to isolate functional antibodies including various antibody medicines [8,9].

Although display technologies have various advantages, they also have some drawbacks, one of which is the immobilization effect. The immobilization of target proteins on the cell surface is necessary to establish genotype–phenotype linkages, but this can affect protein functions. For example, an anti-CaM scFv (*K*_*d*_ = 1 nM) maturated by yeast display lost its binding activity after conversion to a soluble form [10]. Another example is that the orientation of displayed proteins affected the binding activity of an anti-CD3ε scFv, which showed threefold-higher affinity with the free N-terminus [11]. Furthermore, it is known that the immobilization could have negative effects on the accessibility to antigens [12]. Another drawback is the avidity effect. For example, typical bacterial display systems showed cell surface display of target proteins at a level of 10^4^ [13], and yeast display at a level of 10^4^–10^5^ [14]. The avidity effect could result in the isolation of binders with relatively weak affinity, particularly when binders are selected against oligomeric antigens [15].

Several research groups have developed methodologies to evaluate free binders without immobilization. For example, Shembekar *et al*. developed a droplet microfluidic device in which each immune cell isolated from an immunized animal is compartmentalized in droplets and the binding activities of secreted antibodies are evaluated by dual-color normalized fluorescence readout [16]. This research presented a high-throughput droplet microfluidic approach to detect the activities of free antibodies, but the signal/noise ratio and the quantitativity are not high. Another approach is to identify functional antibodies by shotgun proteomics [17–19]. This approach is high-throughput, but there are several issues to be resolved. First, it is difficult to quantify the kinetic parameters of antibodies due to the low quantitativity of shotgun proteomics. Second, high sequence homology of binder ensembles could lead to ambiguous identification of binders. Finally, the existence of peptides with low detectability for mass spectrometry leads to identification bias.

**Fig 1:**
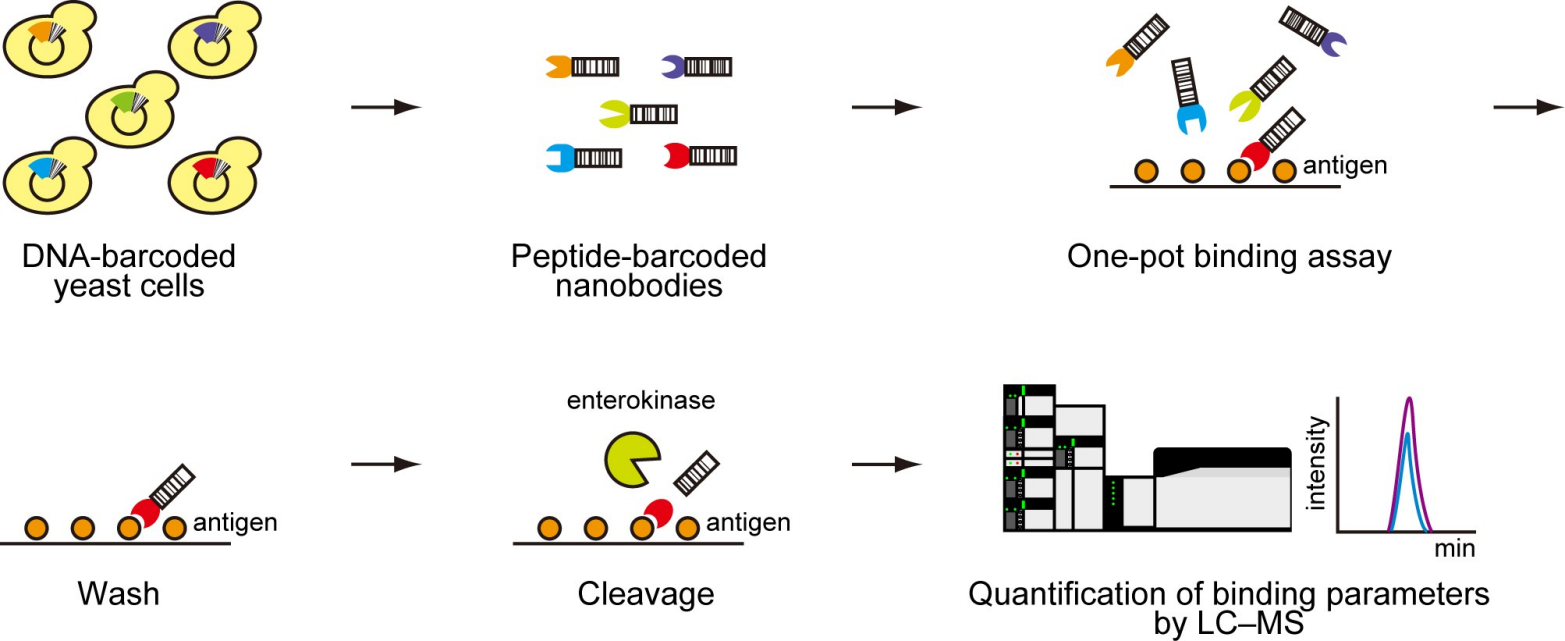
Peptide barcoding for new types of genotype–phenotype linkages. A DNA fragment encoding a unique peptide barcode is fused with the C-terminal of each nanobody gene. Transformed *Pichia pastoris* produces nanobodies fused with peptide barcodes. These nanobodies are mixed with antigen-coated magnetic beads in one pot, and non-specific nanobodies are washed out. Peptide barcodes are cleaved by a specific protease (e.g., enterokinase) and eluted peptide barcodes are quantified by mass spectrometry.

In this study, we attempted to develop a novel methodology that can measure the binding activities of free antibodies with low identification bias, high quantitativity, and potential scalability. The idea is to use small and unique peptide barcodes with high detectability for mass spectrometry. The experimental scheme is shown in Fig 1. DNA sequences encoding designer peptide barcodes are fused with nanobodies, which consist of a V_H_H domain of camelid natural single-domain antibody [20]. These chimeric genes are introduced into the methylotrophic yeast *Pichia pastoris*, and peptide-barcoded nanobodies are produced as secreted proteins. A mixture of different nanobodies is reacted with antigen-coated magnetics beads in one pot, and non-specific nanobodies are washed out. Peptide barcodes of functional nanobodies are cleaved by a specific protease (e.g., enterokinase in this study). Finally, eluted peptide barcodes are identified by selected reaction monitoring (SRM) using triple quadrupole mass spectrometry, which has high sensitivity, specificity, and quantitativity.

This strategy has several advantages. First, peptide barcodes represent genotype information of nanobodies, hence immobilization on huge carriers such as phages or cells is not necessary. Peptide barcodes are very small and expected not to affect the properties of proteins. Second, this experimental scheme enables highly sensitive, specific, and quantitative analysis of nanobodies. We analyze only pre-designed peptide barcodes with high detectability for mass spectrometry, leading to low identification bias and a high identification rate. Furthermore, a decrease of peptide variation by using a specific protease leads to the sensitive detection of peptide barcodes. The third advantage is that we can simultaneously evaluate the productivity of each nanobody when we analyze the mixture of nanobodies before the one-pot binding assay. This advantage could simplify the process of developing binders.

In this paper, we demonstrate proof-of-principle for peptide barcoding using nanobodies as model binders. We generated peptide-barcoded anti-CD4 nanobody and anti-GFP nanobody, and determined whether we could simultaneously quantify their binding activities. We showed that peptide barcoding did not affect the properties of the nanobodies, and succeeded in measuring their binding activities in one shot. These results demonstrated the advantages of peptide barcoding and new types of genotype–phenotype linkages, and we expect that this experimental scheme can be applied to other experiments such as the directed evolution of binders in the future.

## Results

### Production of nanobodies fused with peptide barcodes

**Fig 2:**
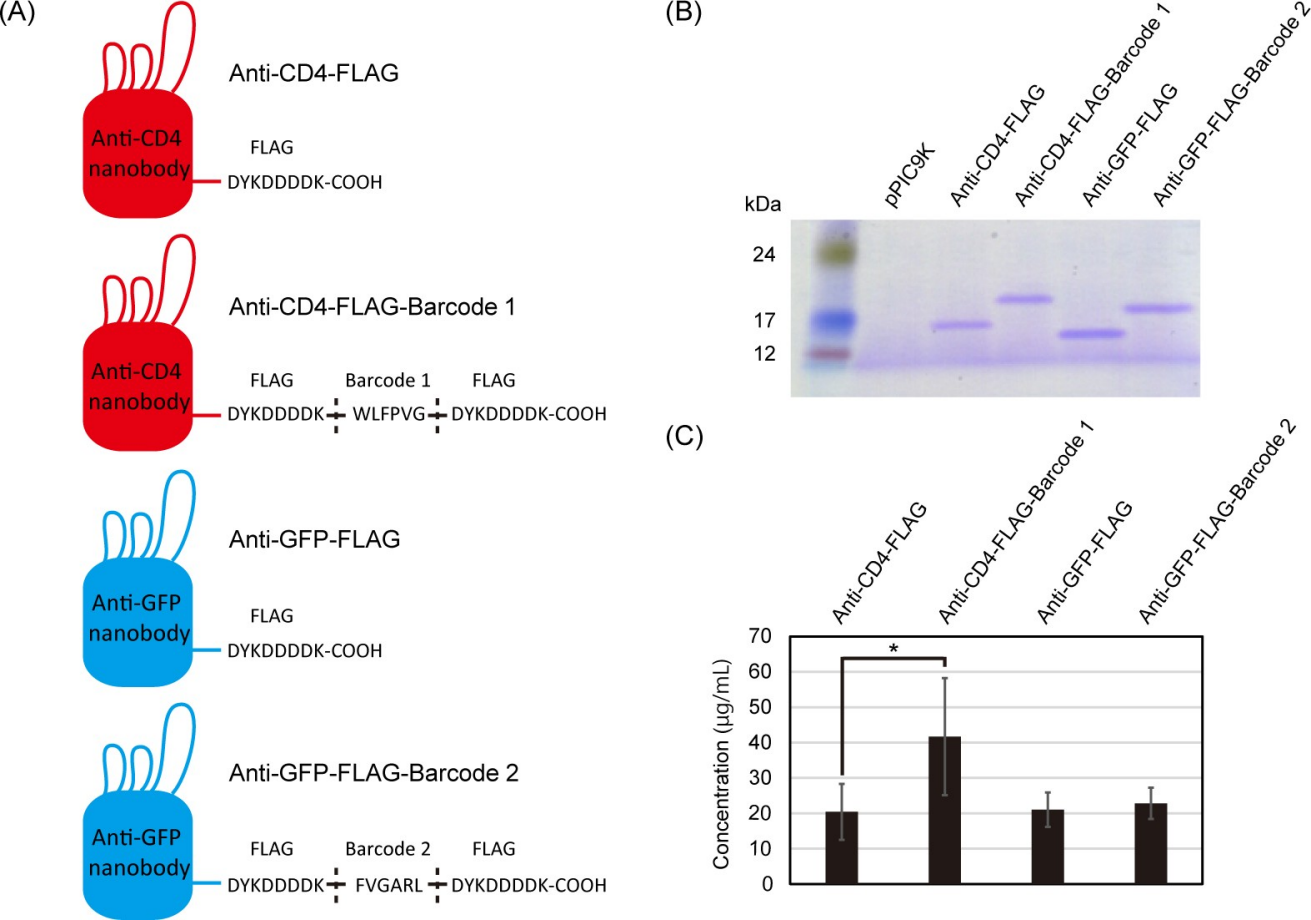
Generation of functional nanobodies fused with peptide barcodes. (A) Design of nanobodies used in this study. A unique peptide barcode was fused at the C-terminal of each nanobody. These peptide barcodes were designed to have high detectability for LC–MS analysis. (B) SDS-PAGE analysis of the nanobodies produced by *Pichia pastoris*. The culture medium was applied to an SDS-PAGE gel, and proteins were stained by Coomassie Brilliant Blue. pPIC9K is a backbone vector used as a negative control. (C) The productivity of the nanobodies by *P*. *pastoris*. Each value represents the mean ± standard deviation of at least five biological replicates. Statistical analysis was performed by *t*-test. An asterisk indicates a significant difference (p < 0.05).

In this study, we designed four nanobodies based on an anti-CD4 nanobody [21] and an anti-GFP nanobody [22] developed in previous studies. Among the designed nanobodies, two are fused with a FLAG tag sequence, and the others are fused with a FLAG tag sequence and a unique peptide barcode composed of six amino acid residues (Barcode 1 and Barcode 2, Fig 2A). These peptide barcodes represent genotype information of each nanobody and will be quantified by LC–MS. The design principles of the peptide barcodes are described in detail in the Materials & Methods section.

We constructed plasmids encoding these nanobodies (S1 Fig) and introduced them into the methylotrophic yeast *P*. *pastoris*. The transformants were cultured with media containing methanol as an inducer, and we confirmed the successful production of all of the designed nanobodies (Fig 2B). There was a small difference in the productivity between anti-CD4-FLAG nanobody and anti-CD4-FLAG-Barcode 1 nanobody (Fig 2C), but this might have been derived from copy number variation of the *P*. *pastoris* transformants [23].

### Evaluation of binding properties of the barcoded nanobodies

**Fig 3:**
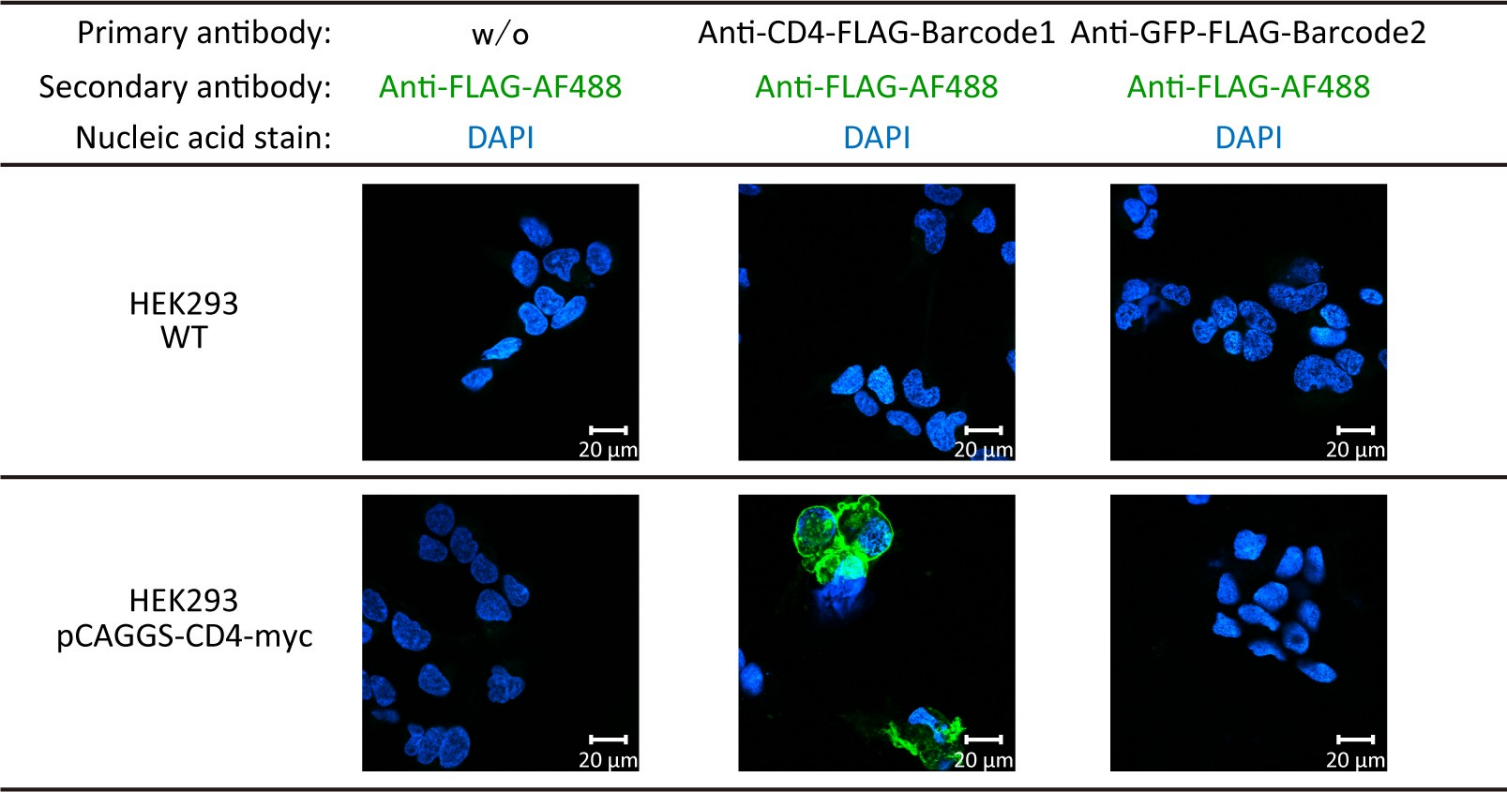
Immunofluorescence staining of CD4. Nuclei were stained by 4′,6-diamidino-2-phenylindole (DAPI, blue). CD4 was stained by each nanobody purified with anti-FLAG gels as a primary antibody and anti-DDDDK-tag mAb Alexa Fluor 488 (anti-FLAG-AF488) as a secondary antibody (green). CD4 was specifically stained by the anti-CD4-FLAG-Barcode 1. Wild-type HEK293 cells were used as negative control cells. Scale bars indicate 20 μm.

We investigated whether the addition of the peptide barcodes affects the properties of the nanobodies. First, we quantified kinetic parameters of the nanobodies by surface plasmon resonance with a sensor chip coated with CD4 or GFP. All of the nanobodies showed specific binding to each antigen, and there were no significant differences in kinetic parameters between the nanobodies with or without the peptide barcodes (Table 1). Second, we carried out immunofluorescence staining of CD4 using the nanobodies. The results showed that anti-CD4-FLAG-Barcode 1 recognized CD4 on the surface of HEK293 cells, but anti-GFP-FLAG-Barcode 2 did not (Fig 3). These results indicate that the addition of the peptide barcodes did not affect the properties of the nanobodies.

**Table 1:** Kinetic parameters of the nanobodies measured by surface plasmon resonance. Each value represents the mean ± standard deviation of three technical replicates.

Nanobodies	Antigen	*k*_*on*_ (M^−1^s^−1^)	*k*_*off*_ (s^−1^)	*K*_*D*_ (nM)
Anti-CD4-FLAG	CD4	(6.8 ± 2.3) × 10^4^	(5.4 ± 1.5) × 10^−3^	42 ± 17
Anti-CD4-FLAG-Barcode 1	CD4	(7.7 ± 0.6) × 10^4^	(4.0 ± 0.1) × 10^−3^	52 ± 3
Anti-GFP-FLAG	GFP	(1.1 ± 0.3) × 10^6^	(4.8 ± 0.2) × 10^−4^	0.44 ± 0.01
Anti-GFP-FLAG-Barcode 2	GFP	(1.4 ± 0.6) ×10^6^	(1.6 ± 1.8) × 10^−4^	0.40 ± 0.12

### Characterization of binding activities of the nanobodies by mass spectrometry

**Fig 4:**
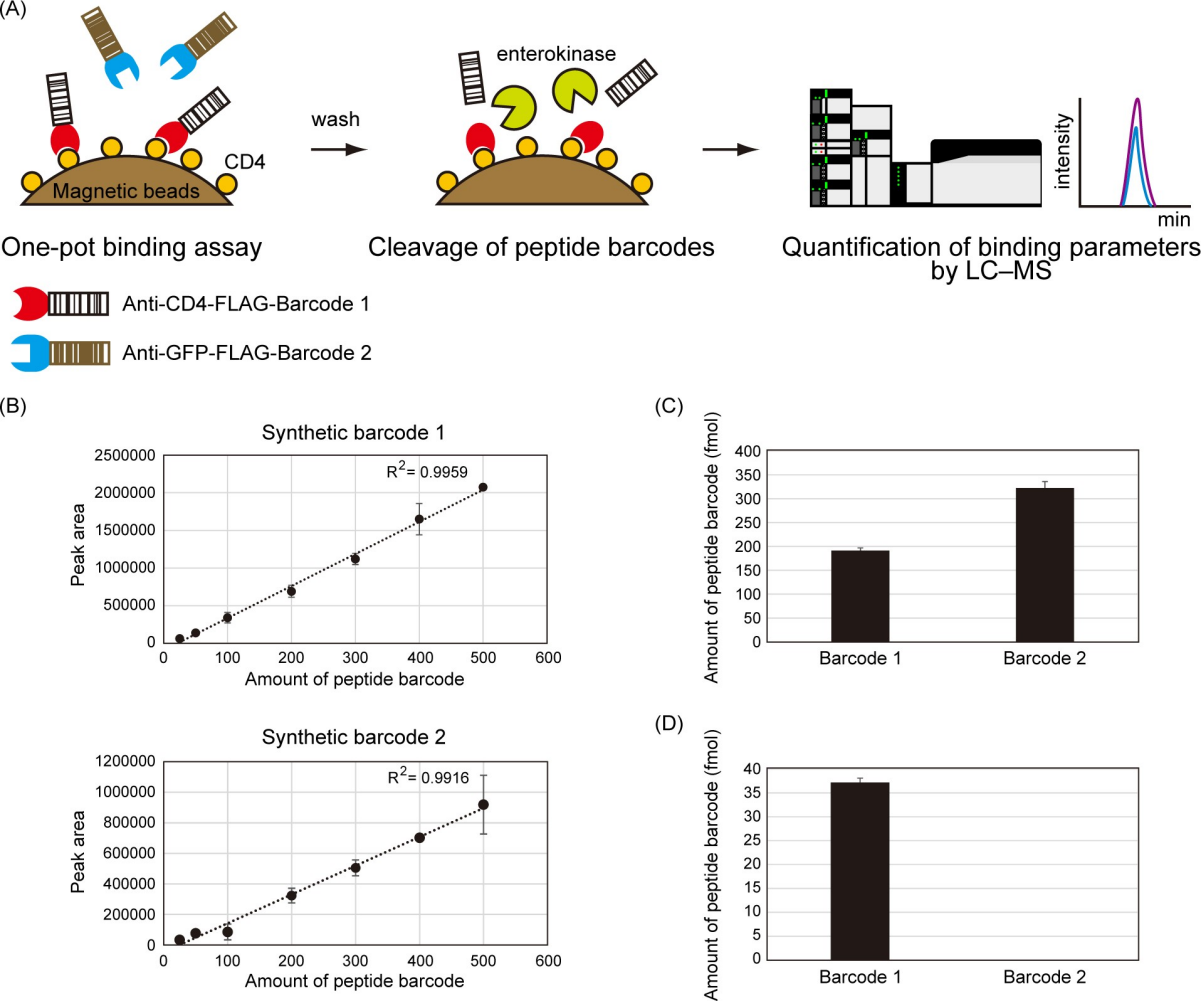
Quantification of binding activities of the barcoded nanobodies by LC–MS. (A) Experimental scheme to quantify the binding activities of the nanobodies by LC–MS. The barcoded nanobodies are mixed with CD4-coated magnetic beads in one pot, and non-specific nanobodies are washed out. The peptide barcodes are cleaved on beads by a specific protease, enterokinase. Then, eluted peptide barcodes are quantified by mass spectrometry for the evaluation of binding activities. (B) The quantitativity of SRM of each peptide barcode. Synthetic peptide barcodes were serially diluted and quantified by LCMS-8060. Each peptide barcode was successfully analyzed with high sensitivity and quantitativity. Each value represents the mean ± standard deviation of three technical replicates. (C) Quantification of each peptide-barcoded nanobody. The peptide barcodes were cleaved from 250 fmol of each nanobody, and quantified by LCMS-8060. Each value represents the mean ± standard deviation of three independent experiments. (D) Quantification of the binding strength of the peptide-barcoded nanobodies. The amount of nanobodies bound on the CD4-coated magnetic beads was quantified by LCMS-8060. The peptide barcode fused with anti-CD4 nanobody was specifically detected. Each value represents the mean ± standard deviation of three independent experiments.

We designed a proof-of-principle experiment to show that peptide barcoding can be a useful technique to measure the binding properties of multiple binders in a single experiment (Fig 4A). First, we prepared an equimolar mixture of anti-CD4-FLAG-Barcode 1 and anti-GFP-FLAG-Barcode 2. The mixture was reacted with CD4-coated magnetic beads in one pot, and non-specific binders were washed out. Then, the peptide barcodes were cleaved on beads by enterokinase. The eluted peptide barcodes were quantified by SRM for evaluation of the binding properties.

To validate the sensitivity and the quantitativity of SRM analysis, we quantified serially diluted synthetic peptide barcodes. The results showed that SRM analysis of the designer peptide barcodes was highly sensitive and quantitative (Fig 4B). As a positive control experiment, we cleaved the peptide barcodes of anti-CD4-FLAG-Barcode 1 (250 fmol) or anti-GFP-FLAG-Barcode 2 (250 fmol), and quantified them by LC–MS. As a result, we successfully quantified these peptide barcodes with minimal loss during sample preparation procedures (Fig 4C). Next, we carried out a one-pot binding assay using the CD4-coated magnetic beads, and quantified the eluted peptide barcodes from the beads. As a result of the proof-of-principle experiment, we found that the peptide barcode derived from anti-CD4-FLAG-Barcode 1 was specifically detected by LC–MS (Fig 4C). These results indicated that peptide barcoding technology enabled one-shot quantitative measurement of the binding properties of multiple binders.

## Discussion

High-throughput evaluation of the binding properties of non-immobilized free binders can be a valuable approach to develop good binders. In this paper, we propose a novel strategy for this, peptide barcoding. In this strategy, we generate a binder fused with a unique peptide barcode, which includes genotype information, and the simultaneous detection of peptide barcodes by LC–MS enables the high-throughput evaluation of the binding properties of multiple free binders. This approach has several advantages. First, peptide barcoding is devoid of adverse effects derived from the immobilization effect. Conventional display technologies require the immobilization of binders on the host cell surface. This immobilization on large carriers could affect the binding properties of binders [10,11] and the accessibility to antigens [12]. Peptide barcodes are very small and expected to have less adverse effects. In fact, our results showed that peptide barcoding did not affect the binding properties of the nanobodies (Fig 3 and Table 1). However, it will be necessary to obtain definitive evidence on whether peptide barcoding affects the properties of nanobodies by using peptide barcodes with a wide range of chemical properties. The second advantage of peptide barcoding is the improvement of detection sensitivity. In our approach, we only detect pre-designed peptide barcodes confirmed to possess high detection sensitivity for LC–MS, leading to the possibility of identifying rare binders. The third advantage is the low identification bias, which is also derived from the use of pre-designed peptide barcodes. In previous studies using shotgun proteomics to identify functional binders [17–19], tryptic digests of binder ensembles are introduced to LC–MS. Such an experimental scheme is very simple, but suffers from identification bias due to the existence of peptides with low ionization and fragmentation efficiency. Furthermore, high sequence homology of binder ensembles leads to ambiguous identification of functional binders. In contrast, our approach detects only peptide barcodes that are pre-designed to have high ionization and fragmentation efficiency and 1:1 genotype–phenotype matching. These characteristics result in the unambiguous identification of functional binders. The fourth advantage is the ability to simultaneously evaluate the productivity of binders. In our experimental scheme, we used nanobodies secreted by *P*. *pastoris*. Hence, we can simultaneously evaluate the productivity and binding properties by LC–MS, enabling the simplification of procedures of binder generation.

Although peptide barcoding can be a novel and promising way to generate binders, there are some issues to be resolved. First, to make the peptide barcoding strategy scalable, it is necessary to develop a high-throughput method to synthesize large libraries encoding chimeric proteins of mutant nanobodies and unique peptide barcodes. One promising approach for this is DropSynth, which is a scalable, low-cost methodology to synthesize thousands of genes in a multiplex manner [24]. DropSynth uses DNA-barcoded beads to pull-down target oligonucleotides, and assembles target genes in water-in-oil emulsions. Using DropSynth, it might be possible to generate thousands of peptide-barcoded nanobody libraries in a simple manner. The second issue is how to design peptide barcodes with high ionization and fragmentation efficiency in a scalable manner. In our opinion, utilization of the SRMAtlas database [25–27] is the easiest way to solve this issue. The SRMAtlas database includes over 100,000 SRM transitions. These transitions are already optimized for sensitive detection using triple quadrupole mass spectrometers. Constructing chimeric proteins of nanobodies and peptides evaluated in the SRMAtlas database, it will be possible to easily construct large peptide-barcoded nanobody libraries.

Recently, Seeger’s group initiated a similar study entitled Engineered Peptide Barcodes for In-Depth Analyses of Binding Protein Ensembles in bioRxiv [28]. Seeger’s group described a similar experimental scheme in which nanobodies fused with peptide barcodes (called flycodes) are generated by *Escherichia coli*, and their binding properties are directly evaluated by mass spectrometry without adverse immobilization effects. They succeeded in obtaining a functional nanobody against major outer membrane protein (MOMP) of the human pathogen *Legionella pneumophila*, in a cellular context where MOMP is embedded in dense lipopolysaccharides. This result also suggested that the evaluation of free nanobodies without the immobilizing effect is a promising approach to generate functional binders. Our approach and Seeger’s approach are different in some regards. First, Seeger’s group used randomized peptide barcodes expected to have high ionization and fragmentation efficiency, and detected them using an Orbitrap mass spectrometer. This approach is scalable, but could cause some identification bias because the properties of peptide barcodes are not precisely evaluated in advance. In contrast, our approach used pre-designed peptide barcodes with high sensitivity and quantitativity, and detected them by using a triple quadrupole mass spectrometer (described in detail in the Materials & Methods section). This approach enables absolute quantification of peptide barcodes, and is expected to be good at more precise evaluation of binding properties. Another difference is the host cells used to produce nanobodies. Seeger’s group used *E*. *coli*, which is a good host because of the ease of handling and library generation. In contrast, our group used secretory production by *P*. *pastoris*, which possesses eukaryotic folding machinery. These two studies have different advantages, and it will be important to select an appropriate approach depending on the experimental purpose.

In conclusion, we proposed peptide barcoding for the development of new types of genotype–phenotype linkages, and its application to directly evaluating the binding properties of nanobodies without immobilization. Our approach should enable high-throughput generation of binders for good experimental reagents, diagnostics, and pharmaceuticals.

## Materials and methods

### Generation of nanobodies fused with peptide barcodes

Anti-CD4 nanobody and anti-GFP nanobody were synthesized (Integrated DNA Technologies, Coralville, IA, USA). Full sequences of each plasmid are shown in S1 Fig.

Peptide barcodes used in this study were designed as follows. Ionization efficiency of the peptides greatly varies depending on their length and constituent amino acid residues [29–31]. While excessively short- or long-chain peptides show low ionization efficiency, peptides that have 6–16 residues show high ionization efficiency [30]. Ionization efficiency also depends on the types of amino acid residues constituting the peptides [30,31]. For example, residues having amino groups such as lysine and arginine are prone to be positively charged, so they increase the ionization efficiency. Glycine and proline residues increase the ionization efficiency, while histidine decreases it [30]. Cysteine and methionine are easily oxidized, and asparagine, serine, and threonine can be glycosylated; hence, these residues are not suitable for SRM analysis that analyzes predetermined targets. Furthermore, the retention time of peptides is greatly affected by the retention coefficients of constituent amino acids [32]. Since co-elution causes a matrix effect, the efficient separation of peptides is necessary for accurate and sensitive analysis [33,34]. Considering these factors, we chose nine amino acids (A, F, G, K, L, P, R, V, and W) as constituents. We designed two peptide barcodes to have different molecular weights: barcode 1, WLFPVG-DYKDDDDK; and barcode 2, FVGARL-DYKDDDDK. These barcodes were fused at the C-terminals of anti-CD4 and anti-GFP nanobodies, respectively.

Plasmids encoding nanobodies were digested by SacI (Toyobo, Osaka, Japan), purified by MinElute PCR Purification Kit (QIAGEN, Hilden, Germany), and introduced into the *P*. *pastoris* GS115 strain by Frozen EZ Yeast Transformation II Kit (Zymo Research, Irvine, CA, USA). The transformants were selected on MD solid medium (1.34% w/v yeast nitrogen base w/o amino acids, 2% w/v D-glucose, and 2% w/v agar). The selected colonies were cultured in 20 mL of BMGY medium (1% w/v yeast extract, 2% w/v peptone, 1% w/v glycerol, 0.1 M potassium phosphate buffer pH 6.0, 2.68% w/v yeast nitrogen base w/o amino acids, and 400 μg/mL biotin) at 30°C and 250 rpm for 48 h, and the grown cells were cultured in 10 mL of BMMY medium (1% w/v yeast extract, 2% w/v peptone, 0.1 M potassium phosphate buffer pH 6.0, 2.68% w/v yeast nitrogen base w/o amino acids, 400 μg/mL biotin, and 0.5% v/v methanol) at 30°C and 250 rpm for 24 h. After the cultivation, BMMY media were centrifuged and filtered using 0.22-μm filters. The supernatants were analyzed by SDS-PAGE to confirm the production of nanobodies. Ten milliliters of the supernatants were concentrated by using Amicon Ultra-15 Centrifugal Filters Ultracel-3K (Merck Millipore, Burlington, MA, USA) at 8000 g for 60 min. Ten milliliters of PBS was added to the Amicon Ultracel-3k unit, and centrifuged at 8000 g for 60 min. This buffer replacement procedure was repeated twice.

### Surface plasmon resonance

Kinetic parameters were measured using Biacore T-200 (GE Healthcare, Chicago, IL, USA). Recombinant human sCD4 CF (R&D Systems, Minneapolis, MN, USA) and recombinant GFP (ProSpec, Rehovot, Israel) were immobilized on Series S Sensor Chip CM5 (GE Healthcare) to be 2486.9 RU and 1189.8 RU, respectively. Anti-CD4 nanobodies were diluted with HBS-EP buffer (0.01 M HEPES pH 7.4, 0.15 M NaCl, 3 mM EDTA, and 0.005% v/v Surfactant P20) to be 0.2, 0.4, 0.6, 0.8, and 1.0 μM, and anti-GFP nanobodies to be 0.5, 1, 5, 10, and 50 nM. Flow rate, contact time, and dissociation time were 30 μL/min, 120 s, and 120 s, respectively. The CD4-coated chip was regenerated by 10 mM NaOH (flow rate 30 μL/min and contact time 30 s), and the GFP-coated chip by 50 mM NaOH (flow rate 30 μL/min and contact time 30 s).

### Immunofluorescence staining

pCAGGS-CD4-myc was a gift from Jacob Yount (Addgene plasmid #58537; http://n2t.net/addgene:58537; RRID: Addgene_58537) [35]. HEK293 cells were transfected with a pCAGGS-CD4-myc plasmid (S1 Fig) by using Xfect Transfection Reagent (Takara Bio Inc., Shiga, Japan), and cultivated in DMEM Low Glucose (Nacalai Tesque, Kyoto, Japan) for 1 day. The transfected cells were transferred onto cover glasses coated with poly-l-lysine hydrobromide (Sigma-Aldrich, St. Louis, MO, USA), and cultured in DMEM Low Glucose for 1–2 days. After removal of the culture medium, the cells were fixed with 4% paraformaldehyde for 30 min at room temperature. The fixed cells were rinsed with PBS three times and blocked with PBS containing 10% FBS (GE Healthcare) for 30 min at room temperature. Anti-CD4 nanobodies (2.7 μg/ml) or anti-GFP nanobodies (3.3 μg/ml) were incubated with the fixed cells for 90 min. The cells were rinsed with PBS three times and washed with PBS for 5 min three times. Anti-DDDDK-tag mAb Alexa Fluor 488 (MBL Co., Ltd., Aichi, Japan) was diluted to be 1 μg/mL with PBS containing 10% FBS, and incubated with the cells for 1 h at room temperature. The cells were rinsed with PBS three times and washed with PBS for 5 min three times. Nuclei were stained with 1 μg/mL 4′,6′-diamidino-2-phenylindole dihydrochloride (DAPI; Nacalai Tesque, Kyoto, Japan) for 1 min. The stained cells were visualized using a confocal laser scanning fluorescence microscope, LSM700 (Carl Zeiss, Oberkochen, Germany).

### Precipitation of nanobodies by antigen–antibody interaction

Recombinant CD4 (0.1 mg/mL) was mixed with 30 μl of NHS-activated Magnetic Beads (Thermo Scientific, Rockford, IL, USA), which was pre-washed with 1 mM ice-cold HCl. After the reaction, the beads were washed with 0.1 M glycine-HCl (pH 2.0) twice and distilled water once, and blocked with 3 M ethanolamine (pH 9.0) for 2 h at room temperature. After the blocking, the magnetic beads were suspended with 30 μl of 50 mM borate buffer containing 0.05% sodium azide.

The CD4-coated magnetic beads were mixed with 500 μL of a nanobody mixture (0.1 μM anti-CD4-FLAG-Barcode 1 and 0.1 μM anti-GFP-FLAG-Barcode 2), and incubated for 2 h at room temperature. After removal of the supernatants, the beads were washed with 1 mL of TBS containing 0.05% Tween 20 twice and 1 mL of distilled water once. To cleave the peptide barcodes, the beads were incubated with 20 μL of 50 ng/mL enterokinase (New England BioLabs, Ipswich, MA, USA) for 16 h at 25°C. The supernatants were desalted using MonoSpin C18 (GL Sciences Inc., Tokyo, Japan) and freeze-dried. The dried pellet was resolved with 20 μL of 50 mM TEAB, filtered by Ultrafree-MC-HV Centrifugal Filters Durapore PVDF 0.45 μm (Merck Millipore, Burlington, MA, USA), and stored at −20°C until subjected to LC–MS/MS analysis.

### Quantification of peptide barcodes by LC¬MS/MS

Peptide barcodes cleaved by enterokinase were analyzed by LC (Nexera UHPLC/HPLC System; Shimadzu, Kyoto, Japan)¬triple quadrupole mass spectrometry (LCMS-8060; Shimadzu). Five microliters of the solution containing peptide barcodes was separated on a 50 mm InertSustainSwift^TM^ C18 column (P.N. 5020–88228, inner diameter 2.1 mm, particle size 1.9 μm; GL Science, Tokyo, Japan), which was kept at 40°C and injected into MS through a six-port injection/switching valve (Valco Instruments, Houston, TX, USA). Analysis of peptide barcodes was performed for 3.5 min with a flow rate of 600 μL/min. A gradient was produced by changing the mixing ratio of the two eluents: A, 0.1% (v/v) formic acid; and B, acetonitrile containing 0.1% (v/v) formic acid. The gradient was started with 5% B with holding for 0.5 min; this was then raised to 50% B for 2 min, then raised to 95% B with holding for 0.5 min, and finally the B concentration was immediately adjusted to 5% and held for 0.5 min to re-equilibrate the column. The autosampler was kept at 4°C and equipped with a black door. The interface temperature, the heat block temperature, and the desolvation line temperature were set at 300, 400, and 250°C, respectively. Nebulizer gas (N_2_), drying gas (N_2_), and heating gas (dried air) flow rates for making droplets were set at 2, 10, and 10 L/min, respectively.

For optimization of SRM methods, we analyzed all transitions of each synthetic peptide barcode made on Skyline [36], excluding sequences containing only FLAG sequences. Two sensitive transitions per peptide were selected and collision energy with the highest peak intensity was adopted (Table 2). Furthermore, these transitions were scheduled based on the obtained retention time. Ionized peptide barcodes were analyzed by the methods with a dwell time of 5 ms.

**Table 2:** Parameters for SRM analysis of peptide barcodes.

Barcode name	Peptide sequence	Precursor *m/z*	Precursor charge	CE	Product *m/z*	Product charge	Fragment ion
Barcode 1	WLFPVGDYKDDDDK	571.60	3	20.3	300.17	1	b2
					633.78	2	y11
Barcode 2	FVGARLDYKDDDDK	552.93	3	19.6	759.41	1	b7
					705.83	2	y12

## Supporting information

S1 Fig Plasmid sequences used in this study. (PDF)
