# Supplementary material for: Peptide barcoding for establishment of new types of genotype–phenotype linkages
Source: PLoS One. 2019 Apr 23;14(4):e0215993. doi: 10.1371/journal.pone.0215993 (PMC6478338; doi:10.1371/journal.pone.0215993)
Supplement: S1 Fig — (PDF) [file pone.0215993.s001.pdf]

Anti-CD4-FLAG (9681 bp)

BglII AleI

AGATCTAACATCCAAAGACGAAAGGTTGAATGAAACCTTTTTGCCATCCGACATCCACAGGTCCATTCTCACACATAAGTGCCAAACGCAACAGGAGGGGATACACT
TCTAGATTGTAGGTTTCTGCTTTCCAACTTACTTTGGAAAAACGGTAGGCTGTAGGTGTCCAGGTAAGAGTGTGTATTCACGGTTTGCGTTGTCCTCCCCTATGTGA

AOX1 promoter

20 40 60 80 100

SacI
BsaXI PpuMI Eco53kI

AGCAGCAGACCGTTGCAAACGCAGGACCTCCACTCCTCTTCTCCTCAACACCCACTTTTGCCATCGAAAAACCAGCCCAGTTATTGGGCTTGATTGGAGCTCGCTCA
TCGTCGTCTGGCAACGTTTGCGTCCTGGAGGTGAGGAGAAGAGGAGTTGTGGGTGAAAACGGTAGCTTTTTGGTCGGGTCAATAACCCGAACTAACCTCGAGCGAGT

AOX1 promoter

120 140 160 180 200

TTCCAATTCCTTCTATTAGGCTACTAACACCATGACTTTATTAGCCTGTCTATCCTGGCCCCCCTGGCGAGGTTCATGTTTGTTTATTTCCGAATGCAACAAGCTCC
AAGGTTAAGGAAGATAATCCGATGATTGTGGTACTGAAATAATCGGACAGATAGGACCGGGGGGACCGCTCCAAGTACAAACAAATAAAGGCTTACGTTGTTCGAGG

AOX1 promoter

220 240 260 280 300 320

PmeI

GCATTACACCCGAACATCACTCCAGATGAGGGCTTTCTGAGTGTGGGGTCAAATAGTTTCATGTTCCCCAAATGGCCCAAAACTGACAGTTTAAACGCTGTCTTGGA
CGTAATGTGGGCTTGTAGTGAGGTCTACTCCCGAAAGACTCACACCCCAGTTTATCAAAGTACAAGGGGTTTACCGGGTTTTGACTGTCAAATTTGCGACAGAACCT

AOX1 promoter

340 360 380 400 420

ACCTAATATGACAAAAGCGTGATCTCATCCAAGATGAACTAAGTTTGGTTCGTTGAAATGCTAACGGCCAGTTGGTCAAAAAGAAACTTCCAAAAGTCGCCATACCG
TGGATTATACTGTTTTCGCACTAGAGTAGGTTCTACTTGATTCAAACCAAGCAACTTTACGATTGCCGGTCAACCAGTTTTTCTTTGAAGGTTTTCAGCGGTATGGC

AOX1 promoter

440 460 480 500 520

BlpI

TTTGTCTTGTTTGGTATTGATTGACGAATGCTCAAAAATAATCTCATTAATGCTTAGCGCAGTCTCTCTATCGCTTCTGAACCCCGGTGCACCTGTGCCGAAACGCA
AAACAGAACAAACCATAACTAACTGCTTACGAGTTTTTATTAGAGTAATTACGAATCGCGTCAGAGAGATAGCGAAGACTTGGGGCCACGTGGACACGGCTTTGCGT

AOX1 promoter

540 560 580 600 620 640

XcmI

AATGGGGAAACACCCGCTTTTTGGATGATTATGCATTGTCTCCACATTGTATGCTTCCAAGATTCTGGTGGGAATACTGCTGATAGCCTAACGTTCATGATCAAAAT
TTACCCCTTTGTGGGCGAAAAACCTACTAATACGTAACAGAGGTGTAACATACGAAGGTTCTAAGACCACCCTTATGACGACTATCGGATTGCAAGTACTAGTTTTA

AOX1 promoter

660 680 700 720 740

TTAACTGTTCTAACCCCTACTTGACAGCAATATATAAACAGAAGGAAGCTGCCCTGTCTTAAACCTTTTTTTTTATCATCATTATTAGCTTACTTTCATAATTGCGA
AATTGACAAGATTGGGGATGAACTGTCGTTATATATTTGTCTTCCTTCGACGGGACAGAATTTGGAAAAAAAAATAGTAGTAATAATCGAATGAAAGTATTAACGCT

AOX1 promoter

760 780 800 820 840

BamHI

CTGGTTCCAATTGACAAGCTTTTGATTTTAACGACTTTTAACGACAACTTGAGAAGATCAAAAAACAACTAATTATTCGAAGGATCCAAACGATGAGATTTCCTTCA
GACCAAGGTTAACTGTTCGAAAACTAAAATTGCTGAAAATTGCTGTTGAACTCTTCTAGTTTTTTGTTGATTAATAAGCTTCCTAGGTTTGCTACTCTAAAGGAAGT

2 4
M R F P S

AOX1 promoter

860 880 900 920 940 960

ATTTTTACTGCAGTTTTATTCGCAGCATCCTCCGCATTAGCTGCTCCAGTCAACACTACAACAGAAGATGAAACGGCACAAATTCCGGCTGAAGCTGTCATCGGTTA
TAAAAATGACGTCAAAATAAGCGTCGTAGGAGGCGTAATCGACGAGGTCAGTTGTGATGTTGTCTTCTACTTTGCCGTGTTTAAGGCCGACTTCGACAGTAGCCAAT

6 8 10 12 14 16 18 20 22 24 26 28 30 32 34 36 38 40
I F T A V L F A A S S A L A A P V N T T T E D E T A Q I P A E A V I G Y

α-factor secretion signal

980 1,000 1,020 1,040 1,060

PsiI

CTCAGATTTAGAAGGGGATTTCGATGTTGCTGTTTTGCCATTTTCCAACAGCACAAATAACGGGTTATTGTTTATAAATACTACTATTGCCAGCATTGCTGCTAAAG
GAGTCTAAATCTTCCCCTAAAGCTACAACGACAAAACGGTAAAAGGTTGTCGTGTTTATTGCCCAATAACAAATATTTATGATGATAACGGTCGTAACGACGATTTC

42 44 46 48 50 52 54 56 58 60 62 64 66 68 70 72 74 76
S D L E G D F D V A V L P F S N S T N N G L L F I N T T I A S I A A K

α-factor secretion signal

1,080 1,100 1,120 1,140 1,160

PaeR7I
XhoI
TliI
EcoRI
SnaBI
BsaAI
SexAI

AAGAAGGGGTATCTCTCGAGAAAAGAGAGGCTGAAGCTTACGTAGAATTCGAAGTTCAGTTAGTTGAATCTGGTGGTGGCTCTGTCCAACCTGGTGGTTCTCTTACT
TTCTTCCCCATAGAGAGCTCTTTTCTCTCCGACTTCGAATGCATCTTAAGCTTCAAGTCAATCAACTTAGACCACCACCGAGACAGGTTGGACCACCAAGAGAATGA

78 80 82 84 86 88 90 92 94 96 98 100 102 104 106 108 110 112
E E G V S L E K R E A E A Y V E F E V Q L V E S G G G S V Q P G G S L T

α-factor secretion signal
CD4 nanobody

1,180 1,200 1,220 1,240 1,260 1,280

TTGTCCTGTGGTACTTCCGGTAGAACCTTCAACGTTATGGGATGGTTCAGACAAGCTCCTGGAAAGGAACGTGAGTTTGTCGCTGCTGTTAGGTGGTCTTCTACTGG
AACAGGACACCATGAAGGCCATCTTGGAAGTTGCAATACCCTACCAAGTCTGTTCGAGGACCTTTCCTTGCACTCAAACAGCGACGACAATCCACCAGAAGATGACC

114 116 118 120 122 124 126 128 130 132 134 136 138 140 142 144 146 148
L S C G T S G R T F N V M G W F R Q A P G K E R E F V A A V R W S S T G

CD4 nanobody

1,300 1,320 1,340 1,360 1,380

PsrI BstZ17I EcoRI

TATCTATTATACCCAATATGCTGACTCAGTTAAAAGTAGATTTACTATTTCAAGAGACAATGCAAAGAACACCGTATACTTGGAGATGAATTCACTGAAGCCAGAGG
ATAGATAATATGGGTTATACGACTGAGTCAATTTTCATCTAAATGATAAAGTTCTCTGTTACGTTTCTTGTGGCATATGAACCTCTACTTAAGTGACTTCGGTCTCC

150 152 154 156 158 160 162 164 166 168 170 172 174 176 178 180 182

I Y Y T Q Y A D S V K S R F T I S R D N A K N T V Y L E M N S L K P E

CD4 nanobody

1,400 1,420 1,440 1,460 1,480

Tth111I
PflFI

ACACGGCTGTCTACTACTGCGCAGCCGACACATATAACTCCAATCCAGCCAGATGGGACGGATATGACTTTCGAGGTCAAGGAACACAGGTGACAGTGTCCAGTGGA
TGTGCCGACAGATGATGACGCGTCGGCTGTGTATATTGAGGTTAGGTCGGTCTACCCTGCCTATACTGAAAGCTCCAGTTCCTTGTGTCCACTGTCACAGGTCACCT

184 186 188 190 192 194 196 198 200 202 204 206 208 210 212 214 216 218

D T A V Y Y C A A D T Y N S N P A R W D G Y D F R G Q G T Q V T V S S G

CD4 nanobody

1,500 1,520 1,540 1,560 1,580 1,600

NotI
EagI
AgeI

GGCAGTGACTACAAAGATGACGACGATAAATAAGCGGCCGCGAATTAATTCGCCTTAGACATGACTGTTCCTCAGTTCAAGTTGGGCACTTACGAGAAGACCGGTCT
CCGTCACTGATGTTTCTACTGCTGCTATTTATTCGCCGGCGCTTAATTAAGCGGAATCTGTACTGACAAGGAGTCAAGTTCAACCCGTGAATGCTCTTCTGGCCAGA

220 222 224 226 228 230

G S D Y K D D D D K *

FLAG

1,620 1,640 1,660 1,680 1,700

TGCTAGATTCTAATCAAGAGGATGTCAGAATGCCATTTGCCTGAGAGATGCAGGCTTCATTTTTGATACTTTTTTATTTGTAACCTATATAGTATAGGATTTTTTTT
ACGATCTAAGATTAGTTCTCCTACAGTCTTACGGTAAACGGACTCTCTACGTCCGAAGTAAAAACTATGAAAAAATAAACATTGGATATATCATATCCTAAAAAAAA

AOX1 terminator

1,720 1,740 1,760 1,780 1,800

BsiWI PvuII

GTCATTTTGTTTCTTCTCGTACGAGCTTGCTCCTGATCAGCCTATCTCGCAGCTGATGAATATCTTGTGGTAGGGGTTTGGGAAAATCATTCGAGTTTGATGTTTTT
CAGTAAAACAAAGAAGAGCATGCTCGAACGAGGACTAGTCGGATAGAGCGTCGACTACTTATAGAACACCATCCCCAAACCCTTTTAGTAAGCTCAAACTACAAAAA

AOX1 terminator

1,820 1,840 1,860 1,880 1,900 1,920

BcgI

CTTGGTATTTCCCACTCCTCTTCAGAGTACAGAAGATTAAGTGAGAAGTTCGTTTGTGCAAGCTTATCGATAAGCTTTAATGCGGTAGTTTATCACAGTTAAATTGC
GAACCATAAAGGGTGAGGAGAAGTCTCATGTCTTCTAATTCACTCTTCAAGCAAACACGTTCGAATAGCTATTCGAAATTACGCCATCAAATAGTGTCAATTTAACG

AOX1 terminator

1,940 1,960 1,980 2,000 2,020

```
TAACGCAGTCAGGCACCGTGTATGAAATCTAACAATGCGCTCATCGTCATCCTCGGCACCGTCACCCTGGATGCTGTAGGCATAGGCTTGGTTATGCCGGTACTGCC
ATTGCGTCAGTCCGTGGCACATACTTTAGATTGTTACGCGAGTAGCAGTAGGAGCCGTGGCAGTGGGACCTACGACATCCGTATCCGAACCAATACGGCCATGACGG
    2,040    2,060    2,080    2,100    2,120    2,140

AfeI
BtgZI    BmtI
EcoRV    NheI    FspAI
GGGCCTCTTGCGGGATATCGTCCATTCCGACAGCATCGCCAGTCACTATGGCGTGCTGCTAGCGCTATATGCGTTGATGCAATTTCTATGCGCACCCGTTCTCGGAG
CCCGGAGAACGCCCTATAGCAGGTAAGGCTGTCGTAGCGGTCAGTGATACCGCACGACGATCGCGATATACGCAACTACGTTAAAGATACGCGTGGGCAAGAGCCTC
    2,160    2,180    2,200    2,220    2,240

CACTGTCCGACCGCTTTGGCCGCCGCCCAGTCCTGCTCGCTTCGCTACTTGGAGCCACTATCGACTACGCGATCATGGCGACCACACCCGTCCTGTGGATCTATCGA
GTGACAGGCTGGCGAAACCGGCGGCGGGTCAGGACGAGCGAAGCGATGAACCTCGGTGATAGCTGATGCGCTAGTACCGCTGGTGTGGGCAGGACACCTAGATAGCT
    2,260    2,280    2,300    2,320    2,340

XbaI
ATCTAAATGTAAGTTAAAATCTCTAAATAATTAAATAAGTCCCAGTTTCTCCATACGAACCTTAACAGCATTGCGGTGAGCATCTAGACCTTCAACAGCAGCCAGAT
TAGATTTACATTCAATTTTAGAGATTTATTAATTTATTCAGGGTCAAAGAGGTATGCTTGGAATTGTCGTAACGCCACTCGTAGATCTGGAAGTTGTCGTCGGTCTA
    2,360    2,380    2,400    2,420    2,440    2,460

MscI    Bsu36I    HpaI
CCATCACTGCTTGGCCAATATGTTTCAGTCCCTCAGGAGTTACGTCTTGTGAAGTGATGAACTTCTGGAAGGTTGCAGTGTTAACTCCGCTGTATTGACGGGCATAT
GGTAGTGACGAACCGGTTATACAAAGTCAGGGAGTCCTCAATGCAGAACACTTCACTACTTGAAGACCTTCCAACGTCACAATTGAGGCGACATAACTGCCCGTATA
    2,480    2,500    2,520    2,540    2,560

KpnI
BsiWI    Acc65I
CCGTACGTTGGCAAAGTGTGGTTGGTACCGGAGGAGTAATCTCCACAACTCTCTGGAGAGTAGGCACCAACAAACACAGATCCAGCGTGTTGTACTTGATCAACATA
GGCATGCAACCGTTTCACACCAACCATGGCCTCCTCATTAGAGGTGTTGAGAGACCTCTCATCCGTGGTTGTTTGTGTCTAGGTCGCACAACATGAACTAGTTGTAT
    2,580    2,600    2,620    2,640    2,660

AloI
AGAAGAAGCATTCTCGATTTGCAGGATCAAGTGTTCAGGAGCGTACTGATTGGACATTTCCAAAGCCTGCTCGTAGGTTGCAACCGATAGGGTTGTAGAGTGTGCAA
TCTTCTTCGTAAGAGCTAAACGTCCTAGTTCACAAGTCCTCGCATGACTAACCTGTAAAGGTTTCGGACGAGCATCCAACGTTGGCTATCCCAACATCTCACACGTT
    2,680    2,700    2,720    2,740    2,760    2,780

BsgI
TACACTTGCGTACAATTTCAACCCTTGGCAACTGCACAGCTTGGTTGTGAACAGCATCTTCAATTCTGGCAAGCTCCTTGTCTGTCATATCGACAGCCAACAGAATC
ATGTGAACGCATGTTAAAGTTGGGAACCGTTGACGTGTCGAACCAACACTTGTCGTAGAAGTTAAGACCGTTCGAGGAACAGACAGTATAGCTGTCGGTTGTCTTAG
    2,800    2,820    2,840    2,860    2,880

ACCTGGGAATCAATACCATGTTCAGCTTGAGACAGAAGGTCTGAGGCAACGAAATCTGGATCAGCGTATTTATCAGCAATAACTAGAACTTCAGAAGGCCCAGCAGG
TGGACCCTTAGTTATGGTACAAGTCGAACTCTGTCTTCCAGACTCCGTTGCTTTAGACCTAGTCGCATAAATAGTCGTTATTGATCTTGAAGTCTTCCGGGTCGTCC
    2,900    2,920    2,940    2,960    2,980
```

CATGTCAATACTACACAGGGCTGATGTGTCATTTTGAACCATCATCTTGGCAGCAGTAACGAACTGGTTTCCTGGACCAAATATTTTGTCACACTTAGGAACAGTTT
GTACAGTTATGATGTGTCCCGACTACACAGTAAAACTTGGTAGTAGAACCGTCGTCATTGCTTGACCAAAGGACCTGGTTTATAAAACAGTGTGAATCCTTGTCAAA

3,000 3,020 3,040 3,060 3,080 3,100

BmtI NheI KpnI Acc65I

CTGTTCCGTAAGCCATAGCAGCTACTGCCTGGGCGCCTCCTGCTAGCACGATACACTTAGCACCAACCTTGTGGGCAACGTAGATGACTTCTGGGGTAAGGGTACCA
GACAAGGCATTCGGTATCGTCGATGACGGACCCGCGGAGGACGATCGTGCTATGTGAATCGTGGTTGGAACACCCGTTGCATCTACTGAAGACCCCATTCCCATGGT

3,120 3,140 3,160 3,180 3,200

TCCTTCTTAGGTGGAGATGCAAAAACAATTTCTTTGCAACCAGCAACTTTGGCAGGAACACCCAGCATCAGGGAAGTGGAAGGCAGAATTGCGGTTCCACCAGGAAT
AGGAAGAATCCACCTCTACGTTTTTGTTAAAGAAACGTTGGTCGTTGAAACCGTCCTTGTGGGTCGTAGTCCCTTCACCTTCCGTCTTAACGCCAAGGTGGTCCTTA

3,220 3,240 3,260 3,280 3,300

ATAGAGGCCAACTTTCTCAATAGGTCTTGCAAAACGAGAGCAGACTACACCAGGGCAAGTCTCAACTTGCAACGTCTCCGTTAGTTGAGCTTCATGGAATTTCCTGA
TATCTCCGGTTGAAAGAGTTATCCAGAACGTTTTGCTCTCGTCTGATGTGGTCCCGTTCAGAGTTGAACGTTGCAGAGGCAATCAACTCGAAGTACCTTAAAGGACT

3,320 3,340 3,360 3,380 3,400 3,420

CGTTATCTATAGAGAGATCAATGGCTCTCTTAACGTTATCTGGCAATTGCATAAGTTCCTCTGGGAAAGGAGCTTCTAACACAGGTGTCTTCAAAGCGACTCCATCA
GCAATAGATATCTCTCTAGTTACCGAGAGAATTGCAATAGACCGTTAACGTATTCAAGGAGACCCTTTCCTCGAAGATTGTGTCCACAGAAGTTTCGCTGAGGTAGT

3,440 3,460 3,480 3,500 3,520

SalI

AACTTGGCAGTTAGTTCTAAAAGGGCTTTGTCACCATTTTGACGAACATTGTCGACAATTGGTTTGACTAATTCCATAATCTGTTCCGTTTTCTGGATAGGACGACG
TTGAACCGTCAATCAAGATTTTCCCGAAACAGTGGTAAAACTGCTTGTAACAGCTGTTAACCAAACTGATTAAGGTATTAGACAAGGCAAAAGACCTATCCTGCTGC

3,540 3,560 3,580 3,600 3,620

StuI

AAGGGCATCTTCAATTTCTTGTGAGGAGGCCTTAGAAACGTCAATTTTGCACAATTCAATACGACCTTCAGAAGGGACTTCTTTAGGTTTGGATTCTTCTTTAGGTT
TTCCCGTAGAAGTTAAAGAACACTCCTCCGGAATCTTTGCAGTTAAAACGTGTTAAGTTATGCTGGAAGTCTTCCCTGAAGAAATCCAAACCTAAGAAGAAATCCAA

3,640 3,660 3,680 3,700 3,720 3,740

GTTCCTTGGTGTATCCTGGCTTGGCATCTCCTTTCCTTCTAGTGACCTTTAGGGACTTCATATCCAGGTTTCTCTCCACCTCGTCCAACGTCACACCGTACTTGGCA
CAAGGAACCACATAGGACCGAACCGTAGAGGAAAGGAAGATCACTGGAAATCCCTGAAGTATAGGTCCAAAGAGAGGTGGAGCAGGTTGCAGTGTGGCATGAACCGT

3,760 3,780 3,800 3,820 3,840

CATCTAACTAATGCAAAATAAAATAAGTCAGCACATTCCCAGGCTATATCTTCCTTGGATTTAGCTTCTGCAAGTTCATCAGCTTCCTCCCTAATTTTAGCGTTCAA
GTAGATTGATTACGTTTTATTTTATTCAGTCGTGTAAGGGTCCGATATAGAAGGAACCTAAATCGAAGACGTTCAAGTAGTCGAAGGAGGGATTAAAATCGCAAGTT

3,860 3,880 3,900 3,920 3,940

DraIII NcoI MscI

CAAAACTTCGTCGTCAAATAACCGTTTGGTATAAGAACCTTCTGGAGCATTGCTCTTACGATCCCACAAGGTGGCTTCCATGGCTCTAAGACCCTTTGATTGGCCAA
GTTTTGAAGCAGCAGTTTATTGGCAAACCATATTCTTGGAAGACCTCGTAACGAGAATGCTAGGGTGTTCCACCGAAGGTACCGAGATTCTGGGAAACTAACCGGTT

3,960 3,980 4,000 4,020 4,040 4,060

AACAGGAAGTGCGTTCCAAGTGACAGAAACCAACACCTGTTTGTTCAACCACAAATTTCAAGCAGTCTCCATCACAATCCAATTCGATACCCAGCAACTTTTGAGTT
TTGTCCTTCACGCAAGGTTCACTGTCTTTGGTTGTGGACAAACAAGTTGGTGTTTAAAGTTCGTCAGAGGTAGTGTTAGGTTAAGCTATGGGTCGTTGAAAACTCAA

4,080 4,100 4,120 4,140 4,160

BspEI

GCTCCAGATGTAGCACCTTTATACCACAAACCGTGACGACGAGATTGGTAGACTCCAGTTTGTGTCCTTATAGCCTCCGGAATAGACTTTTTGGACGAGTACACCAG
CGAGGTCTACATCGTGGAAATATGGTGTTTGGCACTGCTGCTCTAACCATCTGAGGTCAAACACAGGAATATCGGAGGCCTTATCTGAAAAACCTGCTCATGTGGTC

4,180 4,200 4,220 4,240 4,260 4,280

GCCCAACGAGTAATTAGAAGAGTCAGCCACCAAAGTAGTGAATAGACCATCGGGGCGGTCAGTAGTCAAAGACGCCAACAAAATTTCACTGACAGGGAACTTTTTGA
CGGGTTGCTCATTAATCTTCTCAGTCGGTGGTTTCATCACTTATCTGGTAGCCCCGCCAGTCATCAGTTTCTGCGGTTGTTTTAAAGTGACTGTCCCTTGAAAAACT

4,300 4,320 4,340 4,360 4,380

CATCTTCAGAAAGTTCGTATTCAGTAGTCAATTGCCGAGCATCAATAATGGGGATTATACCAGAAGCAACAGTGGAAGTCACATCTACCAACTTTGCGGTCTCAGAA
GTAGAAGTCTTTCAAGCATAAGTCATCAGTTAACGGCTCGTAGTTATTACCCCTAATATGGTCTTCGTTGTCACCTTCAGTGTAGATGGTTGAAACGCCAGAGTCTT

4,400 4,420 4,440 4,460 4,480

AAAGCATAAACAGTTCTACTACCGCCATTAGTGAAACTTTTCAAATCGCCCAGTGGAGAAGAAAAAGGCACAGCGATACTAGCATTAGCGGGCAAGGATGCAACTTT
TTTCGTATTTGTCAAGATGATGGCGGTAATCACTTTGAAAAGTTTAGCGGGTCACCTCTTCTTTTTCCGTGTCGCTATGATCGTAATCGCCCGTTCCTACGTTGAAA

4,500 4,520 4,540 4,560 4,580 4,600

PpuMI BsrGI

ATCAACCAGGGTCCTATAGATAACCCTAGCGCCTGGGATCATCCTTTGGACAACTCTTTCTGCCAAATCTAGGTCCAAAATCACTTCATTGATACCATTATTGTACA
TAGTTGGTCCCAGGATATCTATTGGGATCGCGGACCCTAGTAGGAAACCTGTTGAGAAAGACGGTTTAGATCCAGGTTTTAGTGAAGTAACTATGGTAATAACATGT

4,620 4,640 4,660 4,680 4,700

ACTTGAGCAAGTTGTCGATCAGCTCCTCAAATTGGTCCTCTGTAACGGATGACTCAACTTGCACATTAACTTGAAGCTCAGTCGATTGAGTGAACTTGATCAGGTTG
TGAACTCGTTCAACAGCTAGTCGAGGAGTTTAACCAGGAGACATTGCCTACTGAGTTGAACGTGTAATTGAACTTCGAGTCAGCTAACTCACTTGAACTAGTCCAAC

4,720 4,740 4,760 4,780 4,800

PvuII BsgI

TGCAGCTGGTCAGCAGCATAGGGAAACACGGCTTTTCCTACCAAACTCAAGGAATTATCAAACTCTGCAACACTTGCGTATGCAGGTAGCAAGGGAAATGTCATACT
ACGTCGACCAGTCGTCGTATCCCTTTGTGCCGAAAAGGATGGTTTGAGTTCCTTAATAGTTTGAGACGTTGTGAACGCATACGTCCATCGTTCCCTTTACAGTATGA

4,820 4,840 4,860 4,880 4,900 4,920

TGAAGTCGGACAGTGAGTGTAGTCTTGAGAAATTCTGAAGCCGTATTTTTATTATCAGTGAGTCAGTCATCAGGAGATCCTCTACGCCGGACGCATCGTGGCCGACC
ACTTCAGCCTGTCACTCACATCAGAACTCTTTAAGACTTCGGCATAAAAATAATAGTCACTCAGTCAGTAGTCCTCTAGGAGATGCGGCCTGCGTAGCACCGGCTGG

4,940 4,960 4,980 5,000 5,020

Bpu10I
SbfI BbvCI StuI

TGCAGGGGGGGGGGGGGCGCTGAGGTCTGCCTCGTGAAGAAGGTGTTGCTGACTCATACCAGGCCTGAATCGCCCCATCATCCAGCCAGAAAGTGAGGGAGCCACGG
ACGTCCCCCCCCCCCCCGCGACTCCAGACGGAGCACTTCTTCCACAACGACTGAGTATGGTCCGGACTTAGCGGGGTAGTAGGTCGGTCTTTCACTCCCTCGGTGCC

5,040 5,060 5,080 5,100 5,120

BsaXI
TTGATGAGAGCTTTGTTGTAGGTGGACCAGTTGGTGATTTTGAACTTTTGCTTTGCCACGGAACGGTCTGCGTTGTCGGGAAGATGCGTGATCTGATCCTTCAACTC
AACTACTCTCGAAACAACATCCACCTGGTCAACCACTAAAACTTGAAAACGAAACGGTGCCTTGCCAGACGCAACAGCCCTTCTACGCACTAGACTAGGAAGTTGAG
5,140 5,160 5,180 5,200 5,220 5,240

AGCAAAAGTTCGATTTATTCAACAAAGCCGCCGTCCCGTCAAGTCAGCGTAATGCTCTGCCAGTGTTACAACCAATTAACCAATTCTGATTAGAAAAACTCATCGAG
TCGTTTTCAAGCTAAATAAGTTGTTTCGGCGGCAGGGCAGTTCAGTCGCATTACGAGACGGTCACAATGTTGGTTAATTGGTTAAGACTAATCTTTTTGAGTAGCTC
5,260 5,280 5,300 5,320 5,340

CATCAAATGAAACTGCAATTTATTCATATCAGGATTATCAATACCATATTTTTGAAAAAGCCGTTTCTGTAATGAAGGAGAAAACTCACCGAGGCAGTTCCATAGGA
GTAGTTTACTTTGACGTTAAATAAGTATAGTCCTAATAGTTATGGTATAAAAACTTTTTCGGCAAAGACATTACTTCCTCTTTTGAGTGGCTCCGTCAAGGTATCCT
5,360 5,380 5,400 5,420 5,440

TGGCAAGATCCTGGTATCGGTCTGCGATTCCGACTCGTCCAACATCAATACAACCTATTAATTTCCCCTCGTCAAAAATAAGGTTATCAAGTGAGAAATCACCATGA
ACCGTTCTAGGACCATAGCCAGACGCTAAGGCTGAGCAGGTTGTAGTTATGTTGGATAATTAAAGGGGAGCAGTTTTTATTCCAATAGTTCACTCTTTAGTGGTACT
5,460 5,480 5,500 5,520 5,540 5,560

GTGACGACTGAATCCGGTGAGAATGGCAAAAGCTTATGCATTTCTTTCCAGACTTGTTCAACAGGCCAGCCATTACGCTCGTCATCAAAATCACTCGCATCAACCAA
CACTGCTGACTTAGGCCACTCTTACCGTTTTCGAATACGTAAAGAAAGGTCTGAACAAGTTGTCCGGTCGGTAATGCGAGCAGTAGTTTTAGTGAGCGTAGTTGGTT
5,580 5,600 5,620 5,640 5,660

Bpu10I AsiSI
AjuI PvuI
ACCGTTATTCATTCGTGATTGCGCCTGAGCGAGACGAAATACGCGATCGCTGTTAAAAGGACAATTACAAACAGGAATCGAATGCAACCGGCGCAGGAACACTGCCA
TGGCAATAAGTAAGCACTAACGCGGACTCGCTCTGCTTTATGCGCTAGCGACAATTTTCCTGTTAATGTTTGTCCTTAGCTTACGTTGGCCGCGTCCTTGTGACGGT
5,680 5,700 5,720 5,740 5,760

SmaI
TspMI
XmaI
GCGCATCAACAATATTTTCACCTGAATCAGGATATTCTTCTAATACCTGGAATGCTGTTTTCCCGGGGATCGCAGTGGTGAGTAACCATGCATCATCAGGAGTACGG
CGCGTAGTTGTTATAAAAGTGGACTTAGTCCTATAAGAAGATTATGGACCTTACGACAAAAGGGCCCCTAGCGTCACCACTCATTGGTACGTAGTAGTCCTCATGCC
5,780 5,800 5,820 5,840 5,860 5,880

ATAAAATGCTTGATGGTCGGAAGAGGCATAAATTCCGTCAGCCAGTTTAGTCTGACCATCTCATCTGTAACATCATTGGCAACGCTACCTTTGCCATGTTTCAGAAA
TATTTTACGAACTACCAGCCTTCTCCGTATTTAAGGCAGTCGGTCAAATCAGACTGGTAGAGTAGACATTGTAGTAACCGTTGCGATGGAAACGGTACAAAGTCTTT
5,900 5,920 5,940 5,960 5,980

NruI
CAACTCTGGCGCATCGGGCTTCCCATACAATCGATAGATTGTCGCACCTGATTGCCCGACATTATCGCGAGCCCATTTATACCCATATAAATCAGCATCCATGTTGG
GTTGAGACCGCGTAGCCCGAAGGGTATGTTAGCTATCTAACAGCGTGGACTAACGGGCTGTAATAGCGCTCGGGTAAATATGGGTATATTTAGTCGTAGGTACAACC
6,000 6,020 6,040 6,060 6,080

PaeR7I
PspXI
XhoI
TliI

AATTTAATCGCGGCCTCGAGCAAGACGTTTCCCGTTGAATATGGCTCATAACACCCCTTGTATTACTGTTTATGTAAGCAGACAGTTTTATTGTTCATGATGATATA
TTAAATTAGCGCCGGAGCTCGTTCTGCAAAGGGCAACTTATACCGAGTATTGTGGGGAACATAATGACAAATACATTCGTCTGTCAAAATAACAAGTACTACTATAT

6,100 6,120 6,140 6,160 6,180 6,200

DraIII SbfI SgrAI

TTTTTATCTTGTGCAATGTAACATCAGAGATTTTGAGACACAACGTGGCTTTCCCCCCCCCCCCTGCAGGTCGGCATCACCGGCGCCACAGGTGCGGTTGCTGGCGC
AAAAATAGAACACGTTACATTGTAGTCTCTAAAACTCTGTGTTGCACCGAAAGGGGGGGGGGGGACGTCCAGCCGTAGTGGCCGCGGTGTCCACGCCAACGACCGCG

6,220 6,240 6,260 6,280 6,300

AfeI

CTATATCGCCGACATCACCGATGGGGAAGATCGGGCTCGCCACTTCGGGCTCATGAGCGCTTGTTTCGGCGTGGGTATGGTGGCAGGCCCCGTGGCCGGGGGACTGT
GATATAGCGGCTGTAGTGGCTACCCCTTCTAGCCCGAGCGGTGAAGCCCGAGTACTCGCGAACAAAGCCGCACCCATACCACCGTCCGGGGCACCGGCCCCCTGACA

6,320 6,340 6,360 6,380 6,400 6,420

SphI

TGGGCGCCATCTCCTTGCATGCACCATTCCTTGCGGCGGCGGTGCTCAACGGCCTCAACCTACTACTGGGCTGCTTCCTAATGCAGGAGTCGCATAAGGGAGAGCGT
ACCCGCGGTAGAGGAACGTACGTGGTAAGGAACGCCGCCGCCACGAGTTGCCGGAGTTGGATGATGACCCGACGAAGGATTACGTCCTCAGCGTATTCCCTCTCGCA

6,440 6,460 6,480 6,500 6,520

CGAGTATCTATGATTGGAAGTATGGGAATGGTGATACCCGCATTCTTCAGTGTCTTGAGGTCTCCTATCAGATTATGCCCAACTAAAGCAACCGGAGGAGGAGATTT
GCTCATAGATACTAACCTTCATACCCTTACCACTATGGGCGTAAGAAGTCACAGAACTCCAGAGGATAGTCTAATACGGGTTGATTTCGTTGGCCTCCTCCTCTAAA

6,540 6,560 6,580 6,600 6,620

CATGGTAAATTTCTCTGACTTTTGGTCATCAGTAGACTCGAACTGTGAGACTATCTCGGTTATGACAGCAGAAATGTCCTTCTTGGAGACAGTAAATGAAGTCCCAC
GTACCATTTAAAGAGACTGAAAACCAGTAGTCATCTGAGCTTGACACTCTGATAGAGCCAATACTGTCGTCTTTACAGGAAGAACCTCTGTCATTTACTTCAGGGTG

6,640 6,660 6,680 6,700 6,720 6,740

CAATAAAGAAATCCTTGTTATCAGGAACAAACTTCTTGTTTCGAACTTTTTCGGTGCCTTGAACTATAAAATGTAGAGTGGATATGTCGGGTAGGAATGGAGCGGGC
GTTATTTCTTTAGGAACAATAGTCCTTGTTTGAAGAACAAAGCTTGAAAAAGCCACGGAACTTGATATTTTACATCTCACCTATACAGCCCATCCTTACCTCGCCCG

6,760 6,780 6,800 6,820 6,840

AAATGCTTACCTTCTGGACCTTCAAGAGGTATGTAGGGTTTGTAGATACTGATGCCAACTTCAGTGACAACGTTGCTATTTCGTTCAAACCATTCCGAATCCAGAGA
TTTACGAATGGAAGACCTGGAAGTTCTCCATACATCCCAAACATCTATGACTACGGTTGAAGTCACTGTTGCAACGATAAAGCAAGTTTGGTAAGGCTTAGGTCTCT

6,860 6,880 6,900 6,920 6,940

AATCAAAGTTGTTTGTCTACTATTGATCCAAGCCAGTGCGGTCTTGAAACTGACAATAGTGTGCTCGTGTTTTGAGGTCATCTTTGTATGAATAAATCTAGTCTTTG
TTAGTTTCAACAAACAGATGATAACTAGGTTCGGTCACGCCAGAACTTTGACTGTTATCACACGAGCACAAAACTCCAGTAGAAACATACTTATTTAGATCAGAAAC

6,960 6,980 7,000 7,020 7,040 7,060

```
ATCTAAATAATCTTGACGAGCCAAGGCGATAAATACCCAAATCTAAAACTCTTTTAAAACGTTAAAAGGACAAGTATGTCTGCCTGTATTAAACCCCAAATCAGCTC
TAGATTTATTAGAACTGCTCGGTTCCGCTATTTATGGGTTTAGATTTTGAGAAAATTTTGCAATTTTCCTGTTCATACAGACGGACATAATTTGGGGTTTAGTCGAG
        7,080               7,100               7,120               7,140               7,160

                                                                      EcoRV
GTAGTCTGATCCTCATCAACTTGAGGGGCACTATCTTGTTTTAGAGAAATTTGCGGAGATGCGATATCGAGAAAAAGGTACGCTGATTTTAAACGTGAAATTTATCT
CATCAGACTAGGAGTAGTTGAACTCCCCGTGATAGAACAAAATCTCTTTAAACGCCTCTACGCTATAGCTCTTTTTCCATGCGACTAAAATTTGCACTTTAAATAGA
   7,180               7,200               7,220               7,240               7,260

  BglII
CAAGATCTCTGCCTCGCGCGTTTCGGTGATGACGGTGAAAACCTCTGACACATGCAGCTCCCGGAGACGGTCACAGCTTGTCTGTAAGCGGATGCCGGGAGCAGACA
GTTCTAGAGACGGAGCGCGCAAAGCCACTACTGCCACTTTTGGAGACTGTGTACGTCGAGGGCCTCTGCCAGTGTCGAACAGACATTCGCCTACGGCCCTCGTCTGT
7,280               7,300               7,320               7,340               7,360               7,380

                                                       Tth111I
                                                       PflFI BsaAI                    BstZ17I
AGCCCGTCAGGGCGCGTCAGCGGGTGTTGGCGGGTGTCGGGGCGCAGCCATGACCCAGTCACGTAGCGATAGCGGAGTGTATACTGGCTTAACTATGCGGCATCAGA
TCGGGCAGTCCCGCGCAGTCGCCCACAACCGCCCACAGCCCCGCGTCGGTACTGGGTCAGTGCATCGCTATCGCCTCACATATGACCGAATTGATACGCCGTAGTCT
          7,400               7,420               7,440               7,460               7,480

                                                                                        BspQI
                          NdeI                                                          SapI
GCAGATTGTACTGAGAGTGCACCATATGCGGTGTGAAATACCGCACAGATGCGTAAGGAGAAAATACCGCATCAGGCGCTCTTCCGCTTCCTCGCTCACTGACTCGC
CGTCTAACATGACTCTCACGTGGTATACGCCACACTTTATGGCGTGTCTACGCATTCCTCTTTTATGGCGTAGTCCGCGAGAAGGCGAAGGAGCGAGTGACTGAGCG
         7,500               7,520               7,540               7,560               7,580

                                                                                                 AflIII
                                                                                                 PciI
TGCGCTCGGTCGTTCGGCTGCGGCGAGCGGTATCAGCTCACTCAAAGGCGGTAATACGGTTATCCACAGAATCAGGGGATAACGCAGGAAAGAACATGTGAGCAAAA
ACGCGAGCCAGCAAGCCGACGCCGCTCGCCATAGTCGAGTGAGTTTCCGCCATTATGCCAATAGGTGTCTTAGTCCCCTATTGCGTCCTTTCTTGTACACTCGTTTT
7,600               7,620               7,640               7,660               7,680               7,700

GGCCAGCAAAAGGCCAGGAACCGTAAAAAGGCCGCGTTGCTGGCGTTTTTCCATAGGCTCCGCCCCCCTGACGAGCATCACAAAAATCGACGCTCAAGTCAGAGGTG
CCGGTCGTTTTCCGGTCCTTGGCATTTTTCCGGCGCAACGACCGCAAAAAGGTATCCGAGGCGGGGGGACTGCTCGTAGTGTTTTTAGCTGCGAGTTCAGTCTCCAC
                             <-------------------------- ColE1 origin --------------------------
               7,720               7,740               7,760               7,780               7,800

GCGAAACCCGACAGGACTATAAAGATACCAGGCGTTTCCCCCTGGAAGCTCCCTCGTGCGCTCTCCTGTTCCGACCCTGCCGCTTACCGGATACCTGTCCGCCTTTC
CGCTTTGGGCTGTCCTGATATTTCTATGGTCCGCAAAGGGGGACCTTCGAGGGAGCACGCGAGAGGACAAGGCTGGGACGGCGAATGGCCTATGGACAGGCGGAAAG
------------------------------------------------ ColE1 origin ------------------------------------------------
       7,820               7,840               7,860               7,880               7,900
```

TCCCTTCGGGAAGCGTGGCGCTTTCTCAATGCTCACGCTGTAGGTATCTCAGTTCGGTGTAGGTCGTTCGCTCCAAGCTGGGCTGTGTGCACGAACCCCCCGTTCAG
AGGGAAGCCCTTCGCACCGCGAAAGAGTTACGAGTGCGACATCCATAGAGTCAAGCCACATCCAGCAAGCGAGGTTCGACCCGACACACGTGCTTGGGGGGCAAGTC

ColE1 origin

7,920 7,940 7,960 7,980 8,000 8,020

CCCGACCGCTGCGCCTTATCCGGTAACTATCGTCTTGAGTCCAACCCGGTAAGACACGACTTATCGCCACTGGCAGCAGCCACTGGTAACAGGATTAGCAGAGCGAG
GGGCTGGCGACGCGGAATAGGCCATTGATAGCAGAACTCAGGTTGGGCCATTCTGTGCTGAATAGCGGTGACCGTCGTCGGTGACCATTGTCCTAATCGTCTCGCTC

ColE1 origin

8,040 8,060 8,080 8,100 8,120

GTATGTAGGCGGTGCTACAGAGTTCTTGAAGTGGTGGCCTAACTACGGCTACACTAGAAGGACAGTATTTGGTATCTGCGCTCTGCTGAAGCCAGTTACCTTCGGAA
CATACATCCGCCACGATGTCTCAAGAACTTCACCACCGGATTGATGCCGATGTGATCTTCCTGTCATAAACCATAGACGCGAGACGACTTCGGTCAATGGAAGCCTT

ColE1 origin

8,140 8,160 8,180 8,200 8,220

AAAGAGTTGGTAGCTCTTGATCCGGCAAACAAACCACCGCTGGTAGCGGTGGTTTTTTTGTTTGCAAGCAGCAGATTACGCGCAGAAAAAAAGGATCTCAAGAAGAT
TTTCTCAACCATCGAGAACTAGGCCGTTTGTTTGGTGGCGACCATCGCCACCAAAAAAACAAACGTTCGTCGTCTAATGCGCGTCTTTTTTTCCTAGAGTTCTTCTA

ColE1 origin

8,240 8,260 8,280 8,300 8,320 8,340

CCTTTGATCTTTTCTACGGGGTCTGACGCTCAGTGGAACGAAAACTCACGTTAAGGGATTTTGGTCATGAGATTATCAAAAAGGATCTTCACCTAGATCCTTTTAAA
GGAAACTAGAAAAGATGCCCCAGACTGCGAGTCACCTTGCTTTTGAGTGCAATTCCCTAAAACCAGTACTCTAATAGTTTTTCCTAGAAGTGGATCTAGGAAAATTT

ColE1 origin

8,360 8,380 8,400 8,420 8,440

TTAAAAATGAAGTTTTAAATCAATCTAAAGTATATATGAGTAAACTTGGTCTGACAGTTACCAATGCTTAATCAGTGAGGCACCTATCTCAGCGATCTGTCTATTTC
AATTTTTACTTCAAAATTTAGTTAGATTTCATATATACTCATTTGAACCAGACTGTCAATGGTTACGAATTAGTCACTCCGTGGATAGAGTCGCTAGACAGATAAAG

AmpR

8,460 8,480 8,500 8,520 8,540 8,560

GTTCATCCATAGTTGCCTGACTCCCCGTCGTGTAGATAACTACGATACGGGAGGGCTTACCATCTGGCCCCAGTGCTGCAATGATACCGCGAGACCCACGCTCACCG
CAAGTAGGTATCAACGGACTGAGGGGCAGCACATCTATTGATGCTATGCCCTCCCGAATGGTAGACCGGGGTCACGACGTTACTATGGCGCTCTGGGTGCGAGTGGC

AmpR

8,580 8,600 8,620 8,640 8,660

GCTCCAGATTTATCAGCAATAAACCAGCCAGCCGGAAGGGCCGAGCGCAGAAGTGGTCCTGCAACTTTATCCGCCTCCATCCAGTCTATTAATTGTTGCCGGGAAGC
CGAGGTCTAAATAGTCGTTATTTGGTCGGTCGGCCTTCCCGGCTCGCGTCTTCACCAGGACGTTGAAATAGGCGGAGGTAGGTCAGATAATTAACAACGGCCCTTCG

AmpR

8,680 8,700 8,720 8,740 8,760

TAGAGTAAGTAGTTCGCCAGTTAATAGTTTGCGCAACGTTGTTGCCATTGCTGCAGGCATCGTGGTGTCACGCTCGTCGTTTGGTATGGCTTCATTCAGCTCCGGTT
ATCTCATTCATCAAGCGGTCAATTATCAAACGCGTTGCAACAACGGTAACGACGTCCGTAGCACCACAGTGCGAGCAGCAAACCATACCGAAGTAAGTCGAGGCCAA
AmpR
8,780 8,800 8,820 8,840 8,860 8,880

PvuI
CCCAACGATCAAGGCGAGTTACATGATCCCCCATGTTGTGCAAAAAAGCGGTTAGCTCCTTCGGTCCTCCGATCGTTGTCAGAAGTAAGTTGGCCGCAGTGTTATCA
GGGTTGCTAGTTCCGCTCAATGTACTAGGGGGTACAACACGTTTTTTCGCCAATCGAGGAAGCCAGGAGGCTAGCAACAGTCTTCATTCAACCGGCGTCACAATAGT
AmpR
8,900 8,920 8,940 8,960 8,980

ScaI BcgI
CTCATGGTTATGGCAGCACTGCATAATTCTCTTACTGTCATGCCATCCGTAAGATGCTTTTCTGTGACTGGTGAGTACTCAACCAAGTCATTCTGAGAATAGTGTAT
GAGTACCAATACCGTCGTGACGTATTAAGAGAATGACAGTACGGTAGGCATTCTACGAAAAGACACTGACCACTCATGAGTTGGTTCAGTAAGACTCTTATCACATA
AmpR
9,000 9,020 9,040 9,060 9,080

GCGGCGACCGAGTTGCTCTTGCCCGGCGTCAACACGGGATAATACCGCGCCACATAGCAGAACTTTAAAAGTGCTCATCATTGGAAAACGTTCTTCGGGGCGAAAAC
CGCCGCTGGCTCAACGAGAACGGGCCGCAGTTGTGCCCTATTATGGCGCGGTGTATCGTCTTGAAATTTTCACGAGTAGTAACCTTTTGCAAGAAGCCCCGCTTTTG
AmpR
9,100 9,120 9,140 9,160 9,180 9,200

TCTCAAGGATCTTACCGCTGTTGAGATCCAGTTCGATGTAACCCACTCGTGCACCCAACTGATCTTCAGCATCTTTTACTTTCACCAGCGTTTCTGGGTGAGCAAAA
AGAGTTCCTAGAATGGCGACAACTCTAGGTCAAGCTACATTGGGTGAGCACGTGGGTTGACTAGAAGTCGTAGAAAATGAAAGTGGTCGCAAAGACCCACTCGTTTT
9,220 9,240 9,260 9,280 9,300

ACAGGAAGGCAAAATGCCGCAAAAAAGGGAATAAGGGCGACACGGAAATGTTGAATACTCATACTCTTCCTTTTTCAATATTATTGAAGCATTTATCAGGGTTATTG
TGTCCTTCCGTTTTACGGCGTTTTTTCCCTTATTCCCGCTGTGCCTTTACAACTTATGAGTATGAGAAGGAAAAAGTTATAATAACTTCGTAAATAGTCCCAATAAC
9,320 9,340 9,360 9,380 9,400

AatII
ZraI
TCTCATGAGCGGATACATATTTGAATGTATTTAGAAAAATAAACAAATAGGGGTTCCGCGCACATTTCCCCGAAAAGTGCCACCTGACGTCTAAGAAACCATTATTA
AGAGTACTCGCCTATGTATAAACTTACATAAATCTTTTTATTTGTTTATCCCCAAGGCGCGTGTAAAGGGGCTTTTCACGGTGGACTGCAGATTCTTTGGTAATAAT
9,420 9,440 9,460 9,480 9,500 9,520

TCATGACATTAACCTATAAAAATAGGCGTATCACGAGGCCCTTTCGTCTTCAAGAATTAATTCTCATGTTTGACAGCTTATCATCGATAAGCTGACTCATGTTGGTA
AGTACTGTAATTGGATATTTTTATCCGCATAGTGCTCCGGGAAAGCAGAAGTTCTTAATTAAGAGTACAAACTGTCGAATAGTAGCTATTCGACTGAGTACAACCAT
9,540 9,560 9,580 9,600 9,620

TTGTGAAATAGACGCAGATCGGGAACACTGAAAAATAACAGTTATTATTCG
AACACTTTATCTGCGTCTAGCCCTTGTGACTTTTTATTGTCAATAATAAGC
9,640 9,650 9,660 9,670 9,680

Anti-CD4-FLAG-Barcode1 (9723 bp)

BglII AleI

AGATCTAACATCCAAAGACGAAAGGTTGAATGAAACCTTTTTGCCATCCGACATCCACAGGTCCATTCTCACACATAAGTGCCAAACGCAACAGGAGGGGATACACT
TCTAGATTGTAGGTTTCTGCTTTCCAACTTACTTTGGAAAAACGGTAGGCTGTAGGTGTCCAGGTAAGAGTGTGTATTCACGGTTTGCGTTGTCCTCCCCTATGTGA

AOX1 promoter

20 40 60 80 100

SacI
BsaXI PpuMI Eco53kI

AGCAGCAGACCGTTGCAAACGCAGGACCTCCACTCCTCTTCTCCTCAACACCCACTTTTGCCATCGAAAAACCAGCCCAGTTATTGGGCTTGATTGGAGCTCGCTCA
TCGTCGTCTGGCAACGTTTGCGTCCTGGAGGTGAGGAGAAGAGGAGTTGTGGGTGAAAACGGTAGCTTTTTGGTCGGGTCAATAACCCGAACTAACCTCGAGCGAGT

AOX1 promoter

120 140 160 180 200

TTCCAATTCCTTCTATTAGGCTACTAACACCATGACTTTATTAGCCTGTCTATCCTGGCCCCCCTGGCGAGGTTCATGTTTGTTTATTTCCGAATGCAACAAGCTCC
AAGGTTAAGGAAGATAATCCGATGATTGTGGTACTGAAATAATCGGACAGATAGGACCGGGGGGACCGCTCCAAGTACAAACAAATAAAGGCTTACGTTGTTCGAGG

AOX1 promoter

220 240 260 280 300 320

PmeI

GCATTACACCCGAACATCACTCCAGATGAGGGCTTTCTGAGTGTGGGGTCAAATAGTTTCATGTTCCCCAAATGGCCCAAAACTGACAGTTTAAACGCTGTCTTGGA
CGTAATGTGGGCTTGTAGTGAGGTCTACTCCCGAAAGACTCACACCCCAGTTTATCAAAGTACAAGGGGTTTACCGGGTTTTGACTGTCAAATTTGCGACAGAACCT

AOX1 promoter

340 360 380 400 420

ACCTAATATGACAAAAGCGTGATCTCATCCAAGATGAACTAAGTTTGGTTCGTTGAAATGCTAACGGCCAGTTGGTCAAAAAGAAACTTCCAAAAGTCGCCATACCG
TGGATTATACTGTTTTCGCACTAGAGTAGGTTCTACTTGATTCAAACCAAGCAACTTTACGATTGCCGGTCAACCAGTTTTTCTTTGAAGGTTTTCAGCGGTATGGC

AOX1 promoter

440 460 480 500 520

BlpI

TTTGTCTTGTTTGGTATTGATTGACGAATGCTCAAAAATAATCTCATTAATGCTTAGCGCAGTCTCTCTATCGCTTCTGAACCCCGGTGCACCTGTGCCGAAACGCA
AAACAGAACAAACCATAACTAACTGCTTACGAGTTTTTATTAGAGTAATTACGAATCGCGTCAGAGAGATAGCGAAGACTTGGGGCCACGTGGACACGGCTTTGCGT

AOX1 promoter

540 560 580 600 620 640

XcmI

AATGGGGAAACACCCGCTTTTTGGATGATTATGCATTGTCTCCACATTGTATGCTTCCAAGATTCTGGTGGGAATACTGCTGATAGCCTAACGTTCATGATCAAAAT
TTACCCCTTTGTGGGCGAAAAACCTACTAATACGTAACAGAGGTGTAACATACGAAGGTTCTAAGACCACCCTTATGACGACTATCGGATTGCAAGTACTAGTTTTA

AOX1 promoter

660 680 700 720 740

TTAACTGTTCTAACCCCTACTTGACAGCAATATATAAACAGAAGGAAGCTGCCCTGTCTTAAACCTTTTTTTTTATCATCATTATTAGCTTACTTTCATAATTGCGA
AATTGACAAGATTGGGGATGAACTGTCGTTATATATTTGTCTTCCTTCGACGGGACAGAATTTGGAAAAAAAAATAGTAGTAATAATCGAATGAAAGTATTAACGCT

AOX1 promoter

760 780 800 820 840

BamHI

CTGGTTCCAATTGACAAGCTTTTGATTTTAACGACTTTTAACGACAACTTGAGAAGATCAAAAAACAACTAATTATTCGAAGGATCCAAACGATGAGATTTCCTTCA
GACCAAGGTTAACTGTTCGAAAACTAAAATTGCTGAAAATTGCTGTTGAACTCTTCTAGTTTTTTGTTGATTAATAAGCTTCCTAGGTTTGCTACTCTAAAGGAAGT

2 4
M R F P S

AOX1 promoter

860 880 900 920 940 960

ATTTTTACTGCAGTTTTATTCGCAGCATCCTCCGCATTAGCTGCTCCAGTCAACACTACAACAGAAGATGAAACGGCACAAATTCCGGCTGAAGCTGTCATCGGTTA
TAAAAATGACGTCAAAATAAGCGTCGTAGGAGGCGTAATCGACGAGGTCAGTTGTGATGTTGTCTTCTACTTTGCCGTGTTTAAGGCCGACTTCGACAGTAGCCAAT

6 8 10 12 14 16 18 20 22 24 26 28 30 32 34 36 38 40
I F T A V L F A A S S A L A A P V N T T T E D E T A Q I P A E A V I G Y

α-factor secretion signal

980 1,000 1,020 1,040 1,060

PsiI

CTCAGATTTAGAAGGGGATTTCGATGTTGCTGTTTTGCCATTTTCCAACAGCACAAATAACGGGTTATTGTTTATAAATACTACTATTGCCAGCATTGCTGCTAAAG
GAGTCTAAATCTTCCCCTAAAGCTACAACGACAAAACGGTAAAAGGTTGTCGTGTTTATTGCCCAATAACAAATATTTATGATGATAACGGTCGTAACGACGATTTC

42 44 46 48 50 52 54 56 58 60 62 64 66 68 70 72 74 76
S D L E G D F D V A V L P F S N S T N N G L L F I N T T I A S I A A K

α-factor secretion signal

1,080 1,100 1,120 1,140 1,160

PaeR7I
XhoI
TliI
SnaBI
BsaAI
EcoRI
SexAI

AAGAAGGGGTATCTCTCGAGAAAAGAGAGGCTGAAGCTTACGTAGAATTCGAAGTTCAGTTAGTTGAATCTGGTGGTGGCTCTGTCCAACCTGGTGGTTCTCTTACT
TTCTTCCCCATAGAGAGCTCTTTTCTCTCCGACTTCGAATGCATCTTAAGCTTCAAGTCAATCAACTTAGACCACCACCGAGACAGGTTGGACCACCAAGAGAATGA

78 80 82 84 86 88 90 92 94 96 98 100 102 104 106 108 110 112
E E G V S L E K R E A E A Y V E F E V Q L V E S G G G S V Q P G G S L T

α-factor secretion signal
CD4 nanobody

1,180 1,200 1,220 1,240 1,260 1,280

TTGTCCTGTGGTACTTCCGGTAGAACCTTCAACGTTATGGGATGGTTCAGACAAGCTCCTGGAAAGGAACGTGAGTTTGTCGCTGCTGTTAGGTGGTCTTCTACTGG
AACAGGACACCATGAAGGCCATCTTGGAAGTTGCAATACCCTACCAAGTCTGTTCGAGGACCTTTCCTTGCACTCAAACAGCGACGACAATCCACCAGAAGATGACC

114 116 118 120 122 124 126 128 130 132 134 136 138 140 142 144 146 148
L S C G T S G R T F N V M G W F R Q A P G K E R E F V A A V R W S S T G

CD4 nanobody

1,300 1,320 1,340 1,360 1,380

PsrI BstZ17I EcoRI

TATCTATTATACCCAATATGCTGACTCAGTTAAAAGTAGATTTACTATTTCAAGAGACAATGCAAAGAACACCGTATACTTGGAGATGAATTCACTGAAGCCAGAGG

ATAGATAATATGGGTTATACGACTGAGTCAATTTTCATCTAAATGATAAAGTTCTCTGTTACGTTTCTTGTGGCATATGAACCTCTACTTAAGTGACTTCGGTCTCC

150 152 154 156 158 160 162 164 166 168 170 172 174 176 178 180 182

I Y Y T Q Y A D S V K S R F T I S R D N A K N T V Y L E M N S L K P E

CD4 nanobody

1,400 1,420 1,440 1,460 1,480

Tth111I
PflFI

ACACGGCTGTCTACTACTGCGCAGCCGACACATATAACTCCAATCCAGCCAGATGGGACGGATATGACTTTCGAGGTCAAGGAACACAGGTGACAGTGTCCAGTGGA

TGTGCCGACAGATGATGACGCGTCGGCTGTGTATATTGAGGTTAGGTCGGTCTACCCTGCCTATACTGAAAGCTCCAGTTCCTTGTGTCCACTGTCACAGGTCACCT

184 186 188 190 192 194 196 198 200 202 204 206 208 210 212 214 216 218

D T A V Y Y C A A D T Y N S N P A R W D G Y D F R G Q G T Q V T V S S G

CD4 nanobody

1,500 1,520 1,540 1,560 1,580 1,600

NotI
EagI

GGCAGTGACTACAAAGATGACGACGATAAATGGCTGTTCCCTGTCGGAGACTATAAGGATGATGATGATAAGTAAGCGGCCGCGAATTAATTCGCCTTAGACATGAC

CCGTCACTGATGTTTCTACTGCTGCTATTTACCGACAAGGGACAGCCTCTGATATTCCTACTACTACTATTCATTCGCCGGCGCTTAATTAAGCGGAATCTGTACTG

220 222 224 226 228 230 232 234 236 238 240 242 244

G S D Y K D D D D K W L F P V G D Y K D D D D K *

FLAG barcode1 FLAG

1,620 1,640 1,660 1,680 1,700

AgeI

TGTTCCTCAGTTCAAGTTGGGCACTTACGAGAAGACCGGTCTTGCTAGATTCTAATCAAGAGGATGTCAGAATGCCATTTGCCTGAGAGATGCAGGCTTCATTTTTG

ACAAGGAGTCAAGTTCAACCCGTGAATGCTCTTCTGGCCAGAACGATCTAAGATTAGTTCTCCTACAGTCTTACGGTAAACGGACTCTCTACGTCCGAAGTAAAAAC

AOX1 terminator

1,720 1,740 1,760 1,780 1,800

BsiWI PvuII

ATACTTTTTTATTTGTAACCTATATAGTATAGGATTTTTTTTGTCATTTTGTTTCTTCTCGTACGAGCTTGCTCCTGATCAGCCTATCTCGCAGCTGATGAATATCT

TATGAAAAAATAAACATTGGATATATCATATCCTAAAAAAAACAGTAAAACAAAGAAGAGCATGCTCGAACGAGGACTAGTCGGATAGAGCGTCGACTACTTATAGA

AOX1 terminator

1,820 1,840 1,860 1,880 1,900 1,920

TGTGGTAGGGGTTTGGGAAAATCATTCGAGTTTGATGTTTTTCTTGGTATTTCCCACTCCTCTTCAGAGTACAGAAGATTAAGTGAGAAGTTCGTTTGTGCAAGCTT

ACACCATCCCCAAACCCTTTTAGTAAGCTCAAACTACAAAAAGAACCATAAAGGGTGAGGAGAAGTCTCATGTCTTCTAATTCACTCTTCAAGCAAACACGTTCGAA

AOX1 terminator

1,940 1,960 1,980 2,000 2,020

BcgI
ATCGATAAGCTTTAATGCGGTAGTTTATCACAGTTAAATTGCTAACGCAGTCAGGCACCGTGTATGAAATCTAACAATGCGCTCATCGTCATCCTCGGCACCGTCAC
TAGCTATTCGAAATTACGCCATCAAATAGTGTCAATTTAACGATTGCGTCAGTCCGTGGCACATACTTTAGATTGTTACGCGAGTAGCAGTAGGAGCCGTGGCAGTG
2,040 2,060 2,080 2,100 2,120 2,140

BmtI
BtgZI AfeI
EcoRV NheI
CCTGGATGCTGTAGGCATAGGCTTGGTTATGCCGGTACTGCCGGGCCTCTTGCGGGATATCGTCCATTCCGACAGCATCGCCAGTCACTATGGCGTGCTGCTAGCGC
GGACCTACGACATCCGTATCCGAACCAATACGGCCATGACGGCCCGGAGAACGCCCTATAGCAGGTAAGGCTGTCGTAGCGGTCAGTGATACCGCACGACGATCGCG
2,160 2,180 2,200 2,220 2,240

FspAI
TATATGCGTTGATGCAATTTCTATGCGCACCCGTTCTCGGAGCACTGTCCGACCGCTTTGGCCGCCGCCCAGTCCTGCTCGCTTCGCTACTTGGAGCCACTATCGAC
ATATACGCAACTACGTTAAAGATACGCGTGGGCAAGAGCCTCGTGACAGGCTGGCGAAACCGGCGGCGGGTCAGGACGAGCGAAGCGATGAACCTCGGTGATAGCTG
2,260 2,280 2,300 2,320 2,340

TACGCGATCATGGCGACCACACCCGTCCTGTGGATCTATCGAATCTAAATGTAAGTTAAAATCTCTAAATAATTAAATAAGTCCCAGTTTCTCCATACGAACCTTAA
ATGCGCTAGTACCGCTGGTGTGGGCAGGACACCTAGATAGCTTAGATTTACATTCAATTTTAGAGATTTATTAATTTATTCAGGGTCAAAGAGGTATGCTTGGAATT
2,360 2,380 2,400 2,420 2,440 2,460

XbaI MscI Bsu36I
CAGCATTGCGGTGAGCATCTAGACCTTCAACAGCAGCCAGATCCATCACTGCTTGGCCAATATGTTTCAGTCCCTCAGGAGTTACGTCTTGTGAAGTGATGAACTTC
GTCGTAACGCCACTCGTAGATCTGGAAGTTGTCGTCGGTCTAGGTAGTGACGAACCGGTTATACAAAGTCAGGGAGTCCTCAATGCAGAACACTTCACTACTTGAAG
2,480 2,500 2,520 2,540 2,560

KpnI
HpaI BsiWI Acc65I
TGGAAGGTTGCAGTGTTAACTCCGCTGTATTGACGGGCATATCCGTACGTTGGCAAAGTGTGGTTGGTACCGGAGGAGTAATCTCCACAACTCTCTGGAGAGTAGGC
ACCTTCCAACGTCACAATTGAGGCGACATAACTGCCCGTATAGGCATGCAACCGTTTCACACCAACCATGGCCTCCTCATTAGAGGTGTTGAGAGACCTCTCATCCG
2,580 2,600 2,620 2,640 2,660

AloI
ACCAACAAACACAGATCCAGCGTGTTGTACTTGATCAACATAAGAAGAAGCATTCTCGATTTGCAGGATCAAGTGTTCAGGAGCGTACTGATTGGACATTTCCAAAG
TGGTTGTTTGTGTCTAGGTCGCACAACATGAACTAGTTGTATTCTTCTTCGTAAGAGCTAAACGTCCTAGTTCACAAGTCCTCGCATGACTAACCTGTAAAGGTTTC
2,680 2,700 2,720 2,740 2,760 2,780

BsgI
CCTGCTCGTAGGTTGCAACCGATAGGGTTGTAGAGTGTGCAATACACTTGCGTACAATTTCAACCCTTGGCAACTGCACAGCTTGGTTGTGAACAGCATCTTCAATT
GGACGAGCATCCAACGTTGGCTATCCCAACATCTCACACGTTATGTGAACGCATGTTAAAGTTGGGAACCGTTGACGTGTCGAACCAACACTTGTCGTAGAAGTTAA
2,800 2,820 2,840 2,860 2,880

CTGGCAAGCTCCTTGTCTGTCATATCGACAGCCAACAGAATCACCTGGGAATCAATACCATGTTCAGCTTGAGACAGAAGGTCTGAGGCAACGAAATCTGGATCAGC
GACCGTTCGAGGAACAGACAGTATAGCTGTCGGTTGTCTTAGTGGACCCTTAGTTATGGTACAAGTCGAACTCTGTCTTCCAGACTCCGTTGCTTTAGACCTAGTCG

2,900 2,920 2,940 2,960 2,980

GTATTTATCAGCAATAACTAGAACTTCAGAAGGCCCAGCAGGCATGTCAATACTACACAGGGCTGATGTGTCATTTTGAACCATCATCTTGGCAGCAGTAACGAACT
CATAAATAGTCGTTATTGATCTTGAAGTCTTCCGGGTCGTCCGTACAGTTATGATGTGTCCCGACTACACAGTAAAACTTGGTAGTAGAACCGTCGTCATTGCTTGA

3,000 3,020 3,040 3,060 3,080 3,100

BmtI
NheI

GGTTTCCTGGACCAAATATTTTGTCACACTTAGGAACAGTTTCTGTTCCGTAAGCCATAGCAGCTACTGCCTGGGCGCCTCCTGCTAGCACGATACACTTAGCACCA
CCAAAGGACCTGGTTTATAAAACAGTGTGAATCCTTGTCAAAGACAAGGCATTCGGTATCGTCGATGACGGACCCGCGGAGGACGATCGTGCTATGTGAATCGTGGT

3,120 3,140 3,160 3,180 3,200

KpnI
Acc65I

ACCTTGTGGGCAACGTAGATGACTTCTGGGGTAAGGGTACCATCCTTCTTAGGTGGAGATGCAAAAACAATTTCTTTGCAACCAGCAACTTTGGCAGGAACACCCAG
TGGAACACCCGTTGCATCTACTGAAGACCCCATTCCCATGGTAGGAAGAATCCACCTCTACGTTTTTGTTAAAGAAACGTTGGTCGTTGAAACCGTCCTTGTGGGTC

3,220 3,240 3,260 3,280 3,300

CATCAGGGAAGTGGAAGGCAGAATTGCGGTTCCACCAGGAATATAGAGGCCAACTTTCTCAATAGGTCTTGCAAAACGAGAGCAGACTACACCAGGGCAAGTCTCAA
GTAGTCCCTTCACCTTCCGTCTTAACGCCAAGGTGGTCCTTATATCTCCGGTTGAAAGAGTTATCCAGAACGTTTTGCTCTCGTCTGATGTGGTCCCGTTCAGAGTT

3,320 3,340 3,360 3,380 3,400 3,420

CTTGCAACGTCTCCGTTAGTTGAGCTTCATGGAATTTCCTGACGTTATCTATAGAGAGATCAATGGCTCTCTTAACGTTATCTGGCAATTGCATAAGTTCCTCTGGG
GAACGTTGCAGAGGCAATCAACTCGAAGTACCTTAAAGGACTGCAATAGATATCTCTCTAGTTACCGAGAGAATTGCAATAGACCGTTAACGTATTCAAGGAGACCC

3,440 3,460 3,480 3,500 3,520

SalI

AAAGGAGCTTCTAACACAGGTGTCTTCAAAGCGACTCCATCAAACTTGGCAGTTAGTTCTAAAAGGGCTTTGTCACCATTTTGACGAACATTGTCGACAATTGGTTT
TTTCCTCGAAGATTGTGTCCACAGAAGTTTCGCTGAGGTAGTTTGAACCGTCAATCAAGATTTTCCCGAAACAGTGGTAAAACTGCTTGTAACAGCTGTTAACCAAA

3,540 3,560 3,580 3,600 3,620

StuI

GACTAATTCCATAATCTGTTCCGTTTTCTGGATAGGACGACGAAGGGCATCTTCAATTTCTTGTGAGGAGGCCTTAGAAACGTCAATTTTGCACAATTCAATACGAC
CTGATTAAGGTATTAGACAAGGCAAAAGACCTATCCTGCTGCTTCCCGTAGAAGTTAAAGAACACTCCTCCGGAATCTTTGCAGTTAAAACGTGTTAAGTTATGCTG

3,640 3,660 3,680 3,700 3,720 3,740

CTTCAGAAGGGACTTCTTTAGGTTTGGATTCTTCTTTAGGTTGTTCCTTGGTGTATCCTGGCTTGGCATCTCCTTTCCTTCTAGTGACCTTTAGGGACTTCATATCC
GAAGTCTTCCCTGAAGAAATCCAAACCTAAGAAGAAATCCAACAAGGAACCACATAGGACCGAACCGTAGAGGAAAGGAAGATCACTGGAAATCCCTGAAGTATAGG

3,760 3,780 3,800 3,820 3,840

AGGTTTCTCTCCACCTCGTCCAACGTCACACCGTACTTGGCACATCTAACTAATGCAAAATAAAATAAGTCAGCACATTCCCAGGCTATATCTTCCTTGGATTTAGC
TCCAAAGAGAGGTGGAGCAGGTTGCAGTGTGGCATGAACCGTGTAGATTGATTACGTTTTATTTTATTCAGTCGTGTAAGGGTCCGATATAGAAGGAACCTAAATCG

3,860 3,880 3,900 3,920 3,940

TTCTGCAAGTTCATCAGCTTCCTCCCTAATTTTAGCGTTCAACAAAACTTCGTCGTCAAATAACCGTTTGGTATAAGAACCTTCTGGAGCATTGCTCTTACGATCCC
AAGACGTTCAAGTAGTCGAAGGAGGGATTAAAATCGCAAGTTGTTTTGAAGCAGCAGTTTATTGGCAAACCATATTCTTGGAAGACCTCGTAACGAGAATGCTAGGG

3,960 3,980 4,000 4,020 4,040 4,060

DraIII NcoI MscI

ACAAGGTGGCTTCCATGGCTCTAAGACCCTTTGATTGGCCAAAACAGGAAGTGCGTTCCAAGTGACAGAAACCAACACCTGTTTGTTCAACCACAAATTTCAAGCAG
TGTTCCACCGAAGGTACCGAGATTCTGGGAAACTAACCGGTTTTGTCCTTCACGCAAGGTTCACTGTCTTTGGTTGTGGACAAACAAGTTGGTGTTTAAAGTTCGTC

4,080 4,100 4,120 4,140 4,160

TCTCCATCACAATCCAATTCGATACCCAGCAACTTTTGAGTTGCTCCAGATGTAGCACCTTTATACCACAAACCGTGACGACGAGATTGGTAGACTCCAGTTTGTGT
AGAGGTAGTGTTAGGTTAAGCTATGGGTCGTTGAAAACTCAACGAGGTCTACATCGTGGAAATATGGTGTTTGGCACTGCTGCTCTAACCATCTGAGGTCAAACACA

4,180 4,200 4,220 4,240 4,260 4,280

BspEI

CCTTATAGCCTCCGGAATAGACTTTTTGGACGAGTACACCAGGCCCAACGAGTAATTAGAAGAGTCAGCCACCAAAGTAGTGAATAGACCATCGGGGCGGTCAGTAG
GGAATATCGGAGGCCTTATCTGAAAAACCTGCTCATGTGGTCCGGGTTGCTCATTAATCTTCTCAGTCGGTGGTTTCATCACTTATCTGGTAGCCCCGCCAGTCATC

4,300 4,320 4,340 4,360 4,380

TCAAAGACGCCAACAAAATTTCACTGACAGGGAACTTTTTGACATCTTCAGAAAGTTCGTATTCAGTAGTCAATTGCCGAGCATCAATAATGGGGATTATACCAGAA
AGTTTCTGCGGTTGTTTTAAAGTGACTGTCCCTTGAAAAACTGTAGAAGTCTTTCAAGCATAAGTCATCAGTTAACGGCTCGTAGTTATTACCCCTAATATGGTCTT

4,400 4,420 4,440 4,460 4,480

GCAACAGTGGAAGTCACATCTACCAACTTTGCGGTCTCAGAAAAAGCATAAACAGTTCTACTACCGCCATTAGTGAAACTTTTCAAATCGCCCAGTGGAGAAGAAAA
CGTTGTCACCTTCAGTGTAGATGGTTGAAACGCCAGAGTCTTTTTCGTATTTGTCAAGATGATGGCGGTAATCACTTTGAAAAGTTTAGCGGGTCACCTCTTCTTTT

4,500 4,520 4,540 4,560 4,580 4,600

PpuMI

AGGCACAGCGATACTAGCATTAGCGGGCAAGGATGCAACTTTATCAACCAGGGTCCTATAGATAACCCTAGCGCCTGGGATCATCCTTTGGACAACTCTTTCTGCCA
TCCGTGTCGCTATGATCGTAATCGCCCGTTCCTACGTTGAAATAGTTGGTCCCAGGATATCTATTGGGATCGCGGACCCTAGTAGGAAACCTGTTGAGAAAGACGGT

4,620 4,640 4,660 4,680 4,700

BsrGI

AATCTAGGTCCAAAATCACTTCATTGATACCATTATTGTACAACTTGAGCAAGTTGTCGATCAGCTCCTCAAATTGGTCCTCTGTAACGGATGACTCAACTTGCACA
TTAGATCCAGGTTTTAGTGAAGTAACTATGGTAATAACATGTTGAACTCGTTCAACAGCTAGTCGAGGAGTTTAACCAGGAGACATTGCCTACTGAGTTGAACGTGT

4,720 4,740 4,760 4,780 4,800

PvuII BsgI

TTAACTTGAAGCTCAGTCGATTGAGTGAACTTGATCAGGTTGTGCAGCTGGTCAGCAGCATAGGGAAACACGGCTTTTCCTACCAAACTCAAGGAATTATCAAACTC
AATTGAACTTCGAGTCAGCTAACTCACTTGAACTAGTCCAACACGTCGACCAGTCGTCGTATCCCTTTGTGCCGAAAAGGATGGTTTGAGTTCCTTAATAGTTTGAG

4,820 4,840 4,860 4,880 4,900 4,920

TGCAACACTTGCGTATGCAGGTAGCAAGGGAAATGTCATACTTGAAGTCGGACAGTGAGTGTAGTCTTGAGAAATTCTGAAGCCGTATTTTTATTATCAGTGAGTCA
ACGTTGTGAACGCATACGTCCATCGTTCCCTTTACAGTATGAACTTCAGCCTGTCACTCACATCAGAACTCTTTAAGACTTCGGCATAAAAATAATAGTCACTCAGT

4,940 4,960 4,980 5,000 5,020

Bpu10I
SbfI BbvCI StuI

GTCATCAGGAGATCCTCTACGCCGGACGCATCGTGGCCGACCTGCAGGGGGGGGGGGGGCGCTGAGGTCTGCCTCGTGAAGAAGGTGTTGCTGACTCATACCAGGCC
CAGTAGTCCTCTAGGAGATGCGGCCTGCGTAGCACCGGCTGGACGTCCCCCCCCCCCCCGCGACTCCAGACGGAGCACTTCTTCCACAACGACTGAGTATGGTCCGG

5,040 5,060 5,080 5,100 5,120

BsaXI

TGAATCGCCCCATCATCCAGCCAGAAAGTGAGGGAGCCACGGTTGATGAGAGCTTTGTTGTAGGTGGACCAGTTGGTGATTTTGAACTTTTGCTTTGCCACGGAACG
ACTTAGCGGGGTAGTAGGTCGGTCTTTCACTCCCTCGGTGCCAACTACTCTCGAAACAACATCCACCTGGTCAACCACTAAAACTTGAAAACGAAACGGTGCCTTGC

5,140 5,160 5,180 5,200 5,220 5,240

GTCTGCGTTGTCGGGAAGATGCGTGATCTGATCCTTCAACTCAGCAAAAGTTCGATTTATTCAACAAAGCCGCCGTCCCGTCAAGTCAGCGTAATGCTCTGCCAGTG
CAGACGCAACAGCCCTTCTACGCACTAGACTAGGAAGTTGAGTCGTTTTCAAGCTAAATAAGTTGTTTCGGCGGCAGGGCAGTTCAGTCGCATTACGAGACGGTCAC

5,260 5,280 5,300 5,320 5,340

TTACAACCAATTAACCAATTCTGATTAGAAAAACTCATCGAGCATCAAATGAAACTGCAATTTATTCATATCAGGATTATCAATACCATATTTTTGAAAAAGCCGTT
AATGTTGGTTAATTGGTTAAGACTAATCTTTTTGAGTAGCTCGTAGTTTACTTTGACGTTAAATAAGTATAGTCCTAATAGTTATGGTATAAAAACTTTTTCGGCAA

5,360 5,380 5,400 5,420 5,440

TCTGTAATGAAGGAGAAAACTCACCGAGGCAGTTCCATAGGATGGCAAGATCCTGGTATCGGTCTGCGATTCCGACTCGTCCAACATCAATACAACCTATTAATTTC
AGACATTACTTCCTCTTTTGAGTGGCTCCGTCAAGGTATCCTACCGTTCTAGGACCATAGCCAGACGCTAAGGCTGAGCAGGTTGTAGTTATGTTGGATAATTAAAG

5,460 5,480 5,500 5,520 5,540 5,560

CCCTCGTCAAAAATAAGGTTATCAAGTGAGAAATCACCATGAGTGACGACTGAATCCGGTGAGAATGGCAAAAGCTTATGCATTTCTTTCCAGACTTGTTCAACAGG
GGGAGCAGTTTTTATTCCAATAGTTCACTCTTTAGTGGTACTCACTGCTGACTTAGGCCACTCTTACCGTTTTCGAATACGTAAAGAAAGGTCTGAACAAGTTGTCC

5,580 5,600 5,620 5,640 5,660

Bpu10I AsiSI
AjuI PvuI

CCAGCCATTACGCTCGTCATCAAAATCACTCGCATCAACCAAACCGTTATTCATTCGTGATTGCGCCTGAGCGAGACGAAATACGCGATCGCTGTTAAAAGGACAAT
GGTCGGTAATGCGAGCAGTAGTTTTAGTGAGCGTAGTTGGTTTGGCAATAAGTAAGCACTAACGCGGACTCGCTCTGCTTTATGCGCTAGCGACAATTTTCCTGTTA

5,680 5,700 5,720 5,740 5,760

XmaI
SmaI
TspMI

TACAAACAGGAATCGAATGCAACCGGCGCAGGAACACTGCCAGCGCATCAACAATATTTTCACCTGAATCAGGATATTCTTCTAATACCTGGAATGCTGTTTTCCCG
ATGTTTGTCCTTAGCTTACGTTGGCCGCGTCCTTGTGACGGTCGCGTAGTTGTTATAAAAGTGGACTTAGTCCTATAAGAAGATTATGGACCTTACGACAAAAGGGC

5,780 5,800 5,820 5,840 5,860 5,880

GGGATCGCAGTGGTGAGTAACCATGCATCATCAGGAGTACGGATAAAATGCTTGATGGTCGGAAGAGGCATAAATTCCGTCAGCCAGTTTAGTCTGACCATCTCATC
CCCTAGCGTCACCACTCATTGGTACGTAGTAGTCCTCATGCCTATTTTACGAACTACCAGCCTTCTCCGTATTTAAGGCAGTCGGTCAAATCAGACTGGTAGAGTAG

5,900 5,920 5,940 5,960 5,980

TGTAACATCATTGGCAACGCTACCTTTGCCATGTTTCAGAAACAACTCTGGCGCATCGGGCTTCCCATACAATCGATAGATTGTCGCACCTGATTGCCCGACATTAT
ACATTGTAGTAACCGTTGCGATGGAAACGGTACAAAGTCTTTGTTGAGACCGCGTAGCCCGAAGGGTATGTTAGCTATCTAACAGCGTGGACTAACGGGCTGTAATA

6,000 6,020 6,040 6,060 6,080

PaeR7I
PspXI
XhoI
TliI
NruI

CGCGAGCCCATTTATACCCATATAAATCAGCATCCATGTTGGAATTTAATCGCGGCCTCGAGCAAGACGTTTCCCGTTGAATATGGCTCATAACACCCCTTGTATTA
GCGCTCGGGTAAATATGGGTATATTTAGTCGTAGGTACAACCTTAAATTAGCGCCGGAGCTCGTTCTGCAAAGGGCAACTTATACCGAGTATTGTGGGGAACATAAT

6,100 6,120 6,140 6,160 6,180 6,200

DraIII

CTGTTTATGTAAGCAGACAGTTTTATTGTTCATGATGATATATTTTTATCTTGTGCAATGTAACATCAGAGATTTTGAGACACAACGTGGCTTTCCCCCCCCCCCCT
GACAAATACATTCGTCTGTCAAAATAACAAGTACTACTATATAAAAATAGAACACGTTACATTGTAGTCTCTAAAACTCTGTGTTGCACCGAAAGGGGGGGGGGGGA

6,220 6,240 6,260 6,280 6,300

SbfI SgrAI AfeI

GCAGGTCGGCATCACCGGCGCCACAGGTGCGGTTGCTGGCGCCTATATCGCCGACATCACCGATGGGGAAGATCGGGCTCGCCACTTCGGGCTCATGAGCGCTTGTT
CGTCCAGCCGTAGTGGCCGCGGTGTCCACGCCAACGACCGCGGATATAGCGGCTGTAGTGGCTACCCCTTCTAGCCCGAGCGGTGAAGCCCGAGTACTCGCGAACAA

6,320 6,340 6,360 6,380 6,400 6,420

SphI

TCGGCGTGGGTATGGTGGCAGGCCCCGTGGCCGGGGGACTGTTGGGCGCCATCTCCTTGCATGCACCATTCCTTGCGGCGGCGGTGCTCAACGGCCTCAACCTACTA
AGCCGCACCCATACCACCGTCCGGGGCACCGGCCCCCTGACAACCCGCGGTAGAGGAACGTACGTGGTAAGGAACGCCGCCGCCACGAGTTGCCGGAGTTGGATGAT

6,440 6,460 6,480 6,500 6,520

CTGGGCTGCTTCCTAATGCAGGAGTCGCATAAGGGAGAGCGTCGAGTATCTATGATTGGAAGTATGGGAATGGTGATACCCGCATTCTTCAGTGTCTTGAGGTCTCC
GACCCGACGAAGGATTACGTCCTCAGCGTATTCCCTCTCGCAGCTCATAGATACTAACCTTCATACCCTTACCACTATGGGCGTAAGAAGTCACAGAACTCCAGAGG

6,540 6,560 6,580 6,600 6,620

TATCAGATTATGCCCAACTAAAGCAACCGGAGGAGGAGATTTCATGGTAAATTTCTCTGACTTTTGGTCATCAGTAGACTCGAACTGTGAGACTATCTCGGTTATGA
ATAGTCTAATACGGGTTGATTTCGTTGGCCTCCTCCTCTAAAGTACCATTTAAAGAGACTGAAAACCAGTAGTCATCTGAGCTTGACACTCTGATAGAGCCAATACT

6,640 6,660 6,680 6,700 6,720 6,740

CAGCAGAAATGTCCTTCTTGGAGACAGTAAATGAAGTCCCACCAATAAAGAAATCCTTGTTATCAGGAACAAACTTCTTGTTTCGAACTTTTTCGGTGCCTTGAACT
GTCGTCTTTACAGGAAGAACCTCTGTCATTTACTTCAGGGTGGTTATTTCTTTAGGAACAATAGTCCTTGTTTGAAGAACAAAGCTTGAAAAAGCCACGGAACTTGA

6,760 6,780 6,800 6,820 6,840

ATAAAATGTAGAGTGGATATGTCGGGTAGGAATGGAGCGGGCAAATGCTTACCTTCTGGACCTTCAAGAGGTATGTAGGGTTTGTAGATACTGATGCCAACTTCAGT
TATTTTACATCTCACCTATACAGCCCATCCTTACCTCGCCCGTTTACGAATGGAAGACCTGGAAGTTCTCCATACATCCCAAACATCTATGACTACGGTTGAAGTCA

6,860 6,880 6,900 6,920 6,940

GACAACGTTGCTATTTCGTTCAAACCATTCCGAATCCAGAGAAATCAAAGTTGTTTGTCTACTATTGATCCAAGCCAGTGCGGTCTTGAAACTGACAATAGTGTGCT
CTGTTGCAACGATAAAGCAAGTTTGGTAAGGCTTAGGTCTCTTTAGTTTCAACAAACAGATGATAACTAGGTTCGGTCACGCCAGAACTTTGACTGTTATCACACGA

6,960 6,980 7,000 7,020 7,040 7,060

CGTGTTTTGAGGTCATCTTTGTATGAATAAATCTAGTCTTTGATCTAAATAATCTTGACGAGCCAAGGCGATAAATACCCAAATCTAAAACTCTTTTAAAACGTTAA
GCACAAAACTCCAGTAGAAACATACTTATTTAGATCAGAAACTAGATTTATTAGAACTGCTCGGTTCCGCTATTTATGGGTTTAGATTTTGAGAAAATTTTGCAATT

7,080 7,100 7,120 7,140 7,160

AAGGACAAGTATGTCTGCCTGTATTAAACCCCAAATCAGCTCGTAGTCTGATCCTCATCAACTTGAGGGGCACTATCTTGTTTTAGAGAAATTTGCGGAGATGCGAT
TTCCTGTTCATACAGACGGACATAATTTGGGGTTTAGTCGAGCATCAGACTAGGAGTAGTTGAACTCCCCGTGATAGAACAAAATCTCTTTAAACGCCTCTACGCTA

7,180 7,200 7,220 7,240 7,260

EcoRV BglII

ATCGAGAAAAAGGTACGCTGATTTTAAACGTGAAATTTATCTCAAGATCTCTGCCTCGCGCGTTTCGGTGATGACGGTGAAAACCTCTGACACATGCAGCTCCCGGA
TAGCTCTTTTTCCATGCGACTAAAATTTGCACTTTAAATAGAGTTCTAGAGACGGAGCGCGCAAAGCCACTACTGCCACTTTTGGAGACTGTGTACGTCGAGGGCCT

7,280 7,300 7,320 7,340 7,360 7,380

BsaAI
Tth111I
PflFI

GACGGTCACAGCTTGTCTGTAAGCGGATGCCGGGAGCAGACAAGCCCGTCAGGGCGCGTCAGCGGGTGTTGGCGGGTGTCGGGGCGCAGCCATGACCCAGTCACGTA
CTGCCAGTGTCGAACAGACATTCGCCTACGGCCCTCGTCTGTTCGGGCAGTCCCGCGCAGTCGCCCACAACCGCCCACAGCCCCGCGTCGGTACTGGGTCAGTGCAT

7,400 7,420 7,440 7,460 7,480

BstZ17I NdeI

GCGATAGCGGAGTGTATACTGGCTTAACTATGCGGCATCAGAGCAGATTGTACTGAGAGTGCACCATATGCGGTGTGAAATACCGCACAGATGCGTAAGGAGAAAAT
CGCTATCGCCTCACATATGACCGAATTGATACGCCGTAGTCTCGTCTAACATGACTCTCACGTGGTATACGCCACACTTTATGGCGTGTCTACGCATTCCTCTTTTA

7,500 7,520 7,540 7,560 7,580

BspQI
SapI

ACCGCATCAGGCGCTCTTCCGCTTCCTCGCTCACTGACTCGCTGCGCTCGGTCGTTCGGCTGCGGCGAGCGGTATCAGCTCACTCAAAGGCGGTAATACGGTTATCC
TGGCGTAGTCCGCGAGAAGGCGAAGGAGCGAGTGACTGAGCGACGCGAGCCAGCAAGCCGACGCCGCTCGCCATAGTCGAGTGAGTTTCCGCCATTATGCCAATAGG

7,600 7,620 7,640 7,660 7,680 7,700

AflIII
PciI

ACAGAATCAGGGGATAACGCAGGAAAGAACATGTGAGCAAAAGGCCAGCAAAAGGCCAGGAACCGTAAAAAGGCCGCGTTGCTGGCGTTTTTCCATAGGCTCCGCCC
TGTCTTAGTCCCCTATTGCGTCCTTTCTTGTACACTCGTTTTCCGGTCGTTTTCCGGTCCTTGGCATTTTTCCGGCGCAACGACCGCAAAAAGGTATCCGAGGCGGG

ColE1 origin

7,720 7,740 7,760 7,780 7,800

CCCTGACGAGCATCACAAAAATCGACGCTCAAGTCAGAGGTGGCGAAACCCGACAGGACTATAAAGATACCAGGCGTTTCCCCCTGGAAGCTCCCTCGTGCGCTCTC
GGGACTGCTCGTAGTGTTTTTAGCTGCGAGTTCAGTCTCCACCGCTTTGGGCTGTCCTGATATTTCTATGGTCCGCAAAGGGGGACCTTCGAGGGAGCACGCGAGAG

ColE1 origin

7,820 7,840 7,860 7,880 7,900

CTGTTCCGACCCTGCCGCTTACCGGATACCTGTCCGCCTTTCTCCCTTCGGGAAGCGTGGCGCTTTCTCAATGCTCACGCTGTAGGTATCTCAGTTCGGTGTAGGTC
GACAAGGCTGGGACGGCGAATGGCCTATGGACAGGCGGAAAGAGGGAAGCCCTTCGCACCGCGAAAGAGTTACGAGTGCGACATCCATAGAGTCAAGCCACATCCAG

ColE1 origin

7,920 7,940 7,960 7,980 8,000 8,020

GTTCGCTCCAAGCTGGGCTGTGTGCACGAACCCCCCGTTCAGCCCGACCGCTGCGCCTTATCCGGTAACTATCGTCTTGAGTCCAACCCGGTAAGACACGACTTATC
CAAGCGAGGTTCGACCCGACACACGTGCTTGGGGGGCAAGTCGGGCTGGCGACGCGGAATAGGCCATTGATAGCAGAACTCAGGTTGGGCCATTCTGTGCTGAATAG

ColE1 origin

8,040 8,060 8,080 8,100 8,120

GCCACTGGCAGCAGCCACTGGTAACAGGATTAGCAGAGCGAGGTATGTAGGCGGTGCTACAGAGTTCTTGAAGTGGTGGCCTAACTACGGCTACACTAGAAGGACAG
CGGTGACCGTCGTCGGTGACCATTGTCCTAATCGTCTCGCTCCATACATCCGCCACGATGTCTCAAGAACTTCACCACCGGATTGATGCCGATGTGATCTTCCTGTC

ColE1 origin

8,140 8,160 8,180 8,200 8,220

TATTTGGTATCTGCGCTCTGCTGAAGCCAGTTACCTTCGGAAAAAGAGTTGGTAGCTCTTGATCCGGCAAACAAACCACCGCTGGTAGCGGTGGTTTTTTTGTTTGC
ATAAACCATAGACGCGAGACGACTTCGGTCAATGGAAGCCTTTTTCTCAACCATCGAGAACTAGGCCGTTTGTTTGGTGGCGACCATCGCCACCAAAAAAACAAACG

ColE1 origin

8,240 8,260 8,280 8,300 8,320 8,340

AAGCAGCAGATTACGCGCAGAAAAAAAGGATCTCAAGAAGATCCTTTGATCTTTTCTACGGGGTCTGACGCTCAGTGGAACGAAAACTCACGTTAAGGGATTTTGGT
TTCGTCGTCTAATGCGCGTCTTTTTTTCCTAGAGTTCTTCTAGGAAACTAGAAAAGATGCCCCAGACTGCGAGTCACCTTGCTTTTGAGTGCAATTCCCTAAAACCA

ColE1 origin

8,360 8,380 8,400 8,420 8,440

CATGAGATTATCAAAAAGGATCTTCACCTAGATCCTTTTAAATTAAAAATGAAGTTTTAAATCAATCTAAAGTATATATGAGTAAACTTGGTCTGACAGTTACCAAT
GTACTCTAATAGTTTTTCCTAGAAGTGGATCTAGGAAAATTTAATTTTTACTTCAAAATTTAGTTAGATTTCATATATACTCATTTGAACCAGACTGTCAATGGTTA

8,460 8,480 8,500 8,520 8,540 8,560

GCTTAATCAGTGAGGCACCTATCTCAGCGATCTGTCTATTTCGTTCATCCATAGTTGCCTGACTCCCCGTCGTGTAGATAACTACGATACGGGAGGGCTTACCATCT
CGAATTAGTCACTCCGTGGATAGAGTCGCTAGACAGATAAAGCAAGTAGGTATCAACGGACTGAGGGGCAGCACATCTATTGATGCTATGCCCTCCCGAATGGTAGA

AmpR

8,580 8,600 8,620 8,640 8,660

GGCCCCAGTGCTGCAATGATACCGCGAGACCCACGCTCACCGGCTCCAGATTTATCAGCAATAAACCAGCCAGCCGGAAGGGCCGAGCGCAGAAGTGGTCCTGCAAC
CCGGGGTCACGACGTTACTATGGCGCTCTGGGTGCGAGTGGCCGAGGTCTAAATAGTCGTTATTTGGTCGGTCGGCCTTCCCGGCTCGCGTCTTCACCAGGACGTTG

AmpR

8,680 8,700 8,720 8,740 8,760

TTTATCCGCCTCCATCCAGTCTATTAATTGTTGCCGGGAAGCTAGAGTAAGTAGTTCGCCAGTTAATAGTTTGCGCAACGTTGTTGCCATTGCTGCAGGCATCGTGG
AAATAGGCGGAGGTAGGTCAGATAATTAACAACGGCCCTTCGATCTCATTCATCAAGCGGTCAATTATCAAACGCGTTGCAACAACGGTAACGACGTCCGTAGCACC

AmpR

8,780 8,800 8,820 8,840 8,860 8,880

TGTCACGCTCGTCGTTTGGTATGGCTTCATTCAGCTCCGGTTCCCAACGATCAAGGCGAGTTACATGATCCCCCATGTTGTGCAAAAAAGCGGTTAGCTCCTTCGGT
ACAGTGCGAGCAGCAAACCATACCGAAGTAAGTCGAGGCCAAGGGTTGCTAGTTCCGCTCAATGTACTAGGGGGTACAACACGTTTTTTCGCCAATCGAGGAAGCCA

AmpR

8,900 8,920 8,940 8,960 8,980

PvuI

CCTCCGATCGTTGTCAGAAGTAAGTTGGCCGCAGTGTTATCACTCATGGTTATGGCAGCACTGCATAATTCTCTTACTGTCATGCCATCCGTAAGATGCTTTTCTGT
GGAGGCTAGCAACAGTCTTCATTCAACCGGCGTCACAATAGTGAGTACCAATACCGTCGTGACGTATTAAGAGAATGACAGTACGGTAGGCATTCTACGAAAAGACA

AmpR

9,000 9,020 9,040 9,060 9,080

ScaI BcgI

GACTGGTGAGTACTCAACCAAGTCATTCTGAGAATAGTGTATGCGGCGACCGAGTTGCTCTTGCCCGGCGTCAACACGGGATAATACCGCGCCACATAGCAGAACTT
CTGACCACTCATGAGTTGGTTCAGTAAGACTCTTATCACATACGCCGCTGGCTCAACGAGAACGGGCCGCAGTTGTGCCCTATTATGGCGCGGTGTATCGTCTTGAA

AmpR

9,100 9,120 9,140 9,160 9,180 9,200

TAAAAGTGCTCATCATTGGAAAACGTTCTTCGGGGCGAAAACTCTCAAGGATCTTACCGCTGTTGAGATCCAGTTCGATGTAACCCACTCGTGCACCCAACTGATCT
ATTTTCACGAGTAGTAACCTTTTGCAAGAAGCCCCGCTTTTGAGAGTTCCTAGAATGGCGACAACTCTAGGTCAAGCTACATTGGGTGAGCACGTGGGTTGACTAGA

AmpR

9,220 9,240 9,260 9,280 9,300

```
TCAGCATCTTTTACTTTCACCAGCGTTTCTGGGTGAGCAAAAACAGGAAGGCAAAATGCCGCAAAAAAGGGAATAAGGGCGACACGGAAATGTTGAATACTCATACT
AGTCGTAGAAAATGAAAGTGGTCGCAAAGACCCACTCGTTTTTGTCCTTCCGTTTTACGGCGTTTTTTCCCTTATTCCCGCTGTGCCTTTACAACTTATGAGTATGA
          9,320               9,340               9,360               9,380               9,400

CTTCCTTTTTCAATATTATTGAAGCATTTATCAGGGTTATTGTCTCATGAGCGGATACATATTTGAATGTATTTAGAAAAATAAACAAATAGGGGTTCCGCGCACAT
GAAGGAAAAAGTTATAATAACTTCGTAAATAGTCCCAATAACAGAGTACTCGCCTATGTATAAACTTACATAAATCTTTTTATTTGTTTATCCCCAAGGCGCGTGTA
 9,420               9,440               9,460               9,480               9,500               9,520

                        AatII
                      ZraI
TTCCCCGAAAAGTGCCACCTGACGTCTAAGAAACCATTATTATCATGACATTAACCTATAAAAATAGGCGTATCACGAGGCCCTTTCGTCTTCAAGAATTAATTCTC
AAGGGGCTTTTCACGGTGGACTGCAGATTCTTTGGTAATAATAGTACTGTAATTGGATATTTTTATCCGCATAGTGCTCCGGGAAAGCAGAAGTTCTTAATTAAGAG
                9,540               9,560               9,580               9,600               9,620

ATGTTTGACAGCTTATCATCGATAAGCTGACTCATGTTGGTATTGTGAAATAGACGCAGATCGGGAACACTGAAAAATAACAGTTATTATTCG
TACAAACTGTCGAATAGTAGCTATTCGACTGAGTACAACCATAACACTTTATCTGCGTCTAGCCCTTGTGACTTTTTATTGTCAATAATAAGC
         9,640     9,650     9,660     9,670     9,680     9,690     9,700     9,710     9,720
```

Anti-GFP-FLAG (9654 bp)

BglII AleI
AGATCTAACATCCAAAGACGAAAGGTTGAATGAAACCTTTTTGCCATCCGACATCCACAGGTCCATTCTCACACATAAGTGCCAAACGCAACAGGAGGGGATACACT
TCTAGATTGTAGGTTTCTGCTTTCCAACTTACTTTGGAAAAACGGTAGGCTGTAGGTGTCCAGGTAAGAGTGTGTATTCACGGTTTGCGTTGTCCTCCCCTATGTGA
AOX1 promoter
20 40 60 80 100

SacI
PpuMI Eco53kI
AGCAGCAGACCGTTGCAAACGCAGGACCTCCACTCCTCTTCTCCTCAACACCCACTTTTGCCATCGAAAAACCAGCCCAGTTATTGGGCTTGATTGGAGCTCGCTCA
TCGTCGTCTGGCAACGTTTGCGTCCTGGAGGTGAGGAGAAGAGGAGTTGTGGGTGAAAACGGTAGCTTTTTGGTCGGGTCAATAACCCGAACTAACCTCGAGCGAGT
AOX1 promoter
120 140 160 180 200

TTCCAATTCCTTCTATTAGGCTACTAACACCATGACTTTATTAGCCTGTCTATCCTGGCCCCCCTGGCGAGGTTCATGTTTGTTTATTTCCGAATGCAACAAGCTCC
AAGGTTAAGGAAGATAATCCGATGATTGTGGTACTGAAATAATCGGACAGATAGGACCGGGGGGACCGCTCCAAGTACAAACAAATAAAGGCTTACGTTGTTCGAGG
AOX1 promoter
220 240 260 280 300 320

PmeI
GCATTACACCCGAACATCACTCCAGATGAGGGCTTTCTGAGTGTGGGGTCAAATAGTTTCATGTTCCCCAAATGGCCCAAAACTGACAGTTTAAACGCTGTCTTGGA
CGTAATGTGGGCTTGTAGTGAGGTCTACTCCCGAAAGACTCACACCCCAGTTTATCAAAGTACAAGGGGTTTACCGGGTTTTGACTGTCAAATTTGCGACAGAACCT
AOX1 promoter
340 360 380 400 420

ACCTAATATGACAAAAGCGTGATCTCATCCAAGATGAACTAAGTTTGGTTCGTTGAAATGCTAACGGCCAGTTGGTCAAAAAGAAACTTCCAAAAGTCGCCATACCG
TGGATTATACTGTTTTCGCACTAGAGTAGGTTCTACTTGATTCAAACCAAGCAACTTTACGATTGCCGGTCAACCAGTTTTTCTTTGAAGGTTTTCAGCGGTATGGC
AOX1 promoter
440 460 480 500 520

BlpI
TTTGTCTTGTTTGGTATTGATTGACGAATGCTCAAAAATAATCTCATTAATGCTTAGCGCAGTCTCTCTATCGCTTCTGAACCCCGGTGCACCTGTGCCGAAACGCA
AAACAGAACAAACCATAACTAACTGCTTACGAGTTTTTATTAGAGTAATTACGAATCGCGTCAGAGAGATAGCGAAGACTTGGGGCCACGTGGACACGGCTTTGCGT
AOX1 promoter
540 560 580 600 620 640

XcmI
AATGGGGAAACACCCGCTTTTTGGATGATTATGCATTGTCTCCACATTGTATGCTTCCAAGATTCTGGTGGGAATACTGCTGATAGCCTAACGTTCATGATCAAAAT
TTACCCCTTTGTGGGCGAAAAACCTACTAATACGTAACAGAGGTGTAACATACGAAGGTTCTAAGACCACCCTTATGACGACTATCGGATTGCAAGTACTAGTTTTA
AOX1 promoter
660 680 700 720 740

TTAACTGTTCTAACCCCTACTTGACAGCAATATATAAACAGAAGGAAGCTGCCCTGTCTTAAACCTTTTTTTTTATCATCATTATTAGCTTACTTTCATAATTGCGA
AATTGACAAGATTGGGGATGAACTGTCGTTATATATTTGTCTTCCTTCGACGGGACAGAATTTGGAAAAAAAAATAGTAGTAATAATCGAATGAAAGTATTAACGCT

AOX1 promoter

760 780 800 820 840

BamHI
BstBI

CTGGTTCCAATTGACAAGCTTTTGATTTTAACGACTTTTAACGACAACTTGAGAAGATCAAAAAACAACTAATTATTCGAAGGATCCAAACGATGAGATTTCCTTCA
GACCAAGGTTAACTGTTCGAAAACTAAAATTGCTGAAAATTGCTGTTGAACTCTTCTAGTTTTTTGTTGATTAATAAGCTTCCTAGGTTTGCTACTCTAAAGGAAGT

2 4
M R F P S

AOX1 promoter

860 880 900 920 940 960

ATTTTTACTGCAGTTTTATTCGCAGCATCCTCCGCATTAGCTGCTCCAGTCAACACTACAACAGAAGATGAAACGGCACAAATTCCGGCTGAAGCTGTCATCGGTTA
TAAAAATGACGTCAAAATAAGCGTCGTAGGAGGCGTAATCGACGAGGTCAGTTGTGATGTTGTCTTCTACTTTGCCGTGTTTAAGGCCGACTTCGACAGTAGCCAAT

6 8 10 12 14 16 18 20 22 24 26 28 30 32 34 36 38 40
I F T A V L F A A S S A L A A P V N T T T E D E T A Q I P A E A V I G Y

α-factor secretion signal

980 1,000 1,020 1,040 1,060

PsiI

CTCAGATTTAGAAGGGGATTTCGATGTTGCTGTTTTGCCATTTTCCAACAGCACAAATAACGGGTTATTGTTTATAAATACTACTATTGCCAGCATTGCTGCTAAAG
GAGTCTAAATCTTCCCCTAAAGCTACAACGACAAAACGGTAAAAGGTTGTCGTGTTTATTGCCCAATAACAAATATTTATGATGATAACGGTCGTAACGACGATTTC

42 44 46 48 50 52 54 56 58 60 62 64 66 68 70 72 74 76
S D L E G D F D V A V L P F S N S T N N G L L F I N T T I A S I A A K

α-factor secretion signal

1,080 1,100 1,120 1,140 1,160

PaeR7I
XhoI
TliI
EcoRI
SnaBI

AAGAAGGGGTATCTCTCGAGAAAAGAGAGGCTGAAGCTTACGTAGAATTCATGCAGGTTCAATTGGTTGAATCTGGTGGTGCCCTTGTTCAACCTGGAGGATCACTA
TTCTTCCCCATAGAGAGCTCTTTTCTCTCCGACTTCGAATGCATCTTAAGTACGTCCAAGTTAACCAACTTAGACCACCACGGGAACAAGTTGGACCTCCTAGTGAT

78 80 82 84 86 88 90 92 94 96 98 100 102 104 106 108 110 112
E E G V S L E K R E A E A Y V E F M Q V Q L V E S G G A L V Q P G G S L

α-factor secretion signal GFP nanobody

1,180 1,200 1,220 1,240 1,260 1,280

BspEI HpaI

AGATTATCCTGTGCTGCCTCCGGATTTCCTGTTAACAGGTACTCCATGAGATGGTATAGACAGGCTCCAGGTAAAGAAAGAGAGTGGGTCGCTGGTATGTCATCTGC
TCTAATAGGACACGACGGAGGCCTAAAGGACAATTGTCCATGAGGTACTCTACCATATCTGTCCGAGGTCCATTTCTTTCTCTCACCCAGCGACCATACAGTAGACG

114 116 118 120 122 124 126 128 130 132 134 136 138 140 142 144 146 148
R L S C A A S G F P V N R Y S M R W Y R Q A P G K E R E W V A G M S S A

GFP nanobody

1,300 1,320 1,340 1,360 1,380

EcoRI

TGGAGATAGATCCTCATACGAGGATTCTGTCAAAGGAAGGTTTACTATTAGTCGTGACGACGCACGTAATACCGTTTATTTGCAAATGAATTCTCTGAAACCAGAAG
ACCTCTATCTAGGAGTATGCTCCTAAGACAGTTTCCTTCCAAATGATAATCAGCACTGCTGCGTGCATTATGGCAAATAAACGTTTACTTAAGAGACTTTGGTCTTC

150 152 154 156 158 160 162 164 166 168 170 172 174 176 178 180 182
G D R S S Y E D S V K G R F T I S R D D A R N T V Y L Q M N S L K P E

GFP nanobody

1,400 1,420 1,440 1,460 1,480

Tth111I
ScaI PflFI BaeI

ACACTGCAGTATATTACTGCAACGTCAACGTAGGTTTCGAGTACTGGGGCCAAGGTACACAGGTGACGGTGTCTAGTGGCGGTAGTGACTATAAAGATGATGATGAT
TGTGACGTCATATAATGACGTTGCAGTTGCATCCAAAGCTCATGACCCCGGTTCCATGTGTCCACTGCCACAGATCACCGCCATCACTGATATTTCTACTACTACTA

184 186 188 190 192 194 196 198 200 202 204 206 208 210 212 214 216 218
D T A V Y Y C N V N V G F E Y W G Q G T Q V T V S S G G S D Y K D D D D

GFP nanobody FLAG

1,500 1,520 1,540 1,560 1,580 1,600

NotI
EagI AgeI

AAGTAAGCGGCCGCGAATTAATTCGCCTTAGACATGACTGTTCCTCAGTTCAAGTTGGGCACTTACGAGAAGACCGGTCTTGCTAGATTCTAATCAAGAGGATGTCA
TTCATTCGCCGGCGCTTAATTAAGCGGAATCTGTACTGACAAGGAGTCAAGTTCAACCCGTGAATGCTCTTCTGGCCAGAACGATCTAAGATTAGTTCTCCTACAGT

220
K *

1,620 1,640 1,660 1,680 1,700

BsiWI

GAATGCCATTTGCCTGAGAGATGCAGGCTTCATTTTTGATACTTTTTTATTTGTAACCTATATAGTATAGGATTTTTTTTGTCATTTTGTTTCTTCTCGTACGAGCT
CTTACGGTAAACGGACTCTCTACGTCCGAAGTAAAAACTATGAAAAAATAAACATTGGATATATCATATCCTAAAAAAAACAGTAAAACAAAGAAGAGCATGCTCGA

AOX1 terminator

1,720 1,740 1,760 1,780 1,800

PvuII

TGCTCCTGATCAGCCTATCTCGCAGCTGATGAATATCTTGTGGTAGGGGTTTGGGAAAATCATTCGAGTTTGATGTTTTTCTTGGTATTTCCCACTCCTCTTCAGAG

ACGAGGACTAGTCGGATAGAGCGTCGACTACTTATAGAACACCATCCCCAAACCCTTTTAGTAAGCTCAAACTACAAAAAGAACCATAAAGGGTGAGGAGAAGTCTC

AOX1 terminator

1,820 1,840 1,860 1,880 1,900 1,920

BcgI

TACAGAAGATTAAGTGAGAAGTTCGTTTGTGCAAGCTTATCGATAAGCTTTAATGCGGTAGTTTATCACAGTTAAATTGCTAACGCAGTCAGGCACCGTGTATGAAA

ATGTCTTCTAATTCACTCTTCAAGCAAACACGTTCGAATAGCTATTCGAAATTACGCCATCAAATAGTGTCAATTTAACGATTGCGTCAGTCCGTGGCACATACTTT

1,940 1,960 1,980 2,000 2,020

BtgZI
EcoRV

TCTAACAATGCGCTCATCGTCATCCTCGGCACCGTCACCCTGGATGCTGTAGGCATAGGCTTGGTTATGCCGGTACTGCCGGGCCTCTTGCGGGATATCGTCCATTC

AGATTGTTACGCGAGTAGCAGTAGGAGCCGTGGCAGTGGGACCTACGACATCCGTATCCGAACCAATACGGCCATGACGGCCCGGAGAACGCCCTATAGCAGGTAAG

2,040 2,060 2,080 2,100 2,120 2,140

AfeI
BmtI
NheI
FspAI
FspI

CGACAGCATCGCCAGTCACTATGGCGTGCTGCTAGCGCTATATGCGTTGATGCAATTTCTATGCGCACCCGTTCTCGGAGCACTGTCCGACCGCTTTGGCCGCCGCC

GCTGTCGTAGCGGTCAGTGATACCGCACGACGATCGCGATATACGCAACTACGTTAAAGATACGCGTGGGCAAGAGCCTCGTGACAGGCTGGCGAAACCGGCGGCGG

2,160 2,180 2,200 2,220 2,240

CAGTCCTGCTCGCTTCGCTACTTGGAGCCACTATCGACTACGCGATCATGGCGACCACACCCGTCCTGTGGATCTATCGAATCTAAATGTAAGTTAAAATCTCTAAA

GTCAGGACGAGCGAAGCGATGAACCTCGGTGATAGCTGATGCGCTAGTACCGCTGGTGTGGGCAGGACACCTAGATAGCTTAGATTTACATTCAATTTTAGAGATTT

2,260 2,280 2,300 2,320 2,340

XbaI
MscI

TAATTAAATAAGTCCCAGTTTCTCCATACGAACCTTAACAGCATTGCGGTGAGCATCTAGACCTTCAACAGCAGCCAGATCCATCACTGCTTGGCCAATATGTTTCA

ATTAATTTATTCAGGGTCAAAGAGGTATGCTTGGAATTGTCGTAACGCCACTCGTAGATCTGGAAGTTGTCGTCGGTCTAGGTAGTGACGAACCGGTTATACAAAGT

2,360 2,380 2,400 2,420 2,440 2,460

Bsu36I
HpaI
BsiWI
Acc65I

GTCCCTCAGGAGTTACGTCTTGTGAAGTGATGAACTTCTGGAAGGTTGCAGTGTTAACTCCGCTGTATTGACGGGCATATCCGTACGTTGGCAAAGTGTGGTTGGTA

CAGGGAGTCCTCAATGCAGAACACTTCACTACTTGAAGACCTTCCAACGTCACAATTGAGGCGACATAACTGCCCGTATAGGCATGCAACCGTTTCACACCAACCAT

2,480 2,500 2,520 2,540 2,560

KpnI

CCGGAGGAGTAATCTCCACAACTCTCTGGAGAGTAGGCACCAACAAACACAGATCCAGCGTGTTGTACTTGATCAACATAAGAAGAAGCATTCTCGATTTGCAGGAT

GGCCTCCTCATTAGAGGTGTTGAGAGACCTCTCATCCGTGGTTGTTTGTGTCTAGGTCGCACAACATGAACTAGTTGTATTCTTCTTCGTAAGAGCTAAACGTCCTA

2,580 2,600 2,620 2,640 2,660

```
                             AloI                                                                                                          BsgI
CAAGTGTTCAGGAGCGTACTGATTGGACATTTCCAAAGCCTGCTCGTAGGTTGCAACCGATAGGGTTGTAGAGTGTGCAATACACTTGCGTACAATTTCAACCCTTG
GTTCACAAGTCCTCGCATGACTAACCTGTAAAGGTTTCGGACGAGCATCCAACGTTGGCTATCCCAACATCTCACACGTTATGTGAACGCATGTTAAAGTTGGGAAC
    2,680                2,700                2,720                2,740                2,760                2,780

GCAACTGCACAGCTTGGTTGTGAACAGCATCTTCAATTCTGGCAAGCTCCTTGTCTGTCATATCGACAGCCAACAGAATCACCTGGGAATCAATACCATGTTCAGCT
CGTTGACGTGTCGAACCAACACTTGTCGTAGAAGTTAAGACCGTTCGAGGAACAGACAGTATAGCTGTCGGTTGTCTTAGTGGACCCTTAGTTATGGTACAAGTCGA
                 2,800                2,820                2,840                2,860                2,880

TGAGACAGAAGGTCTGAGGCAACGAAATCTGGATCAGCGTATTTATCAGCAATAACTAGAACTTCAGAAGGCCCAGCAGGCATGTCAATACTACACAGGGCTGATGT
ACTCTGTCTTCCAGACTCCGTTGCTTTAGACCTAGTCGCATAAATAGTCGTTATTGATCTTGAAGTCTTCCGGGTCGTCCGTACAGTTATGATGTGTCCCGACTACA
          2,900                2,920                2,940                2,960                2,980

                                                    PfoI
GTCATTTTGAACCATCATCTTGGCAGCAGTAACGAACTGGTTTCCTGGACCAAATATTTTGTCACACTTAGGAACAGTTTCTGTTCCGTAAGCCATAGCAGCTACTG
CAGTAAAACTTGGTAGTAGAACCGTCGTCATTGCTTGACCAAAGGACCTGGTTTATAAAACAGTGTGAATCCTTGTCAAAGACAAGGCATTCGGTATCGTCGATGAC
 3,000                3,020                3,040                3,060                3,080                3,100

                         BmtI                                                                          KpnI
                    NheI                                                                           Acc65I
CCTGGGCGCCTCCTGCTAGCACGATACACTTAGCACCAACCTTGTGGGCAACGTAGATGACTTCTGGGGTAAGGGTACCATCCTTCTTAGGTGGAGATGCAAAAACA
GGACCCGCGGAGGACGATCGTGCTATGTGAATCGTGGTTGGAACACCCGTTGCATCTACTGAAGACCCCATTCCCATGGTAGGAAGAATCCACCTCTACGTTTTTGT
                    3,120                3,140                3,160                3,180                3,200

ATTTCTTTGCAACCAGCAACTTTGGCAGGAACACCCAGCATCAGGGAAGTGGAAGGCAGAATTGCGGTTCCACCAGGAATATAGAGGCCAACTTTCTCAATAGGTCT
TAAAGAAACGTTGGTCGTTGAAACCGTCCTTGTGGGTCGTAGTCCCTTCACCTTCCGTCTTAACGCCAAGGTGGTCCTTATATCTCCGGTTGAAAGAGTTATCCAGA
         3,220                3,240                3,260                3,280                3,300

TGCAAAACGAGAGCAGACTACACCAGGGCAAGTCTCAACTTGCAACGTCTCCGTTAGTTGAGCTTCATGGAATTTCCTGACGTTATCTATAGAGAGATCAATGGCTC
ACGTTTTGCTCTCGTCTGATGTGGTCCCGTTCAGAGTTGAACGTTGCAGAGGCAATCAACTCGAAGTACCTTAAAGGACTGCAATAGATATCTCTCTAGTTACCGAG
 3,320                3,340                3,360                3,380                3,400                3,420

TCTTAACGTTATCTGGCAATTGCATAAGTTCCTCTGGGAAAGGAGCTTCTAACACAGGTGTCTTCAAAGCGACTCCATCAAACTTGGCAGTTAGTTCTAAAAGGGCT
AGAATTGCAATAGACCGTTAACGTATTCAAGGAGACCCTTTCCTCGAAGATTGTGTCCACAGAAGTTTCGCTGAGGTAGTTTGAACCGTCAATCAAGATTTTCCCGA
               3,440                3,460                3,480                3,500                3,520

                              SalI
TTGTCACCATTTTGACGAACATTGTCGACAATTGGTTTGACTAATTCCATAATCTGTTCCGTTTTCTGGATAGGACGACGAAGGGCATCTTCAATTTCTTGTGAGGA
AACAGTGGTAAAACTGCTTGTAACAGCTGTTAACCAAACTGATTAAGGTATTAGACAAGGCAAAAGACCTATCCTGCTGCTTCCCGTAGAAGTTAAAGAACACTCCT
        3,540                3,560                3,580                3,600                3,620
```

StuI

GGCCTTAGAAACGTCAATTTTGCACAATTCAATACGACCTTCAGAAGGGACTTCTTTAGGTTTGGATTCTTCTTTAGGTTGTTCCTTGGTGTATCCTGGCTTGGCAT
CCGGAATCTTTGCAGTTAAAACGTGTTAAGTTATGCTGGAAGTCTTCCCTGAAGAAATCCAAACCTAAGAAGAAATCCAACAAGGAACCACATAGGACCGAACCGTA

3,640 3,660 3,680 3,700 3,720 3,740

CTCCTTTCCTTCTAGTGACCTTTAGGGACTTCATATCCAGGTTTCTCTCCACCTCGTCCAACGTCACACCGTACTTGGCACATCTAACTAATGCAAAATAAAATAAG
GAGGAAAGGAAGATCACTGGAAATCCCTGAAGTATAGGTCCAAAGAGAGGTGGAGCAGGTTGCAGTGTGGCATGAACCGTGTAGATTGATTACGTTTTATTTTATTC

3,760 3,780 3,800 3,820 3,840

TCAGCACATTCCCAGGCTATATCTTCCTTGGATTTAGCTTCTGCAAGTTCATCAGCTTCCTCCCTAATTTTAGCGTTCAACAAAACTTCGTCGTCAAATAACCGTTT
AGTCGTGTAAGGGTCCGATATAGAAGGAACCTAAATCGAAGACGTTCAAGTAGTCGAAGGAGGGATTAAAATCGCAAGTTGTTTTGAAGCAGCAGTTTATTGGCAAA

3,860 3,880 3,900 3,920 3,940

DraIII NcoI MscI

GGTATAAGAACCTTCTGGAGCATTGCTCTTACGATCCCACAAGGTGGCTTCCATGGCTCTAAGACCCTTTGATTGGCCAAAACAGGAAGTGCGTTCCAAGTGACAGA
CCATATTCTTGGAAGACCTCGTAACGAGAATGCTAGGGTGTTCCACCGAAGGTACCGAGATTCTGGGAAACTAACCGGTTTTGTCCTTCACGCAAGGTTCACTGTCT

3,960 3,980 4,000 4,020 4,040 4,060

AACCAACACCTGTTTGTTCAACCACAAATTTCAAGCAGTCTCCATCACAATCCAATTCGATACCCAGCAACTTTTGAGTTGCTCCAGATGTAGCACCTTTATACCAC
TTGGTTGTGGACAAACAAGTTGGTGTTTAAAGTTCGTCAGAGGTAGTGTTAGGTTAAGCTATGGGTCGTTGAAAACTCAACGAGGTCTACATCGTGGAAATATGGTG

4,080 4,100 4,120 4,140 4,160

BspEI

AAACCGTGACGACGAGATTGGTAGACTCCAGTTTGTGTCCTTATAGCCTCCGGAATAGACTTTTTGGACGAGTACACCAGGCCCAACGAGTAATTAGAAGAGTCAGC
TTTGGCACTGCTGCTCTAACCATCTGAGGTCAAACACAGGAATATCGGAGGCCTTATCTGAAAAACCTGCTCATGTGGTCCGGGTTGCTCATTAATCTTCTCAGTCG

4,180 4,200 4,220 4,240 4,260 4,280

CACCAAAGTAGTGAATAGACCATCGGGGCGGTCAGTAGTCAAAGACGCCAACAAAATTTCACTGACAGGGAACTTTTTGACATCTTCAGAAAGTTCGTATTCAGTAG
GTGGTTTCATCACTTATCTGGTAGCCCCGCCAGTCATCAGTTTCTGCGGTTGTTTTAAAGTGACTGTCCCTTGAAAAACTGTAGAAGTCTTTCAAGCATAAGTCATC

4,300 4,320 4,340 4,360 4,380

TCAATTGCCGAGCATCAATAATGGGGATTATACCAGAAGCAACAGTGGAAGTCACATCTACCAACTTTGCGGTCTCAGAAAAAGCATAAACAGTTCTACTACCGCCA
AGTTAACGGCTCGTAGTTATTACCCCTAATATGGTCTTCGTTGTCACCTTCAGTGTAGATGGTTGAAACGCCAGAGTCTTTTTCGTATTTGTCAAGATGATGGCGGT

4,400 4,420 4,440 4,460 4,480

PpuMI

TTAGTGAAACTTTTCAAATCGCCCAGTGGAGAAGAAAAAGGCACAGCGATACTAGCATTAGCGGGCAAGGATGCAACTTTATCAACCAGGGTCCTATAGATAACCCT
AATCACTTTGAAAAGTTTAGCGGGTCACCTCTTCTTTTTCCGTGTCGCTATGATCGTAATCGCCCGTTCCTACGTTGAAATAGTTGGTCCCAGGATATCTATTGGGA

4,500 4,520 4,540 4,560 4,580 4,600

BsrGI

AGCGCCTGGGATCATCCTTTGGACAACTCTTTCTGCCAAATCTAGGTCCAAAATCACTTCATTGATACCATTATTGTACAACTTGAGCAAGTTGTCGATCAGCTCCT
TCGCGGACCCTAGTAGGAAACCTGTTGAGAAAGACGGTTTAGATCCAGGTTTTAGTGAAGTAACTATGGTAATAACATGTTGAACTCGTTCAACAGCTAGTCGAGGA

4,620 4,640 4,660 4,680 4,700

PvuII BsgI

CAAATTGGTCCTCTGTAACGGATGACTCAACTTGCACATTAACTTGAAGCTCAGTCGATTGAGTGAACTTGATCAGGTTGTGCAGCTGGTCAGCAGCATAGGGAAAC
GTTTAACCAGGAGACATTGCCTACTGAGTTGAACGTGTAATTGAACTTCGAGTCAGCTAACTCACTTGAACTAGTCCAACACGTCGACCAGTCGTCGTATCCCTTTG

4,720 4,740 4,760 4,780 4,800

ACGGCTTTTCCTACCAAACTCAAGGAATTATCAAACTCTGCAACACTTGCGTATGCAGGTAGCAAGGGAAATGTCATACTTGAAGTCGGACAGTGAGTGTAGTCTTG
TGCCGAAAAGGATGGTTTGAGTTCCTTAATAGTTTGAGACGTTGTGAACGCATACGTCCATCGTTCCCTTTACAGTATGAACTTCAGCCTGTCACTCACATCAGAAC

4,820 4,840 4,860 4,880 4,900 4,920

Bpu10I
SbfI BbvCI

AGAAATTCTGAAGCCGTATTTTTATTATCAGTGAGTCAGTCATCAGGAGATCCTCTACGCCGGACGCATCGTGGCCGACCTGCAGGGGGGGGGGGGGCGCTGAGGTC
TCTTTAAGACTTCGGCATAAAAATAATAGTCACTCAGTCAGTAGTCCTCTAGGAGATGCGGCCTGCGTAGCACCGGCTGGACGTCCCCCCCCCCCCCGCGACTCCAG

4,940 4,960 4,980 5,000 5,020

StuI

TGCCTCGTGAAGAAGGTGTTGCTGACTCATACCAGGCCTGAATCGCCCCATCATCCAGCCAGAAAGTGAGGGAGCCACGGTTGATGAGAGCTTTGTTGTAGGTGGAC
ACGGAGCACTTCTTCCACAACGACTGAGTATGGTCCGGACTTAGCGGGGTAGTAGGTCGGTCTTTCACTCCCTCGGTGCCAACTACTCTCGAAACAACATCCACCTG

5,040 5,060 5,080 5,100 5,120

CAGTTGGTGATTTTGAACTTTTGCTTTGCCACGGAACGGTCTGCGTTGTCGGGAAGATGCGTGATCTGATCCTTCAACTCAGCAAAAGTTCGATTTATTCAACAAAG
GTCAACCACTAAAACTTGAAAACGAAACGGTGCCTTGCCAGACGCAACAGCCCTTCTACGCACTAGACTAGGAAGTTGAGTCGTTTTCAAGCTAAATAAGTTGTTTC

5,140 5,160 5,180 5,200 5,220 5,240

CCGCCGTCCCGTCAAGTCAGCGTAATGCTCTGCCAGTGTTACAACCAATTAACCAATTCTGATTAGAAAAACTCATCGAGCATCAAATGAAACTGCAATTTATTCAT
GGCGGCAGGGCAGTTCAGTCGCATTACGAGACGGTCACAATGTTGGTTAATTGGTTAAGACTAATCTTTTTGAGTAGCTCGTAGTTTACTTTGACGTTAAATAAGTA

5,260 5,280 5,300 5,320 5,340

ATCAGGATTATCAATACCATATTTTTGAAAAAGCCGTTTCTGTAATGAAGGAGAAAACTCACCGAGGCAGTTCCATAGGATGGCAAGATCCTGGTATCGGTCTGCGA
TAGTCCTAATAGTTATGGTATAAAAACTTTTTCGGCAAAGACATTACTTCCTCTTTTGAGTGGCTCCGTCAAGGTATCCTACCGTTCTAGGACCATAGCCAGACGCT

5,360 5,380 5,400 5,420 5,440

TTCCGACTCGTCCAACATCAATACAACCTATTAATTTCCCCTCGTCAAAAATAAGGTTATCAAGTGAGAAATCACCATGAGTGACGACTGAATCCGGTGAGAATGGC
AAGGCTGAGCAGGTTGTAGTTATGTTGGATAATTAAAGGGGAGCAGTTTTTATTCCAATAGTTCACTCTTTAGTGGTACTCACTGCTGACTTAGGCCACTCTTACCG

5,460 5,480 5,500 5,520 5,540 5,560

AjuI
Bpu10I
AAAAGCTTATGCATTTCTTTCCAGACTTGTTCAACAGGCCAGCCATTACGCTCGTCATCAAAATCACTCGCATCAACCAAACCGTTATTCATTCGTGATTGCGCCTG
TTTTCGAATACGTAAAGAAAGGTCTGAACAAGTTGTCCGGTCGGTAATGCGAGCAGTAGTTTTAGTGAGCGTAGTTGGTTTGGCAATAAGTAAGCACTAACGCGGAC
5,580 5,600 5,620 5,640 5,660

AsiSI
PvuI
AGCGAGACGAAATACGCGATCGCTGTTAAAAGGACAATTACAAACAGGAATCGAATGCAACCGGCGCAGGAACACTGCCAGCGCATCAACAATATTTTCACCTGAAT
TCGCTCTGCTTTATGCGCTAGCGACAATTTTCCTGTTAATGTTTGTCCTTAGCTTACGTTGGCCGCGTCCTTGTGACGGTCGCGTAGTTGTTATAAAAGTGGACTTA
5,680 5,700 5,720 5,740 5,760

SmaI
TspMI
XmaI
CAGGATATTCTTCTAATACCTGGAATGCTGTTTTCCCGGGGATCGCAGTGGTGAGTAACCATGCATCATCAGGAGTACGGATAAAATGCTTGATGGTCGGAAGAGGC
GTCCTATAAGAAGATTATGGACCTTACGACAAAAGGGCCCCTAGCGTCACCACTCATTGGTACGTAGTAGTCCTCATGCCTATTTTACGAACTACCAGCCTTCTCCG
5,780 5,800 5,820 5,840 5,860 5,880

ATAAATTCCGTCAGCCAGTTTAGTCTGACCATCTCATCTGTAACATCATTGGCAACGCTACCTTTGCCATGTTTCAGAAACAACTCTGGCGCATCGGGCTTCCCATA
TATTTAAGGCAGTCGGTCAAATCAGACTGGTAGAGTAGACATTGTAGTAACCGTTGCGATGGAAACGGTACAAAGTCTTTGTTGAGACCGCGTAGCCCGAAGGGTAT
5,900 5,920 5,940 5,960 5,980

PaeR7I
PspXI
XhoI
NruI TliI
CAATCGATAGATTGTCGCACCTGATTGCCCGACATTATCGCGAGCCCATTTATACCCATATAAATCAGCATCCATGTTGGAATTTAATCGCGGCCTCGAGCAAGACG
GTTAGCTATCTAACAGCGTGGACTAACGGGCTGTAATAGCGCTCGGGTAAATATGGGTATATTTAGTCGTAGGTACAACCTTAAATTAGCGCCGGAGCTCGTTCTGC
6,000 6,020 6,040 6,060 6,080

TTTCCCGTTGAATATGGCTCATAACACCCCTTGTATTACTGTTTATGTAAGCAGACAGTTTTATTGTTCATGATGATATATTTTTATCTTGTGCAATGTAACATCAG
AAAGGGCAACTTATACCGAGTATTGTGGGGAACATAATGACAAATACATTCGTCTGTCAAAATAACAAGTACTACTATATAAAAATAGAACACGTTACATTGTAGTC
6,100 6,120 6,140 6,160 6,180 6,200

DraIII SbfI SgrAI
AGATTTTGAGACACAACGTGGCTTTCCCCCCCCCCCCCTGCAGGTCGGCATCACCGGCGCCACAGGTGCGGTTGCTGGCGCCTATATCGCCGACATCACCGATGGGGA
TCTAAAACTCTGTGTTGCACCGAAAGGGGGGGGGGGGGACGTCCAGCCGTAGTGGCCGCGGTGTCCACGCCAACGACCGCGGATATAGCGGCTGTAGTGGCTACCCCT
6,220 6,240 6,260 6,280 6,300

AfeI SphI
AGATCGGGCTCGCCACTTCGGGCTCATGAGCGCTTGTTTCGGCGTGGGTATGGTGGCAGGCCCCGTGGCCGGGGGACTGTTGGGCGCCATCTCCTTGCATGCACCAT
TCTAGCCCGAGCGGTGAAGCCCGAGTACTCGCGAACAAAGCCGCACCCATACCACCGTCCGGGGCACCGGCCCCCTGACAACCCGCGGTAGAGGAACGTACGTGGTA
6,320 6,340 6,360 6,380 6,400 6,420

TCCTTGCGGCGGCGGTGCTCAACGGCCTCAACCTACTACTGGGCTGCTTCCTAATGCAGGAGTCGCATAAGGGAGAGCGTCGAGTATCTATGATTGGAAGTATGGGA
AGGAACGCCGCCGCCACGAGTTGCCGGAGTTGGATGATGACCCGACGAAGGATTACGTCCTCAGCGTATTCCCTCTCGCAGCTCATAGATACTAACCTTCATACCCT

6,440 6,460 6,480 6,500 6,520

ATGGTGATACCCGCATTCTTCAGTGTCTTGAGGTCTCCTATCAGATTATGCCCAACTAAAGCAACCGGAGGAGGAGATTTCATGGTAAATTTCTCTGACTTTTGGTC
TACCACTATGGGCGTAAGAAGTCACAGAACTCCAGAGGATAGTCTAATACGGGTTGATTTCGTTGGCCTCCTCCTCTAAAGTACCATTTAAAGAGACTGAAAACCAG

6,540 6,560 6,580 6,600 6,620

ATCAGTAGACTCGAACTGTGAGACTATCTCGGTTATGACAGCAGAAATGTCCTTCTTGGAGACAGTAAATGAAGTCCCACCAATAAAGAAATCCTTGTTATCAGGAA
TAGTCATCTGAGCTTGACACTCTGATAGAGCCAATACTGTCGTCTTTACAGGAAGAACCTCTGTCATTTACTTCAGGGTGGTTATTTCTTTAGGAACAATAGTCCTT

6,640 6,660 6,680 6,700 6,720 6,740

BstBI

CAAACTTCTTGTTTCGAACTTTTTCGGTGCCTTGAACTATAAAATGTAGAGTGGATATGTCGGGTAGGAATGGAGCGGGCAAATGCTTACCTTCTGGACCTTCAAGA
GTTTGAAGAACAAAGCTTGAAAAAGCCACGGAACTTGATATTTTACATCTCACCTATACAGCCCATCCTTACCTCGCCCGTTTACGAATGGAAGACCTGGAAGTTCT

6,760 6,780 6,800 6,820 6,840

GGTATGTAGGGTTTGTAGATACTGATGCCAACTTCAGTGACAACGTTGCTATTTCGTTCAAACCATTCCGAATCCAGAGAAATCAAAGTTGTTTGTCTACTATTGAT
CCATACATCCCAAACATCTATGACTACGGTTGAAGTCACTGTTGCAACGATAAAGCAAGTTTGGTAAGGCTTAGGTCTCTTTAGTTTCAACAAACAGATGATAACTA

6,860 6,880 6,900 6,920 6,940

CCAAGCCAGTGCGGTCTTGAAACTGACAATAGTGTGCTCGTGTTTTGAGGTCATCTTTGTATGAATAAATCTAGTCTTTGATCTAAATAATCTTGACGAGCCAAGGC
GGTTCGGTCACGCCAGAACTTTGACTGTTATCACACGAGCACAAAACTCCAGTAGAAACATACTTATTTAGATCAGAAACTAGATTTATTAGAACTGCTCGGTTCCG

6,960 6,980 7,000 7,020 7,040 7,060

GATAAATACCCAAATCTAAAACTCTTTTAAAACGTTAAAAGGACAAGTATGTCTGCCTGTATTAAACCCCAAATCAGCTCGTAGTCTGATCCTCATCAACTTGAGGG
CTATTTATGGGTTTAGATTTTGAGAAAATTTTGCAATTTTCCTGTTCATACAGACGGACATAATTTGGGGTTTAGTCGAGCATCAGACTAGGAGTAGTTGAACTCCC

7,080 7,100 7,120 7,140 7,160

EcoRV BglII

GCACTATCTTGTTTTAGAGAAATTTGCGGAGATGCGATATCGAGAAAAAGGTACGCTGATTTTAAACGTGAAATTTATCTCAAGATCTCTGCCTCGCGCGTTTCGGT
CGTGATAGAACAAAATCTCTTTAAACGCCTCTACGCTATAGCTCTTTTTCCATGCGACTAAAATTTGCACTTTAAATAGAGTTCTAGAGACGGAGCGCGCAAAGCCA

7,180 7,200 7,220 7,240 7,260

PfoI

GATGACGGTGAAAACCTCTGACACATGCAGCTCCCGGAGACGGTCACAGCTTGTCTGTAAGCGGATGCCGGGAGCAGACAAGCCCGTCAGGGCGCGTCAGCGGGTGT
CTACTGCCACTTTTGGAGACTGTGTACGTCGAGGGCCTCTGCCAGTGTCGAACAGACATTCGCCTACGGCCCTCGTCTGTTCGGGCAGTCCCGCGCAGTCGCCCACA

7,280 7,300 7,320 7,340 7,360 7,380

Tth111I
PflFI BstZ17I NdeI

TGGCGGGTGTCGGGGCGCAGCCATGACCCAGTCACGTAGCGATAGCGGAGTGTATACTGGCTTAACTATGCGGCATCAGAGCAGATTGTACTGAGAGTGCACCATAT
ACCGCCCACAGCCCCGCGTCGGTACTGGGTCAGTGCATCGCTATCGCCTCACATATGACCGAATTGATACGCCGTAGTCTCGTCTAACATGACTCTCACGTGGTATA

7,400 7,420 7,440 7,460 7,480

BspQI
SapI

GCGGTGTGAAATACCGCACAGATGCGTAAGGAGAAAATACCGCATCAGGCGCTCTTCCGCTTCCTCGCTCACTGACTCGCTGCGCTCGGTCGTTCGGCTGCGGCGAG
CGCCACACTTTATGGCGTGTCTACGCATTCCTCTTTTATGGCGTAGTCCGCGAGAAGGCGAAGGAGCGAGTGACTGAGCGACGCGAGCCAGCAAGCCGACGCCGCTC

7,500 7,520 7,540 7,560 7,580

AflIII
PciI

CGGTATCAGCTCACTCAAAGGCGGTAATACGGTTATCCACAGAATCAGGGGATAACGCAGGAAAGAACATGTGAGCAAAAGGCCAGCAAAAGGCCAGGAACCGTAAA
GCCATAGTCGAGTGAGTTTCCGCCATTATGCCAATAGGTGTCTTAGTCCCCTATTGCGTCCTTTCTTGTACACTCGTTTTCCGGTCGTTTTCCGGTCCTTGGCATTT

7,600 7,620 7,640 7,660 7,680 7,700

AAGGCCGCGTTGCTGGCGTTTTTCCATAGGCTCCGCCCCCCTGACGAGCATCACAAAAATCGACGCTCAAGTCAGAGGTGGCGAAACCCGACAGGACTATAAAGATA
TTCCGGCGCAACGACCGCAAAAAGGTATCCGAGGCGGGGGGACTGCTCGTAGTGTTTTTAGCTGCGAGTTCAGTCTCCACCGCTTTGGGCTGTCCTGATATTTCTAT

ColE1 origin

7,720 7,740 7,760 7,780 7,800

CCAGGCGTTTCCCCCTGGAAGCTCCCTCGTGCGCTCTCCTGTTCCGACCCTGCCGCTTACCGGATACCTGTCCGCCTTTCTCCCTTCGGGAAGCGTGGCGCTTTCTC
GGTCCGCAAAGGGGGACCTTCGAGGGAGCACGCGAGAGGACAAGGCTGGGACGGCGAATGGCCTATGGACAGGCGGAAAGAGGGAAGCCCTTCGCACCGCGAAAGAG

ColE1 origin

7,820 7,840 7,860 7,880 7,900

AATGCTCACGCTGTAGGTATCTCAGTTCGGTGTAGGTCGTTCGCTCCAAGCTGGGCTGTGTGCACGAACCCCCCGTTCAGCCCGACCGCTGCGCCTTATCCGGTAAC
TTACGAGTGCGACATCCATAGAGTCAAGCCACATCCAGCAAGCGAGGTTCGACCCGACACACGTGCTTGGGGGGCAAGTCGGGCTGGCGACGCGGAATAGGCCATTG

ColE1 origin

7,920 7,940 7,960 7,980 8,000 8,020

TATCGTCTTGAGTCCAACCCGGTAAGACACGACTTATCGCCACTGGCAGCAGCCACTGGTAACAGGATTAGCAGAGCGAGGTATGTAGGCGGTGCTACAGAGTTCTT
ATAGCAGAACTCAGGTTGGGCCATTCTGTGCTGAATAGCGGTGACCGTCGTCGGTGACCATTGTCCTAATCGTCTCGCTCCATACATCCGCCACGATGTCTCAAGAA

ColE1 origin

8,040 8,060 8,080 8,100 8,120

GAAGTGGTGGCCTAACTACGGCTACACTAGAAGGACAGTATTTGGTATCTGCGCTCTGCTGAAGCCAGTTACCTTCGGAAAAAGAGTTGGTAGCTCTTGATCCGGCA
CTTCACCACCGGATTGATGCCGATGTGATCTTCCTGTCATAAACCATAGACGCGAGACGACTTCGGTCAATGGAAGCCTTTTTCTCAACCATCGAGAACTAGGCCGT

ColE1 origin

8,140 8,160 8,180 8,200 8,220

AACAAACCACCGCTGGTAGCGGTGGTTTTTTTGTTTGCAAGCAGCAGATTACGCGCAGAAAAAAAGGATCTCAAGAAGATCCTTTGATCTTTTCTACGGGGTCTGAC
TTGTTTGGTGGCGACCATCGCCACCAAAAAAACAAACGTTCGTCGTCTAATGCGCGTCTTTTTTTCCTAGAGTTCTTCTAGGAAACTAGAAAAGATGCCCCAGACTG

ColE1 origin

8,240 8,260 8,280 8,300 8,320 8,340

GCTCAGTGGAACGAAAACTCACGTTAAGGGATTTTGGTCATGAGATTATCAAAAAGGATCTTCACCTAGATCCTTTTAAATTAAAAATGAAGTTTTAAATCAATCTA
CGAGTCACCTTGCTTTTGAGTGCAATTCCCTAAAACCAGTACTCTAATAGTTTTTCCTAGAAGTGGATCTAGGAAAATTTAATTTTTACTTCAAAATTTAGTTAGAT
ColE1 origin
8,360 8,380 8,400 8,420 8,440

AAGTATATATGAGTAAACTTGGTCTGACAGTTACCAATGCTTAATCAGTGAGGCACCTATCTCAGCGATCTGTCTATTTCGTTCATCCATAGTTGCCTGACTCCCCG
TTCATATATACTCATTTGAACCAGACTGTCAATGGTTACGAATTAGTCACTCCGTGGATAGAGTCGCTAGACAGATAAAGCAAGTAGGTATCAACGGACTGAGGGGC
AmpR
8,460 8,480 8,500 8,520 8,540 8,560

TCGTGTAGATAACTACGATACGGGAGGGCTTACCATCTGGCCCCAGTGCTGCAATGATACCGCGAGACCCACGCTCACCGGCTCCAGATTTATCAGCAATAAACCAG
AGCACATCTATTGATGCTATGCCCTCCCGAATGGTAGACCGGGGTCACGACGTTACTATGGCGCTCTGGGTGCGAGTGGCCGAGGTCTAAATAGTCGTTATTTGGTC
AmpR
8,580 8,600 8,620 8,640 8,660

CCAGCCGGAAGGGCCGAGCGCAGAAGTGGTCCTGCAACTTTATCCGCCTCCATCCAGTCTATTAATTGTTGCCGGGAAGCTAGAGTAAGTAGTTCGCCAGTTAATAG
GGTCGGCCTTCCCGGCTCGCGTCTTCACCAGGACGTTGAAATAGGCGGAGGTAGGTCAGATAATTAACAACGGCCCTTCGATCTCATTCATCAAGCGGTCAATTATC
AmpR
8,680 8,700 8,720 8,740 8,760

FspI
TTTGCGCAACGTTGTTGCCATTGCTGCAGGCATCGTGGTGTCACGCTCGTCGTTTGGTATGGCTTCATTCAGCTCCGGTTCCCAACGATCAAGGCGAGTTACATGAT
AAACGCGTTGCAACAACGGTAACGACGTCCGTAGCACCACAGTGCGAGCAGCAAACCATACCGAAGTAAGTCGAGGCCAAGGGTTGCTAGTTCCGCTCAATGTACTA
AmpR
8,780 8,800 8,820 8,840 8,860 8,880

PvuI
CCCCCATGTTGTGCAAAAAAGCGGTTAGCTCCTTCGGTCCTCCGATCGTTGTCAGAAGTAAGTTGGCCGCAGTGTTATCACTCATGGTTATGGCAGCACTGCATAAT
GGGGGTACAACACGTTTTTTCGCCAATCGAGGAAGCCAGGAGGCTAGCAACAGTCTTCATTCAACCGGCGTCACAATAGTGAGTACCAATACCGTCGTGACGTATTA
AmpR
8,900 8,920 8,940 8,960 8,980

ScaI BcgI
TCTCTTACTGTCATGCCATCCGTAAGATGCTTTTCTGTGACTGGTGAGTACTCAACCAAGTCATTCTGAGAATAGTGTATGCGGCGACCGAGTTGCTCTTGCCCGGC
AGAGAATGACAGTACGGTAGGCATTCTACGAAAAGACACTGACCACTCATGAGTTGGTTCAGTAAGACTCTTATCACATACGCCGCTGGCTCAACGAGAACGGGCCG
AmpR
9,000 9,020 9,040 9,060 9,080

GTCAACACGGGATAATACCGCGCCACATAGCAGAACTTTAAAAGTGCTCATCATTGGAAAACGTTCTTCGGGGCGAAAACTCTCAAGGATCTTACCGCTGTTGAGAT
CAGTTGTGCCCTATTATGGCGCGGTGTATCGTCTTGAAATTTTCACGAGTAGTAACCTTTTGCAAGAAGCCCCGCTTTTGAGAGTTCCTAGAATGGCGACAACTCTA
AmpR
9,100 9,120 9,140 9,160 9,180 9,200

```
CCAGTTCGATGTAACCCACTCGTGCACCCAACTGATCTTCAGCATCTTTTACTTTCACCAGCGTTTCTGGGTGAGCAAAAACAGGAAGGCAAAATGCCGCAAAAAAG
GGTCAAGCTACATTGGGTGAGCACGTGGGTTGACTAGAAGTCGTAGAAAATGAAAGTGGTCGCAAAGACCCACTCGTTTTTGTCCTTCCGTTTTACGGCGTTTTTTC
                 9,220               9,240               9,260               9,280               9,300

GGAATAAGGGCGACACGGAAATGTTGAATACTCATACTCTTCCTTTTTCAATATTATTGAAGCATTTATCAGGGTTATTGTCTCATGAGCGGATACATATTTGAATG
CCTTATTCCCGCTGTGCCTTTACAACTTATGAGTATGAGAAGGAAAAAGTTATAATAACTTCGTAAATAGTCCCAATAACAGAGTACTCGCCTATGTATAAACTTAC
          9,320               9,340               9,360               9,380               9,400

                                                                       AatII
                                                                     ZraI
TATTTAGAAAAATAAACAAATAGGGGTTCCGCGCACATTTCCCCGAAAAGTGCCACCTGACGTCTAAGAAACCATTATTATCATGACATTAACCTATAAAAATAGGC
ATAAATCTTTTTATTTGTTTATCCCCAAGGCGCGTGTAAAGGGGCTTTTCACGGTGGACTGCAGATTCTTTGGTAATAATAGTACTGTAATTGGATATTTTTATCCG
 9,420               9,440               9,460               9,480               9,500               9,520

GTATCACGAGGCCCTTTCGTCTTCAAGAATTAATTCTCATGTTTGACAGCTTATCATCGATAAGCTGACTCATGTTGGTATTGTGAAATAGACGCAGATCGGGAACA
CATAGTGCTCCGGGAAAGCAGAAGTTCTTAATTAAGAGTACAAACTGTCGAATAGTAGCTATTCGACTGAGTACAACCATAACACTTTATCTGCGTCTAGCCCTTGT
                9,540               9,560               9,580               9,600               9,620

CTGAAAAATAACAGTTATTATTCG
GACTTTTTATTGTCAATAATAAGC
         9,640     9,650
```

Anti-GFP-FLAG-Barcode2 (9696 bp)

BglII AleI

AGATCTAACATCCAAAGACGAAAGGTTGAATGAAACCTTTTTGCCATCCGACATCCACAGGTCCATTCTCACACATAAGTGCCAAACGCAACAGGAGGGGATACACT
TCTAGATTGTAGGTTTCTGCTTTCCAACTTACTTTGGAAAAACGGTAGGCTGTAGGTGTCCAGGTAAGAGTGTGTATTCACGGTTTGCGTTGTCCTCCCCTATGTGA

AOX1 promoter

20 40 60 80 100

SacI
PpuMI Eco53kI

AGCAGCAGACCGTTGCAAACGCAGGACCTCCACTCCTCTTCTCCTCAACACCCACTTTTGCCATCGAAAAACCAGCCCAGTTATTGGGCTTGATTGGAGCTCGCTCA
TCGTCGTCTGGCAACGTTTGCGTCCTGGAGGTGAGGAGAAGAGGAGTTGTGGGTGAAAACGGTAGCTTTTTGGTCGGGTCAATAACCCGAACTAACCTCGAGCGAGT

AOX1 promoter

120 140 160 180 200

TTCCAATTCCTTCTATTAGGCTACTAACACCATGACTTTATTAGCCTGTCTATCCTGGCCCCCCTGGCGAGGTTCATGTTTGTTTATTTCCGAATGCAACAAGCTCC
AAGGTTAAGGAAGATAATCCGATGATTGTGGTACTGAAATAATCGGACAGATAGGACCGGGGGGACCGCTCCAAGTACAAACAAATAAAGGCTTACGTTGTTCGAGG

AOX1 promoter

220 240 260 280 300 320

PmeI

GCATTACACCCGAACATCACTCCAGATGAGGGCTTTCTGAGTGTGGGGTCAAATAGTTTCATGTTCCCCAAATGGCCCAAAACTGACAGTTTAAACGCTGTCTTGGA
CGTAATGTGGGCTTGTAGTGAGGTCTACTCCCGAAAGACTCACACCCCAGTTTATCAAAGTACAAGGGGTTTACCGGGTTTTGACTGTCAAATTTGCGACAGAACCT

AOX1 promoter

340 360 380 400 420

ACCTAATATGACAAAAGCGTGATCTCATCCAAGATGAACTAAGTTTGGTTCGTTGAAATGCTAACGGCCAGTTGGTCAAAAAGAAACTTCCAAAAGTCGCCATACCG
TGGATTATACTGTTTTCGCACTAGAGTAGGTTCTACTTGATTCAAACCAAGCAACTTTACGATTGCCGGTCAACCAGTTTTTCTTTGAAGGTTTTCAGCGGTATGGC

AOX1 promoter

440 460 480 500 520

BlpI

TTTGTCTTGTTTGGTATTGATTGACGAATGCTCAAAAATAATCTCATTAATGCTTAGCGCAGTCTCTCTATCGCTTCTGAACCCCGGTGCACCTGTGCCGAAACGCA
AAACAGAACAAACCATAACTAACTGCTTACGAGTTTTTATTAGAGTAATTACGAATCGCGTCAGAGAGATAGCGAAGACTTGGGGCCACGTGGACACGGCTTTGCGT

AOX1 promoter

540 560 580 600 620 640

XcmI

AATGGGGAAACACCCGCTTTTTGGATGATTATGCATTGTCTCCACATTGTATGCTTCCAAGATTCTGGTGGGAATACTGCTGATAGCCTAACGTTCATGATCAAAAT
TTACCCCTTTGTGGGCGAAAAACCTACTAATACGTAACAGAGGTGTAACATACGAAGGTTCTAAGACCACCCTTATGACGACTATCGGATTGCAAGTACTAGTTTTA

AOX1 promoter

660 680 700 720 740

TTAACTGTTCTAACCCCTACTTGACAGCAATATATAAACAGAAGGAAGCTGCCCTGTCTTAAACCTTTTTTTTTATCATCATTATTAGCTTACTTTCATAATTGCGA
AATTGACAAGATTGGGGATGAACTGTCGTTATATATTTGTCTTCCTTCGACGGGACAGAATTTGGAAAAAAAAATAGTAGTAATAATCGAATGAAAGTATTAACGCT

AOX1 promoter

760 780 800 820 840

BamHI
BstBI

CTGGTTCCAATTGACAAGCTTTTGATTTTAACGACTTTTAACGACAACTTGAGAAGATCAAAAAACAACTAATTATTCGAAGGATCCAAACGATGAGATTTCCTTCA
GACCAAGGTTAACTGTTCGAAAACTAAAATTGCTGAAAATTGCTGTTGAACTCTTCTAGTTTTTTGTTGATTAATAAGCTTCCTAGGTTTGCTACTCTAAAGGAAGT

2 4
M R F P S

AOX1 promoter

860 880 900 920 940 960

ATTTTTACTGCAGTTTTATTCGCAGCATCCTCCGCATTAGCTGCTCCAGTCAACACTACAACAGAAGATGAAACGGCACAAATTCCGGCTGAAGCTGTCATCGGTTA
TAAAAATGACGTCAAAATAAGCGTCGTAGGAGGCGTAATCGACGAGGTCAGTTGTGATGTTGTCTTCTACTTTGCCGTGTTTAAGGCCGACTTCGACAGTAGCCAAT

6 8 10 12 14 16 18 20 22 24 26 28 30 32 34 36 38 40
I F T A V L F A A S S A L A A P V N T T T E D E T A Q I P A E A V I G Y

α-factor secretion signal

980 1,000 1,020 1,040 1,060

PsiI

CTCAGATTTAGAAGGGGATTTCGATGTTGCTGTTTTGCCATTTTCCAACAGCACAAATAACGGGTTATTGTTTATAAATACTACTATTGCCAGCATTGCTGCTAAAG
GAGTCTAAATCTTCCCCTAAAGCTACAACGACAAAACGGTAAAAGGTTGTCGTGTTTATTGCCCAATAACAAATATTTATGATGATAACGGTCGTAACGACGATTTC

42 44 46 48 50 52 54 56 58 60 62 64 66 68 70 72 74 76
S D L E G D F D V A V L P F S N S T N N G L L F I N T T I A S I A A K

α-factor secretion signal

1,080 1,100 1,120 1,140 1,160

PaeR7I
XhoI
TliI
EcoRI
SnaBI

AAGAAGGGGTATCTCTCGAGAAAAGAGAGGCTGAAGCTTACGTAGAATTCATGCAGGTTCAATTGGTTGAATCTGGTGGTGCCCTTGTTCAACCTGGAGGATCACTA
TTCTTCCCCATAGAGAGCTCTTTTCTCTCCGACTTCGAATGCATCTTAAGTACGTCCAAGTTAACCAACTTAGACCACCACGGGAACAAGTTGGACCTCCTAGTGAT

78 80 82 84 86 88 90 92 94 96 98 100 102 104 106 108 110 112
E E G V S L E K R E A E A Y V E F M Q V Q L V E S G G A L V Q P G G S L

α-factor secretion signal GFP nanobody

1,180 1,200 1,220 1,240 1,260 1,280

BspEI HpaI

AGATTATCCTGTGCTGCCTCCGGATTTCCTGTTAACAGGTACTCCATGAGATGGTATAGACAGGCTCCAGGTAAAGAAAGAGAGTGGGTCGCTGGTATGTCATCTGC
TCTAATAGGACACGACGGAGGCCTAAAGGACAATTGTCCATGAGGTACTCTACCATATCTGTCCGAGGTCCATTTCTTTCTCTCACCCAGCGACCATACAGTAGACG

114 116 118 120 122 124 126 128 130 132 134 136 138 140 142 144 146 148

R L S C A A S G F P V N R Y S M R W Y R Q A P G K E R E W V A G M S S A

GFP nanobody

1,300 1,320 1,340 1,360 1,380

EcoRI

TGGAGATAGATCCTCATACGAGGATTCTGTCAAAGGAAGGTTTACTATTAGTCGTGACGACGCACGTAATACCGTTTATTTGCAAATGAATTCTCTGAAACCAGAAG
ACCTCTATCTAGGAGTATGCTCCTAAGACAGTTTCCTTCCAAATGATAATCAGCACTGCTGCGTGCATTATGGCAAATAAACGTTTACTTAAGAGACTTTGGTCTTC

150 152 154 156 158 160 162 164 166 168 170 172 174 176 178 180 182

G D R S S Y E D S V K G R F T I S R D D A R N T V Y L Q M N S L K P E

GFP nanobody

1,400 1,420 1,440 1,460 1,480

Tth111I
PflFI
ScaI BaeI

ACACTGCAGTATATTACTGCAACGTCAACGTAGGTTTCGAGTACTGGGGCCAAGGTACACAGGTGACGGTGTCTAGTGGCGGTAGTGACTACAAAGATGACGACGAT
TGTGACGTCATATAATGACGTTGCAGTTGCATCCAAAGCTCATGACCCCGGTTCCATGTGTCCACTGCCACAGATCACCGCCATCACTGATGTTTCTACTGCTGCTA

184 186 188 190 192 194 196 198 200 202 204 206 208 210 212 214 216 218

D T A V Y Y C N V N V G F E Y W G Q G T Q V T V S S G G S D Y K D D D D

GFP nanobody FLAG

1,500 1,520 1,540 1,560 1,580 1,600

NotI
EagI
XbaI

AAATTTGTCGGTGCCCGTCTAGACTACAAGGATGATGATGACAAGTAAGCGGCCGCGAATTAATTCGCCTTAGACATGACTGTTCCTCAGTTCAAGTTGGGCACTTA
TTTAAACAGCCACGGGCAGATCTGATGTTCCTACTACTACTGTTCATTCGCCGGCGCTTAATTAAGCGGAATCTGTACTGACAAGGAGTCAAGTTCAACCCGTGAAT

220 222 224 226 228 230 232 234

K F V G A R L D Y K D D D D K *

barcode2 FLAG

1,620 1,640 1,660 1,680 1,700

AgeI

CGAGAAGACCGGTCTTGCTAGATTCTAATCAAGAGGATGTCAGAATGCCATTTGCCTGAGAGATGCAGGCTTCATTTTTGATACTTTTTTATTTGTAACCTATATAG
GCTCTTCTGGCCAGAACGATCTAAGATTAGTTCTCCTACAGTCTTACGGTAAACGGACTCTCTACGTCCGAAGTAAAAACTATGAAAAAATAAACATTGGATATATC

AOX1 terminator

1,720 1,740 1,760 1,780 1,800

BsiWI PvuII
TATAGGATTTTTTTGTCATTTTGTTTCTTCTCGTACGAGCTTGCTCCTGATCAGCCTATCTCGCAGCTGATGAATATCTTGTGGTAGGGGTTTGGGAAAATCATTC
ATATCCTAAAAAAACAGTAAAACAAAGAAGAGCATGCTCGAACGAGGACTAGTCGGATAGAGCGTCGACTACTTATAGAACACCATCCCCAAACCCTTTTAGTAAG
AOX1 terminator
1,820 1,840 1,860 1,880 1,900 1,920

BcgI
GAGTTTGATGTTTTTCTTGGTATTTCCCACTCCTCTTCAGAGTACAGAAGATTAAGTGAGAAGTTCGTTTGTGCAAGCTTATCGATAAGCTTTAATGCGGTAGTTTA
CTCAAACTACAAAAAGAACCATAAAGGGTGAGGAGAAGTCTCATGTCTTCTAATTCACTCTTCAAGCAAACACGTTCGAATAGCTATTCGAAATTACGCCATCAAAT
AOX1 terminator
1,940 1,960 1,980 2,000 2,020

TCACAGTTAAATTGCTAACGCAGTCAGGCACCGTGTATGAAATCTAACAATGCGCTCATCGTCATCCTCGGCACCGTCACCCTGGATGCTGTAGGCATAGGCTTGGT
AGTGTCAATTTAACGATTGCGTCAGTCCGTGGCACATACTTTAGATTGTTACGCGAGTAGCAGTAGGAGCCGTGGCAGTGGGACCTACGACATCCGTATCCGAACCA
2,040 2,060 2,080 2,100 2,120 2,140

AfeI
BtgZI BmtI FspI
EcoRV NheI FspAI
TATGCCGGTACTGCCGGGCCTCTTGCGGGATATCGTCCATTCCGACAGCATCGCCAGTCACTATGGCGTGCTGCTAGCGCTATATGCGTTGATGCAATTTCTATGCG
ATACGGCCATGACGGCCCGGAGAACGCCCTATAGCAGGTAAGGCTGTCGTAGCGGTCAGTGATACCGCACGACGATCGCGATATACGCAACTACGTTAAAGATACGC
2,160 2,180 2,200 2,220 2,240

CACCCGTTCTCGGAGCACTGTCCGACCGCTTTGGCCGCCGCCCAGTCCTGCTCGCTTCGCTACTTGGAGCCACTATCGACTACGCGATCATGGCGACCACACCCGTC
GTGGGCAAGAGCCTCGTGACAGGCTGGCGAAACCGGCGGCGGGTCAGGACGAGCGAAGCGATGAACCTCGGTGATAGCTGATGCGCTAGTACCGCTGGTGTGGGCAG
2,260 2,280 2,300 2,320 2,340

XbaI
CTGTGGATCTATCGAATCTAAATGTAAGTTAAAATCTCTAAATAATTAAATAAGTCCCAGTTTCTCCATACGAACCTTAACAGCATTGCGGTGAGCATCTAGACCTT
GACACCTAGATAGCTTAGATTTACATTCAATTTTAGAGATTTATTAATTTATTCAGGGTCAAAGAGGTATGCTTGGAATTGTCGTAACGCCACTCGTAGATCTGGAA
2,360 2,380 2,400 2,420 2,440 2,460

MscI Bsu36I HpaI
CAACAGCAGCCAGATCCATCACTGCTTGGCCAATATGTTTCAGTCCCTCAGGAGTTACGTCTTGTGAAGTGATGAACTTCTGGAAGGTTGCAGTGTTAACTCCGCTG
GTTGTCGTCGGTCTAGGTAGTGACGAACCGGTTATACAAAGTCAGGGAGTCCTCAATGCAGAACACTTCACTACTTGAAGACCTTCCAACGTCACAATTGAGGCGAC
2,480 2,500 2,520 2,540 2,560

KpnI
BsiWI Acc65I
TATTGACGGGCATATCCGTACGTTGGCAAAGTGTGGTTGGTACCGGAGGAGTAATCTCCACAACTCTCTGGAGAGTAGGCACCAACAAACACAGATCCAGCGTGTTG
ATAACTGCCCGTATAGGCATGCAACCGTTTCACACCAACCATGGCCTCCTCATTAGAGGTGTTGAGAGACCTCTCATCCGTGGTTGTTTGTGTCTAGGTCGCACAAC
2,580 2,600 2,620 2,640 2,660

AloI

TACTTGATCAACATAAGAAGAAGCATTCTCGATTTGCAGGATCAAGTGTTCAGGAGCGTACTGATTGGACATTTCCAAAGCCTGCTCGTAGGTTGCAACCGATAGGG
ATGAACTAGTTGTATTCTTCTTCGTAAGAGCTAAACGTCCTAGTTCACAAGTCCTCGCATGACTAACCTGTAAAGGTTTCGGACGAGCATCCAACGTTGGCTATCCC

2,680 2,700 2,720 2,740 2,760 2,780

BsgI

TTGTAGAGTGTGCAATACACTTGCGTACAATTTCAACCCTTGGCAACTGCACAGCTTGGTTGTGAACAGCATCTTCAATTCTGGCAAGCTCCTTGTCTGTCATATCG
AACATCTCACACGTTATGTGAACGCATGTTAAAGTTGGGAACCGTTGACGTGTCGAACCAACACTTGTCGTAGAAGTTAAGACCGTTCGAGGAACAGACAGTATAGC

2,800 2,820 2,840 2,860 2,880

ACAGCCAACAGAATCACCTGGGAATCAATACCATGTTCAGCTTGAGACAGAAGGTCTGAGGCAACGAAATCTGGATCAGCGTATTTATCAGCAATAACTAGAACTTC
TGTCGGTTGTCTTAGTGGACCCTTAGTTATGGTACAAGTCGAACTCTGTCTTCCAGACTCCGTTGCTTTAGACCTAGTCGCATAAATAGTCGTTATTGATCTTGAAG

2,900 2,920 2,940 2,960 2,980

PfoI

AGAAGGCCCAGCAGGCATGTCAATACTACACAGGGCTGATGTGTCATTTTGAACCATCATCTTGGCAGCAGTAACGAACTGGTTTCCTGGACCAAATATTTTGTCAC
TCTTCCGGGTCGTCCGTACAGTTATGATGTGTCCCGACTACACAGTAAAACTTGGTAGTAGAACCGTCGTCATTGCTTGACCAAAGGACCTGGTTTATAAAACAGTG

3,000 3,020 3,040 3,060 3,080 3,100

BmtI
NheI

ACTTAGGAACAGTTTCTGTTCCGTAAGCCATAGCAGCTACTGCCTGGGCGCCTCCTGCTAGCACGATACACTTAGCACCAACCTTGTGGGCAACGTAGATGACTTCT
TGAATCCTTGTCAAAGACAAGGCATTCGGTATCGTCGATGACGGACCCGCGGAGGACGATCGTGCTATGTGAATCGTGGTTGGAACACCCGTTGCATCTACTGAAGA

3,120 3,140 3,160 3,180 3,200

KpnI
Acc65I

GGGGTAAGGGTACCATCCTTCTTAGGTGGAGATGCAAAAACAATTTCTTTGCAACCAGCAACTTTGGCAGGAACACCCAGCATCAGGGAAGTGGAAGGCAGAATTGC
CCCCATTCCCATGGTAGGAAGAATCCACCTCTACGTTTTTGTTAAAGAAACGTTGGTCGTTGAAACCGTCCTTGTGGGTCGTAGTCCCTTCACCTTCCGTCTTAACG

3,220 3,240 3,260 3,280 3,300

GGTTCCACCAGGAATATAGAGGCCAACTTTCTCAATAGGTCTTGCAAAACGAGAGCAGACTACACCAGGGCAAGTCTCAACTTGCAACGTCTCCGTTAGTTGAGCTT
CCAAGGTGGTCCTTATATCTCCGGTTGAAAGAGTTATCCAGAACGTTTTGCTCTCGTCTGATGTGGTCCCGTTCAGAGTTGAACGTTGCAGAGGCAATCAACTCGAA

3,320 3,340 3,360 3,380 3,400 3,420

CATGGAATTTCCTGACGTTATCTATAGAGAGATCAATGGCTCTCTTAACGTTATCTGGCAATTGCATAAGTTCCTCTGGGAAAGGAGCTTCTAACACAGGTGTCTTC
GTACCTTAAAGGACTGCAATAGATATCTCTCTAGTTACCGAGAGAATTGCAATAGACCGTTAACGTATTCAAGGAGACCCTTTCCTCGAAGATTGTGTCCACAGAAG

3,440 3,460 3,480 3,500 3,520

SalI

AAAGCGACTCCATCAAACTTGGCAGTTAGTTCTAAAAGGGCTTTGTCACCATTTTGACGAACATTGTCGACAATTGGTTTGACTAATTCCATAATCTGTTCCGTTTT
TTTCGCTGAGGTAGTTTGAACCGTCAATCAAGATTTTCCCGAAACAGTGGTAAAACTGCTTGTAACAGCTGTTAACCAAACTGATTAAGGTATTAGACAAGGCAAAA

3,540 3,560 3,580 3,600 3,620

StuI
CTGGATAGGACGACGAAGGGCATCTTCAATTTCTTGTGAGGAGGCCTTAGAAACGTCAATTTTGCACAATTCAATACGACCTTCAGAAGGGACTTCTTTAGGTTTGG
GACCTATCCTGCTGCTTCCCGTAGAAGTTAAAGAACACTCCTCCGGAATCTTTGCAGTTAAAACGTGTTAAGTTATGCTGGAAGTCTTCCCTGAAGAAATCCAAACC
3,640 3,660 3,680 3,700 3,720 3,740

ATTCTTCTTTAGGTTGTTCCTTGGTGTATCCTGGCTTGGCATCTCCTTTCCTTCTAGTGACCTTTAGGGACTTCATATCCAGGTTTCTCTCCACCTCGTCCAACGTC
TAAGAAGAAATCCAACAAGGAACCACATAGGACCGAACCGTAGAGGAAAGGAAGATCACTGGAAATCCCTGAAGTATAGGTCCAAAGAGAGGTGGAGCAGGTTGCAG
3,760 3,780 3,800 3,820 3,840

ACACCGTACTTGGCACATCTAACTAATGCAAAATAAAATAAGTCAGCACATTCCCAGGCTATATCTTCCTTGGATTTAGCTTCTGCAAGTTCATCAGCTTCCTCCCT
TGTGGCATGAACCGTGTAGATTGATTACGTTTTATTTTATTCAGTCGTGTAAGGGTCCGATATAGAAGGAACCTAAATCGAAGACGTTCAAGTAGTCGAAGGAGGGA
3,860 3,880 3,900 3,920 3,940

DraIII NcoI
AATTTTAGCGTTCAACAAAACTTCGTCGTCAAATAACCGTTTGGTATAAGAACCTTCTGGAGCATTGCTCTTACGATCCCACAAGGTGGCTTCCATGGCTCTAAGAC
TTAAAATCGCAAGTTGTTTTGAAGCAGCAGTTTATTGGCAAACCATATTCTTGGAAGACCTCGTAACGAGAATGCTAGGGTGTTCCACCGAAGGTACCGAGATTCTG
3,960 3,980 4,000 4,020 4,040 4,060

MscI
CCTTTGATTGGCCAAAACAGGAAGTGCGTTCCAAGTGACAGAAACCAACACCTGTTTGTTCAACCACAAATTTCAAGCAGTCTCCATCACAATCCAATTCGATACCC
GGAAACTAACCGGTTTTGTCCTTCACGCAAGGTTCACTGTCTTTGGTTGTGGACAAACAAGTTGGTGTTTAAAGTTCGTCAGAGGTAGTGTTAGGTTAAGCTATGGG
4,080 4,100 4,120 4,140 4,160

BspEI
AGCAACTTTTGAGTTGCTCCAGATGTAGCACCTTTATACCACAAACCGTGACGACGAGATTGGTAGACTCCAGTTTGTGTCCTTATAGCCTCCGGAATAGACTTTTT
TCGTTGAAAACTCAACGAGGTCTACATCGTGGAAATATGGTGTTTGGCACTGCTGCTCTAACCATCTGAGGTCAAACACAGGAATATCGGAGGCCTTATCTGAAAAA
4,180 4,200 4,220 4,240 4,260 4,280

GGACGAGTACACCAGGCCCAACGAGTAATTAGAAGAGTCAGCCACCAAAGTAGTGAATAGACCATCGGGGCGGTCAGTAGTCAAAGACGCCAACAAAATTTCACTGA
CCTGCTCATGTGGTCCGGGTTGCTCATTAATCTTCTCAGTCGGTGGTTTCATCACTTATCTGGTAGCCCCGCCAGTCATCAGTTTCTGCGGTTGTTTTAAAGTGACT
4,300 4,320 4,340 4,360 4,380

CAGGGAACTTTTTGACATCTTCAGAAAGTTCGTATTCAGTAGTCAATTGCCGAGCATCAATAATGGGGATTATACCAGAAGCAACAGTGGAAGTCACATCTACCAAC
GTCCCTTGAAAAACTGTAGAAGTCTTTCAAGCATAAGTCATCAGTTAACGGCTCGTAGTTATTACCCCTAATATGGTCTTCGTTGTCACCTTCAGTGTAGATGGTTG
4,400 4,420 4,440 4,460 4,480

TTTGCGGTCTCAGAAAAAGCATAAACAGTTCTACTACCGCCATTAGTGAAACTTTTCAAATCGCCCAGTGGAGAAGAAAAAGGCACAGCGATACTAGCATTAGCGGG
AAACGCCAGAGTCTTTTTCGTATTTGTCAAGATGATGGCGGTAATCACTTTGAAAAGTTTAGCGGGTCACCTCTTCTTTTTCCGTGTCGCTATGATCGTAATCGCCC
4,500 4,520 4,540 4,560 4,580 4,600

PpuMI

CAAGGATGCAACTTTATCAACCAGGGTCCTATAGATAACCCTAGCGCCTGGGATCATCCTTTGGACAACTCTTTCTGCCAAATCTAGGTCCAAAATCACTTCATTGA
GTTCCTACGTTGAAATAGTTGGTCCCAGGATATCTATTGGGATCGCGGACCCTAGTAGGAAACCTGTTGAGAAAGACGGTTTAGATCCAGGTTTTAGTGAAGTAACT

4,620 4,640 4,660 4,680 4,700

BsrGI

TACCATTATTGTACAACTTGAGCAAGTTGTCGATCAGCTCCTCAAATTGGTCCTCTGTAACGGATGACTCAACTTGCACATTAACTTGAAGCTCAGTCGATTGAGTG
ATGGTAATAACATGTTGAACTCGTTCAACAGCTAGTCGAGGAGTTTAACCAGGAGACATTGCCTACTGAGTTGAACGTGTAATTGAACTTCGAGTCAGCTAACTCAC

4,720 4,740 4,760 4,780 4,800

PvuII BsgI

AACTTGATCAGGTTGTGCAGCTGGTCAGCAGCATAGGGAAACACGGCTTTTCCTACCAAACTCAAGGAATTATCAAACTCTGCAACACTTGCGTATGCAGGTAGCAA
TTGAACTAGTCCAACACGTCGACCAGTCGTCGTATCCCTTTGTGCCGAAAAGGATGGTTTGAGTTCCTTAATAGTTTGAGACGTTGTGAACGCATACGTCCATCGTT

4,820 4,840 4,860 4,880 4,900 4,920

GGGAAATGTCATACTTGAAGTCGGACAGTGAGTGTAGTCTTGAGAAATTCTGAAGCCGTATTTTTATTATCAGTGAGTCAGTCATCAGGAGATCCTCTACGCCGGAC
CCCTTTACAGTATGAACTTCAGCCTGTCACTCACATCAGAACTCTTTAAGACTTCGGCATAAAAATAATAGTCACTCAGTCAGTAGTCCTCTAGGAGATGCGGCCTG

4,940 4,960 4,980 5,000 5,020

Bpu10I
SbfI BbvCI StuI

GCATCGTGGCCGACCTGCAGGGGGGGGGGGGGGCGCTGAGGTCTGCCTCGTGAAGAAGGTGTTGCTGACTCATACCAGGCCTGAATCGCCCCATCATCCAGCCAGAAA
CGTAGCACCGGCTGGACGTCCCCCCCCCCCCCCGCGACTCCAGACGGAGCACTTCTTCCACAACGACTGAGTATGGTCCGGACTTAGCGGGGTAGTAGGTCGGTCTTT

5,040 5,060 5,080 5,100 5,120

GTGAGGGAGCCACGGTTGATGAGAGCTTTGTTGTAGGTGGACCAGTTGGTGATTTTGAACTTTTGCTTTGCCACGGAACGGTCTGCGTTGTCGGGAAGATGCGTGAT
CACTCCCTCGGTGCCAACTACTCTCGAAACAACATCCACCTGGTCAACCACTAAAACTTGAAAACGAAACGGTGCCTTGCCAGACGCAACAGCCCTTCTACGCACTA

5,140 5,160 5,180 5,200 5,220 5,240

CTGATCCTTCAACTCAGCAAAAGTTCGATTTATTCAACAAAGCCGCCGTCCCGTCAAGTCAGCGTAATGCTCTGCCAGTGTTACAACCAATTAACCAATTCTGATTA
GACTAGGAAGTTGAGTCGTTTTCAAGCTAAATAAGTTGTTTCGGCGGCAGGGCAGTTCAGTCGCATTACGAGACGGTCACAATGTTGGTTAATTGGTTAAGACTAAT

5,260 5,280 5,300 5,320 5,340

GAAAAACTCATCGAGCATCAAATGAAACTGCAATTTATTCATATCAGGATTATCAATACCATATTTTTGAAAAAGCCGTTTCTGTAATGAAGGAGAAAACTCACCGA
CTTTTTGAGTAGCTCGTAGTTTACTTTGACGTTAAATAAGTATAGTCCTAATAGTTATGGTATAAAAACTTTTTCGGCAAAGACATTACTTCCTCTTTTGAGTGGCT

5,360 5,380 5,400 5,420 5,440

GGCAGTTCCATAGGATGGCAAGATCCTGGTATCGGTCTGCGATTCCGACTCGTCCAACATCAATACAACCTATTAATTTCCCCTCGTCAAAAATAAGGTTATCAAGT
CCGTCAAGGTATCCTACCGTTCTAGGACCATAGCCAGACGCTAAGGCTGAGCAGGTTGTAGTTATGTTGGATAATTAAAGGGGAGCAGTTTTTATTCCAATAGTTCA

5,460 5,480 5,500 5,520 5,540 5,560

GAGAAATCACCATGAGTGACGACTGAATCCGGTGAGAATGGCAAAAGCTTATGCATTTCTTTCCAGACTTGTTCAACAGGCCAGCCATTACGCTCGTCATCAAAATC
CTCTTTAGTGGTACTCACTGCTGACTTAGGCCACTCTTACCGTTTTCGAATACGTAAAGAAAGGTCTGAACAAGTTGTCCGGTCGGTAATGCGAGCAGTAGTTTTAG

5,580 5,600 5,620 5,640 5,660

Bpu10I
AjuI
AsiSI
PvuI

ACTCGCATCAACCAAACCGTTATTCATTCGTGATTGCGCCTGAGCGAGACGAAATACGCGATCGCTGTTAAAAGGACAATTACAAACAGGAATCGAATGCAACCGGC
TGAGCGTAGTTGGTTTGGCAATAAGTAAGCACTAACGCGGACTCGCTCTGCTTTATGCGCTAGCGACAATTTTCCTGTTAATGTTTGTCCTTAGCTTACGTTGGCCG

5,680 5,700 5,720 5,740 5,760

SmaI
TspMI
XmaI

GCAGGAACACTGCCAGCGCATCAACAATATTTTCACCTGAATCAGGATATTCTTCTAATACCTGGAATGCTGTTTTCCCGGGGATCGCAGTGGTGAGTAACCATGCA
CGTCCTTGTGACGGTCGCGTAGTTGTTATAAAAGTGGACTTAGTCCTATAAGAAGATTATGGACCTTACGACAAAAGGGCCCCTAGCGTCACCACTCATTGGTACGT

5,780 5,800 5,820 5,840 5,860 5,880

TCATCAGGAGTACGGATAAAATGCTTGATGGTCGGAAGAGGCATAAATTCCGTCAGCCAGTTTAGTCTGACCATCTCATCTGTAACATCATTGGCAACGCTACCTTT
AGTAGTCCTCATGCCTATTTTACGAACTACCAGCCTTCTCCGTATTTAAGGCAGTCGGTCAAATCAGACTGGTAGAGTAGACATTGTAGTAACCGTTGCGATGGAAA

5,900 5,920 5,940 5,960 5,980

NruI

GCCATGTTTCAGAAACAACTCTGGCGCATCGGGCTTCCCATACAATCGATAGATTGTCGCACCTGATTGCCCGACATTATCGCGAGCCCATTTATACCCATATAAAT
CGGTACAAAGTCTTTGTTGAGACCGCGTAGCCCGAAGGGTATGTTAGCTATCTAACAGCGTGGACTAACGGGCTGTAATAGCGCTCGGGTAAATATGGGTATATTTA

6,000 6,020 6,040 6,060 6,080

PaeR7I
PspXI
XhoI
TliI

CAGCATCCATGTTGGAATTTAATCGCGGCCTCGAGCAAGACGTTTCCCGTTGAATATGGCTCATAACACCCCTTGTATTACTGTTTATGTAAGCAGACAGTTTTATT
GTCGTAGGTACAACCTTAAATTAGCGCCGGAGCTCGTTCTGCAAAGGGCAACTTATACCGAGTATTGTGGGGAACATAATGACAAATACATTCGTCTGTCAAAATAA

6,100 6,120 6,140 6,160 6,180 6,200

DraIII SbfI SgrAI

GTTCATGATGATATATTTTTATCTTGTGCAATGTAACATCAGAGATTTTGAGACACAACGTGGCTTTCCCCCCCCCCCCTGCAGGTCGGCATCACCGGCGCCACAGG
CAAGTACTACTATATAAAAATAGAACACGTTACATTGTAGTCTCTAAAACTCTGTGTTGCACCGAAAGGGGGGGGGGGGACGTCCAGCCGTAGTGGCCGCGGTGTCC

6,220 6,240 6,260 6,280 6,300

AfeI

TGCGGTTGCTGGCGCCTATATCGCCGACATCACCGATGGGGAAGATCGGGCTCGCCACTTCGGGCTCATGAGCGCTTGTTTCGGCGTGGGTATGGTGGCAGGCCCCG
ACGCCAACGACCGCGGATATAGCGGCTGTAGTGGCTACCCCTTCTAGCCCGAGCGGTGAAGCCCGAGTACTCGCGAACAAAGCCGCACCCATACCACCGTCCGGGGC

6,320 6,340 6,360 6,380 6,400 6,420

SphI

TGGCCGGGGGACTGTTGGGCGCCATCTCCTTGCATGCACCATTCCTTGCGGCGGCGGTGCTCAACGGCCTCAACCTACTACTGGGCTGCTTCCTAATGCAGGAGTCG
ACCGGCCCCCTGACAACCCGCGGTAGAGGAACGTACGTGGTAAGGAACGCCGCCGCCACGAGTTGCCGGAGTTGGATGATGACCCGACGAAGGATTACGTCCTCAGC

6,440 6,460 6,480 6,500 6,520

CATAAGGGAGAGCGTCGAGTATCTATGATTGGAAGTATGGGAATGGTGATACCCGCATTCTTCAGTGTCTTGAGGTCTCCTATCAGATTATGCCCAACTAAAGCAAC
GTATTCCCTCTCGCAGCTCATAGATACTAACCTTCATACCCTTACCACTATGGGCGTAAGAAGTCACAGAACTCCAGAGGATAGTCTAATACGGGTTGATTTCGTTG

6,540 6,560 6,580 6,600 6,620

CGGAGGAGGAGATTTCATGGTAAATTTCTCTGACTTTTGGTCATCAGTAGACTCGAACTGTGAGACTATCTCGGTTATGACAGCAGAAATGTCCTTCTTGGAGACAG
GCCTCCTCCTCTAAAGTACCATTTAAAGAGACTGAAAACCAGTAGTCATCTGAGCTTGACACTCTGATAGAGCCAATACTGTCGTCTTTACAGGAAGAACCTCTGTC

6,640 6,660 6,680 6,700 6,720 6,740

BstBI

TAAATGAAGTCCCACCAATAAAGAAATCCTTGTTATCAGGAACAAACTTCTTGTTTCGAACTTTTTCGGTGCCTTGAACTATAAAATGTAGAGTGGATATGTCGGGT
ATTTACTTCAGGGTGGTTATTTCTTTAGGAACAATAGTCCTTGTTTGAAGAACAAAGCTTGAAAAAGCCACGGAACTTGATATTTTACATCTCACCTATACAGCCCA

6,760 6,780 6,800 6,820 6,840

AGGAATGGAGCGGGCAAATGCTTACCTTCTGGACCTTCAAGAGGTATGTAGGGTTTGTAGATACTGATGCCAACTTCAGTGACAACGTTGCTATTTCGTTCAAACCA
TCCTTACCTCGCCCGTTTACGAATGGAAGACCTGGAAGTTCTCCATACATCCCAAACATCTATGACTACGGTTGAAGTCACTGTTGCAACGATAAAGCAAGTTTGGT

6,860 6,880 6,900 6,920 6,940

TTCCGAATCCAGAGAAATCAAAGTTGTTTGTCTACTATTGATCCAAGCCAGTGCGGTCTTGAAACTGACAATAGTGTGCTCGTGTTTTGAGGTCATCTTTGTATGAA
AAGGCTTAGGTCTCTTTAGTTTCAACAAACAGATGATAACTAGGTTCGGTCACGCCAGAACTTTGACTGTTATCACACGAGCACAAAACTCCAGTAGAAACATACTT

6,960 6,980 7,000 7,020 7,040 7,060

TAAATCTAGTCTTTGATCTAAATAATCTTGACGAGCCAAGGCGATAAATACCCAAATCTAAAACTCTTTTAAAACGTTAAAAGGACAAGTATGTCTGCCTGTATTAA
ATTTAGATCAGAAACTAGATTTATTAGAACTGCTCGGTTCCGCTATTTATGGGTTTAGATTTTGAGAAAATTTTGCAATTTTCCTGTTCATACAGACGGACATAATT

7,080 7,100 7,120 7,140 7,160

EcoRV

ACCCCAAATCAGCTCGTAGTCTGATCCTCATCAACTTGAGGGGCACTATCTTGTTTTAGAGAAATTTGCGGAGATGCGATATCGAGAAAAAGGTACGCTGATTTTAA
TGGGGTTTAGTCGAGCATCAGACTAGGAGTAGTTGAACTCCCCGTGATAGAACAAAATCTCTTTAAACGCCTCTACGCTATAGCTCTTTTTCCATGCGACTAAAATT

7,180 7,200 7,220 7,240 7,260

BglII PfoI

ACGTGAAATTTATCTCAAGATCTCTGCCTCGCGCGTTTCGGTGATGACGGTGAAAACCTCTGACACATGCAGCTCCCGGAGACGGTCACAGCTTGTCTGTAAGCGGA
TGCACTTTAAATAGAGTTCTAGAGACGGAGCGCGCAAAGCCACTACTGCCACTTTTGGAGACTGTGTACGTCGAGGGCCTCTGCCAGTGTCGAACAGACATTCGCCT

7,280 7,300 7,320 7,340 7,360 7,380

Tth111I
PflFI BstZ17I

TGCCGGGAGCAGACAAGCCCGTCAGGGCGCGTCAGCGGGTGTTGGCGGGTGTCGGGGCGCAGCCATGACCCAGTCACGTAGCGATAGCGGAGTGTATACTGGCTTAA
ACGGCCCTCGTCTGTTCGGGCAGTCCCGCGCAGTCGCCCACAACCGCCCACAGCCCCGCGTCGGTACTGGGTCAGTGCATCGCTATCGCCTCACATATGACCGAATT

7,400 7,420 7,440 7,460 7,480

NdeI BspQI SapI

CTATGCGGCATCAGAGCAGATTGTACTGAGAGTGCACCATATGCGGTGTGAAATACCGCACAGATGCGTAAGGAGAAAATACCGCATCAGGCGCTCTTCCGCTTCCT
GATACGCCGTAGTCTCGTCTAACATGACTCTCACGTGGTATACGCCACACTTTATGGCGTGTCTACGCATTCCTCTTTTATGGCGTAGTCCGCGAGAAGGCGAAGGA

7,500 7,520 7,540 7,560 7,580

CGCTCACTGACTCGCTGCGCTCGGTCGTTCGGCTGCGGCGAGCGGTATCAGCTCACTCAAAGGCGGTAATACGGTTATCCACAGAATCAGGGGATAACGCAGGAAAG
GCGAGTGACTGAGCGACGCGAGCCAGCAAGCCGACGCCGCTCGCCATAGTCGAGTGAGTTTCCGCCATTATGCCAATAGGTGTCTTAGTCCCCTATTGCGTCCTTTC

7,600 7,620 7,640 7,660 7,680 7,700

AflIII PciI

AACATGTGAGCAAAAGGCCAGCAAAAGGCCAGGAACCGTAAAAAGGCCGCGTTGCTGGCGTTTTTCCATAGGCTCCGCCCCCCTGACGAGCATCACAAAAATCGACG
TTGTACACTCGTTTTCCGGTCGTTTTCCGGTCCTTGGCATTTTTCCGGCGCAACGACCGCAAAAAGGTATCCGAGGCGGGGGGACTGCTCGTAGTGTTTTTAGCTGC

ColE1 origin

7,720 7,740 7,760 7,780 7,800

CTCAAGTCAGAGGTGGCGAAACCCGACAGGACTATAAAGATACCAGGCGTTTCCCCCTGGAAGCTCCCTCGTGCGCTCTCCTGTTCCGACCCTGCCGCTTACCGGAT
GAGTTCAGTCTCCACCGCTTTGGGCTGTCCTGATATTTCTATGGTCCGCAAAGGGGGACCTTCGAGGGAGCACGCGAGAGGACAAGGCTGGGACGGCGAATGGCCTA

ColE1 origin

7,820 7,840 7,860 7,880 7,900

ACCTGTCCGCCTTTCTCCCTTCGGGAAGCGTGGCGCTTTCTCAATGCTCACGCTGTAGGTATCTCAGTTCGGTGTAGGTCGTTCGCTCCAAGCTGGGCTGTGTGCAC
TGGACAGGCGGAAAGAGGGAAGCCCTTCGCACCGCGAAAGAGTTACGAGTGCGACATCCATAGAGTCAAGCCACATCCAGCAAGCGAGGTTCGACCCGACACACGTG

ColE1 origin

7,920 7,940 7,960 7,980 8,000 8,020

GAACCCCCCGTTCAGCCCGACCGCTGCGCCTTATCCGGTAACTATCGTCTTGAGTCCAACCCGGTAAGACACGACTTATCGCCACTGGCAGCAGCCACTGGTAACAG
CTTGGGGGGCAAGTCGGGCTGGCGACGCGGAATAGGCCATTGATAGCAGAACTCAGGTTGGGCCATTCTGTGCTGAATAGCGGTGACCGTCGTCGGTGACCATTGTC

ColE1 origin

8,040 8,060 8,080 8,100 8,120

GATTAGCAGAGCGAGGTATGTAGGCGGTGCTACAGAGTTCTTGAAGTGGTGGCCTAACTACGGCTACACTAGAAGGACAGTATTTGGTATCTGCGCTCTGCTGAAGC
CTAATCGTCTCGCTCCATACATCCGCCACGATGTCTCAAGAACTTCACCACCGGATTGATGCCGATGTGATCTTCCTGTCATAAACCATAGACGCGAGACGACTTCG

ColE1 origin

8,140 8,160 8,180 8,200 8,220

CAGTTACCTTCGGAAAAAGAGTTGGTAGCTCTTGATCCGGCAAACAAACCACCGCTGGTAGCGGTGGTTTTTTTGTTTGCAAGCAGCAGATTACGCGCAGAAAAAAA
GTCAATGGAAGCCTTTTTCTCAACCATCGAGAACTAGGCCGTTTGTTTGGTGGCGACCATCGCCACCAAAAAAACAAACGTTCGTCGTCTAATGCGCGTCTTTTTTT

ColE1 origin

8,240 8,260 8,280 8,300 8,320 8,340

GGATCTCAAGAAGATCCTTTGATCTTTTCTACGGGGTCTGACGCTCAGTGGAACGAAAACTCACGTTAAGGGATTTTGGTCATGAGATTATCAAAAAGGATCTTCAC
CCTAGAGTTCTTCTAGGAAACTAGAAAAGATGCCCCAGACTGCGAGTCACCTTGCTTTTGAGTGCAATTCCCTAAAACCAGTACTCTAATAGTTTTTCCTAGAAGTG

ColE1 origin

8,360 8,380 8,400 8,420 8,440

CTAGATCCTTTTAAATTAAAAATGAAGTTTTAAATCAATCTAAAGTATATATGAGTAAACTTGGTCTGACAGTTACCAATGCTTAATCAGTGAGGCACCTATCTCAG
GATCTAGGAAAATTTAATTTTTACTTCAAAATTTAGTTAGATTTCATATATACTCATTTGAACCAGACTGTCAATGGTTACGAATTAGTCACTCCGTGGATAGAGTC

AmpR

8,460 8,480 8,500 8,520 8,540 8,560

CGATCTGTCTATTTCGTTCATCCATAGTTGCCTGACTCCCCGTCGTGTAGATAACTACGATACGGGAGGGCTTACCATCTGGCCCCAGTGCTGCAATGATACCGCGA
GCTAGACAGATAAAGCAAGTAGGTATCAACGGACTGAGGGGCAGCACATCTATTGATGCTATGCCCTCCCGAATGGTAGACCGGGGTCACGACGTTACTATGGCGCT

AmpR

8,580 8,600 8,620 8,640 8,660

GACCCACGCTCACCGGCTCCAGATTTATCAGCAATAAACCAGCCAGCCGGAAGGGCCGAGCGCAGAAGTGGTCCTGCAACTTTATCCGCCTCCATCCAGTCTATTAA
CTGGGTGCGAGTGGCCGAGGTCTAAATAGTCGTTATTTGGTCGGTCGGCCTTCCCGGCTCGCGTCTTCACCAGGACGTTGAAATAGGCGGAGGTAGGTCAGATAATT

AmpR

8,680 8,700 8,720 8,740 8,760

FspI

TTGTTGCCGGGAAGCTAGAGTAAGTAGTTCGCCAGTTAATAGTTTGCGCAACGTTGTTGCCATTGCTGCAGGCATCGTGGTGTCACGCTCGTCGTTTGGTATGGCTT
AACAACGGCCCTTCGATCTCATTCATCAAGCGGTCAATTATCAAACGCGTTGCAACAACGGTAACGACGTCCGTAGCACCACAGTGCGAGCAGCAAACCATACCGAA

AmpR

8,780 8,800 8,820 8,840 8,860 8,880

PvuI

CATTCAGCTCCGGTTCCCAACGATCAAGGCGAGTTACATGATCCCCCATGTTGTGCAAAAAAGCGGTTAGCTCCTTCGGTCCTCCGATCGTTGTCAGAAGTAAGTTG
GTAAGTCGAGGCCAAGGGTTGCTAGTTCCGCTCAATGTACTAGGGGGTACAACACGTTTTTTCGCCAATCGAGGAAGCCAGGAGGCTAGCAACAGTCTTCATTCAAC

AmpR

8,900 8,920 8,940 8,960 8,980

ScaI

GCCGCAGTGTTATCACTCATGGTTATGGCAGCACTGCATAATTCTCTTACTGTCATGCCATCCGTAAGATGCTTTTCTGTGACTGGTGAGTACTCAACCAAGTCATT
CGGCGTCACAATAGTGAGTACCAATACCGTCGTGACGTATTAAGAGAATGACAGTACGGTAGGCATTCTACGAAAAGACACTGACCACTCATGAGTTGGTTCAGTAA

AmpR

9,000 9,020 9,040 9,060 9,080

BcgI

CTGAGAATAGTGTATGCGGCGACCGAGTTGCTCTTGCCCGGCGTCAACACGGGATAATACCGCGCCACATAGCAGAACTTTAAAAGTGCTCATCATTGGAAAACGTT
GACTCTTATCACATACGCCGCTGGCTCAACGAGAACGGGCCGCAGTTGTGCCCTATTATGGCGCGGTGTATCGTCTTGAAATTTTCACGAGTAGTAACCTTTTGCAA

AmpR

9,100 9,120 9,140 9,160 9,180 9,200

CTTCGGGGCGAAAACTCTCAAGGATCTTACCGCTGTTGAGATCCAGTTCGATGTAACCCACTCGTGCACCCAACTGATCTTCAGCATCTTTTACTTTCACCAGCGTT
GAAGCCCCGCTTTTGAGAGTTCCTAGAATGGCGACAACTCTAGGTCAAGCTACATTGGGTGAGCACGTGGGTTGACTAGAAGTCGTAGAAAATGAAAGTGGTCGCAA

9,220 9,240 9,260 9,280 9,300

TCTGGGTGAGCAAAAACAGGAAGGCAAAATGCCGCAAAAAAGGGAATAAGGGCGACACGGAAATGTTGAATACTCATACTCTTCCTTTTTCAATATTATTGAAGCAT
AGACCCACTCGTTTTTGTCCTTCCGTTTTACGGCGTTTTTTCCCTTATTCCCGCTGTGCCTTTACAACTTATGAGTATGAGAAGGAAAAAGTTATAATAACTTCGTA

9,320 9,340 9,360 9,380 9,400

ZraI
AatII

TTATCAGGGTTATTGTCTCATGAGCGGATACATATTTGAATGTATTTAGAAAAATAAACAAATAGGGGTTCCGCGCACATTTCCCCGAAAAGTGCCACCTGACGTCT
AATAGTCCCAATAACAGAGTACTCGCCTATGTATAAACTTACATAAATCTTTTTATTTGTTTATCCCCAAGGCGCGTGTAAAGGGGCTTTTCACGGTGGACTGCAGA

9,420 9,440 9,460 9,480 9,500 9,520

AAGAAACCATTATTATCATGACATTAACCTATAAAAATAGGCGTATCACGAGGCCCTTTCGTCTTCAAGAATTAATTCTCATGTTTGACAGCTTATCATCGATAAGC
TTCTTTGGTAATAATAGTACTGTAATTGGATATTTTTATCCGCATAGTGCTCCGGGAAAGCAGAAGTTCTTAATTAAGAGTACAAACTGTCGAATAGTAGCTATTCG

9,540 9,560 9,580 9,600 9,620

TGACTCATGTTGGTATTGTGAAATAGACGCAGATCGGGAACACTGAAAAATAACAGTTATTATTCG
ACTGAGTACAACCATAACACTTTATCTGCGTCTAGCCCTTGTGACTTTTTATTGTCAATAATAAGC

9,640 9,650 9,660 9,670 9,680 9,690
